# Engineering Ferrimagnetic Interactions in Molecular Quantum Systems

**DOI:** 10.1002/anie.4744683

**Published:** 2026-09-15

**Authors:** Elia Turco, Fupeng Wu, Annika Bernhardt, Nils Krane, Ji Ma, Roman Fasel, Michal Juríček, Xinliang Feng, Pascal Ruffieux

**Affiliations:** ^1^ nanotech@surfaces Laboratory Empa – Swiss Federal Laboratories for Materials Science and Technology Dübendorf Switzerland; ^2^ Present address: QuTech and Kavli Institute of Nanoscience Delft University of Technology Delft The Netherlands; ^3^ Max Planck Institute of Microstructure Physics Weinberg Halle Germany; ^4^ Center for Advancing Electronics Dresden (CFAED) & Faculty of Chemistry and Food Chemistry Technische Universität Dresden Dresden Germany; ^5^ Department of Chemistry University of Zurich Zurich Switzerland; ^6^ College of Materials Science and Optoelectronic Technology & Center of Materials Science and Optoelectronics Engineering University of Chinese Academy of Science Beijing China; ^7^ Beijing National Laboratory for Molecular Science, CAS Key Laboratory for Organic Solids, Institute of Chemistry Chinese Academy of Sciences Beijing China; ^8^ Department of Chemistry, Biochemistry and Pharmaceutical Sciences University of Bern Bern Switzerland

**Keywords:** ferrimagnetism, Heisenberg model, physics, quantum, qubit, scanning probe microscopy, scanning tunneling microscope, spin states

## Abstract

Achieving long‐range ferrimagnetic order in purely organic systems remains a major challenge in molecular magnetism. Here, we report the synthesis and characterization of heterospin‐coupling motifs, formed by covalently linking spin‐1/2 and spin‐1 triangular nanographenes. A combined solution‐phase and on‐surface synthetic strategy yields three distinct compounds, whose structures are elucidated by bond‐resolved scanning probe microscopy. Starting from a spin‐1/2–spin‐1 dimer as the elemental ferrimagnetic unit, we employ inelastic electron tunneling spectroscopy to resolve low‐energy magnetic excitations and extract the parameters of the Heisenberg Hamiltonian. Extension to trimeric architectures results in two distinct spin configurations, with compensated (S=0) and uncompensated (S=3/2) ferrimagnetic ground states. The Heisenberg model accurately describes all magnetic transitions, offering direct insight into increasingly complex spin Hamiltonians. These findings establish a molecular platform for designing heterospin systems with robust exchange interactions, providing access to higher‐dimensional spin states beyond the two‐level qubit paradigm.

## Graphical abstract

Heterospin nanographene architectures—constructed by covalently linking spin‐1/2 and spin‐1 triangular units via on‐surface synthesis—are characterized by bond‐resolved scanning probe microscopy. Inelastic electron tunneling spectroscopy probes exchange interactions within a Heisenberg model, revealing both compensated (S=0) and uncompensated (S=3/2) ferrimagnetic ground states.

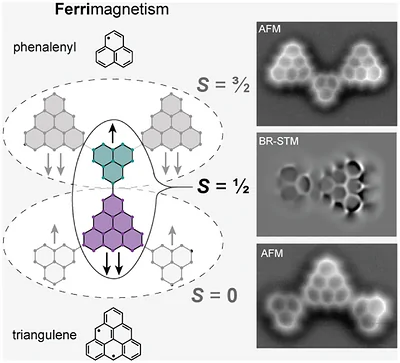

## 1 Introduction

Organic magnetic materials are an emerging platform for spintronics and quantum computing, enabling quantum–state control at the molecular scale [1, 2, 3, 4]. Their tunable structure–quantum properties [5], longer spin coherence times [6], and potential for sustainable production [7] make them attractive alternatives to inorganic magnetic systems. However, their practical deployment is hindered by a prevailing tendency toward antiferromagnetic spin coupling, often resulting in complete spin compensation and zero net magnetization. Ferrimagnetic coupling—where spins of unequal multiplicities align antiferromagnetically to yield a net magnetic moment [8]—offers a compelling alternative, combining the fast spin dynamics and transport characteristics of antiferromagnets with magnetic field addressability akin to ferromagnets [9, 10, 11].

Since Buchachenko's seminal proposal in 1979 [12], the quest for purely organic ferrimagnets has remained a central challenge in molecular magnetism. While long‐range ferrimagnetic order is well established in metal–organic systems [13, 14, 15, 16], fully organic analogues remain elusive, limited by weak exchange interactions, low ordering temperatures, and stability problems [17, 18, 19, 20, 21, 22].

Graphene‐based π‐electron magnets represent a promising route to overcome these limitations, offering strong and tunable exchange couplings (up to hundreds of meV) [23, 24, 25], along with precise control over spin states and molecular architecture [26, 27, 28]. On‐surface synthesis of open‐shell nanographenes on coinage metals has enabled the construction of molecular quantum spin chains, including homospin S=1 and S=1/2 architectures [29, 30, 31, 32], offering an ideal platform to explore quantum magnetism and topological phases in low‐dimensional systems.

Here, we take a first step toward extending this strategy to heterospin systems [33, 34] by synthesizing three model compounds featuring an alternating spin‐1/spin‐1/2 motif.

In contrast to the extensively studied homospin configurations [35, 36, 37, 38, 39, 40], heterospin coupling remains largely unexplored, with previous on‐surface realizations limited to atomically precise metal atom assemblies [41, 42, 43, 44] or metal–organic hybrids on surfaces [45, 46]. To realize a heterospin coupling motif in a purely organic framework, we combine two nanographene building blocks with distinct spin multiplicities. This strategy exploits the bipartite structure of the graphene honeycomb lattice, where sublattice imbalance dictates the total spin quantum number as $S=(N_{A}-N_{B})/2$ [47, 48].

The two smallest members of the triangulene family—namely phenalenyl (2T, S=1/2) and [3]triangulene (3T, S=1)—are herein employed as magnetic building blocks (Scheme 1). Covalent bonding at the β‐positions (minority sublattices) of 2T and 3T yields strong antiferromagnetic exchange, mediated by third‐nearest‐neighbor hopping ($t_{3}$) [36, 49]. The 2T–3T dimer (**1**, Scheme 2) represents the fundamental ferrimagnetic unit, synthesized via a combined solution‐phase and on‐surface synthesis on Au(111). Using the same synthetic strategy, we obtained two trimeric compounds: 3T–2T–3T (**2**) and 2T–3T–2T (**3**), featuring quartet and singlet ground states, respectively. A combination of low‐temperature (4.5 K) scanning tunneling microscopy (STM), atomic force microscopy (AFM), and scanning tunneling spectroscopy (STS) reveals spin excitations in **1**–**3** that are accurately captured by a minimal Heisenberg model [50]. These findings establish a modular platform for engineering all‐carbon ferrimagnets and underscore their potential as molecular qudits in quantum information applications [2, 51, 52, 53].

**SCHEME 1:**
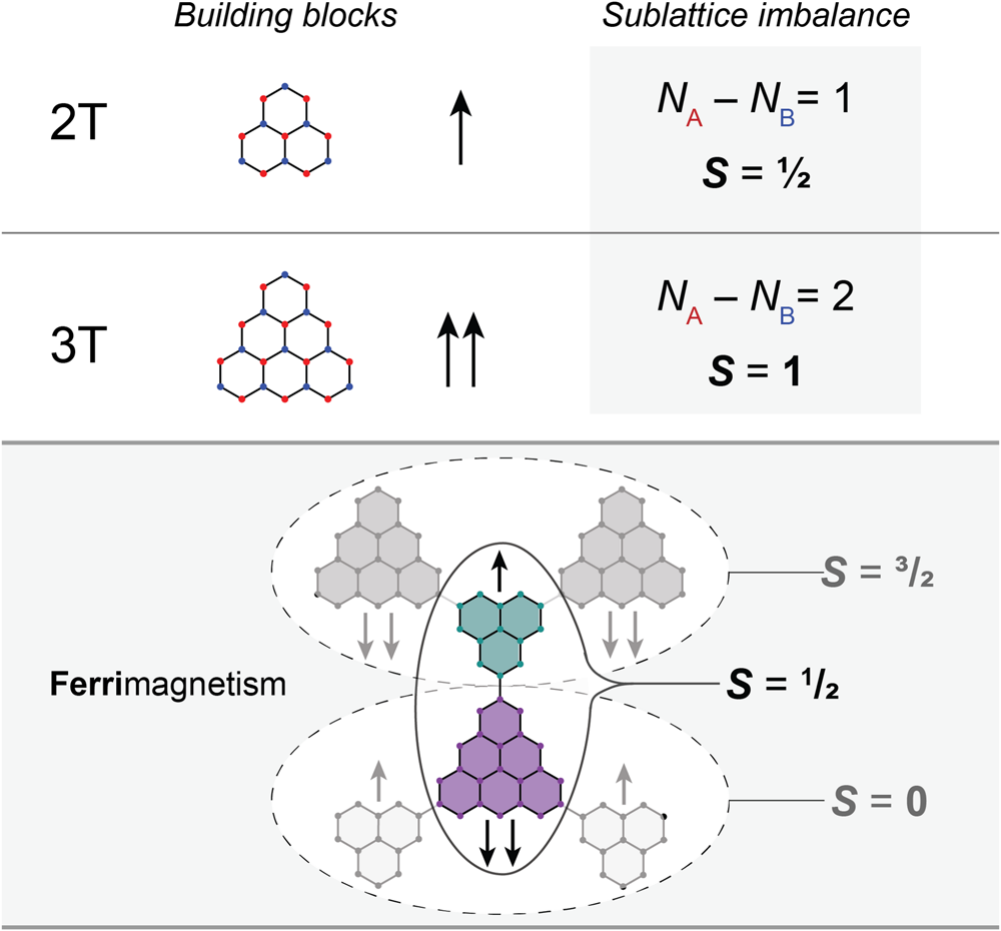
Phenalenyl (2T) and [3]triangulene (3T) as magnetic building blocks with total spin quantum numbers S=1/2 and S=1, respectively. The total spin S arises from the sublattice imbalance, as described by the Ovchinnikov rule [47, 48], $S=(N_{A}-N_{B})/2$, where $N_{A}$ and $N_{B}$ are the number of atomic sites in sublattices A (red) and B (blue). Covalent heterospin coupling of 2T and 3T units into dimers and trimers enables the realization of distinct ferrimagnetic ground states.

**SCHEME 2:**
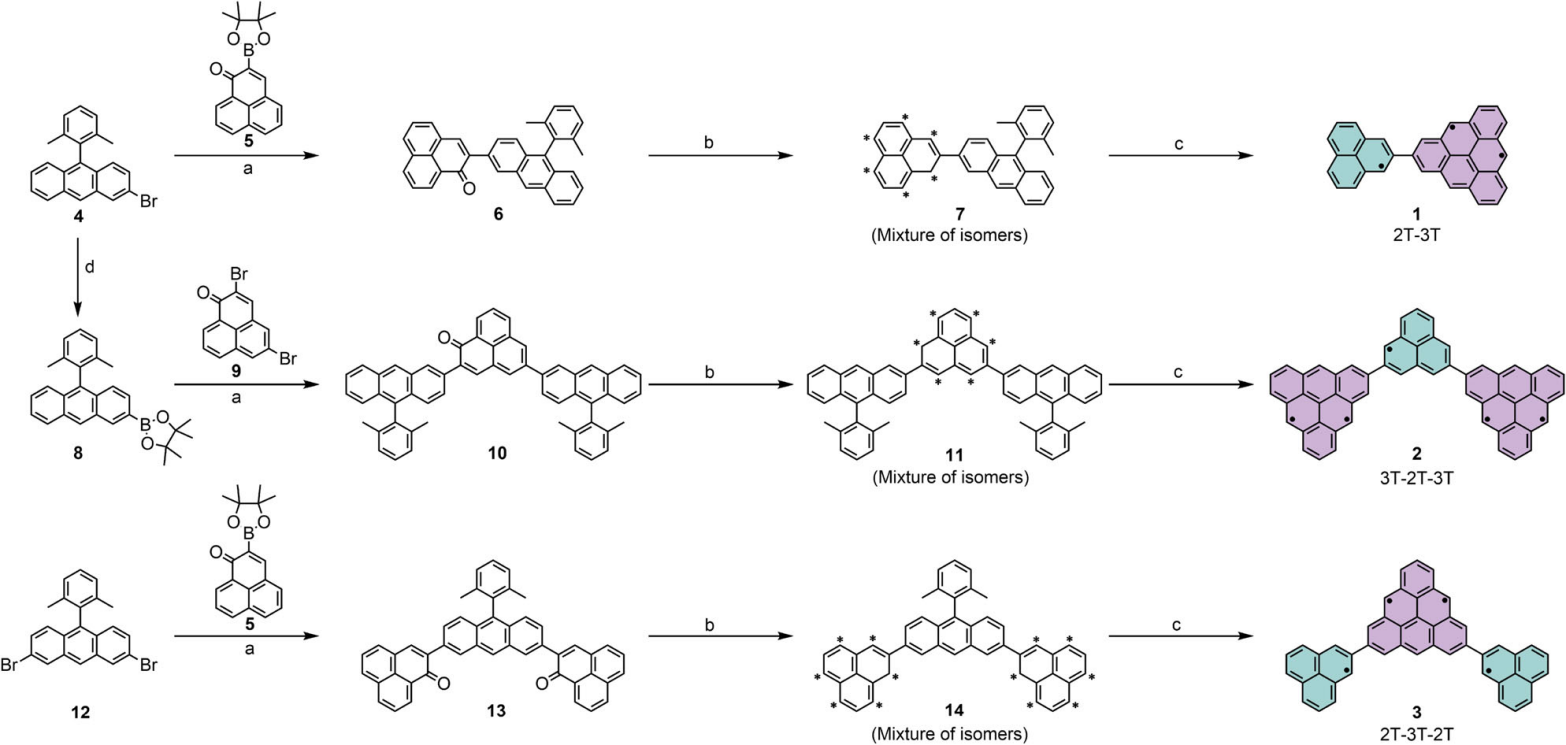
Synthetic route to phenalenyl–triangulene dimer and trimers (compounds **1**–**3**). Reagents and conditions: (a) Pd(dppf)${\mathrm{Cl}}_{2}\cdot {\mathrm{CH}}_{2}{\mathrm{Cl}}_{2}$, K_3_PO_4_, 1,4‐dioxane, 85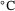, 16 h, yields: 90% and 89% for compounds **6** and **13**, respectively; Pd(PPh_3_)_4_, K_2_CO_3_, 1,4‐dioxane/H_2_O, 85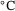, overnight, yield: 79% for compound **10**; (b) DIBAL‐H, toluene, rt or 100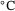, overnight, yields: 32%, 68%, and 72% for compounds **7**, **11**, and **14**, respectively; (c) On Au(111), 320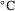; (d) 4,4,4',4',5,5,5',5'‐octamethyl‐2,2'‐bi(1,3,2‐dioxaborolane), Pd(OAc)_2_, SPhos, K_3_PO_4_, 1,4‐dioxane, 90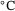, overnight, 92%. *Note: In a mixture of positional isomers, the methylene groups occupy any* α *‐position* *(marked with asterisks) of the phenalene subunit*.

## 2 Results and Discussion

The 2T–3T dimer (**1**), 3T–2T–3T trimer (**2**), and 2T–3T–2T trimer (**3**) were synthesized via surface‐assisted cyclodehydrogenation of molecular precursors **7**, **11**, and **14**. These precursors were obtained via solution‐phase synthesis in two steps, as illustrated in Scheme 2 (detailed procedures and characterization data are provided in the Supporting Information). In the first step, a Suzuki coupling reaction between 2‐bromo‐10‐(2,6‐dimethylphenyl)anthracene (**4**) and 2‐(4,4,5,5‐tetramethyl‐1,3,2‐dioxaborolan‐2‐yl)‐1*H*‐phenalen‐1‐one (**5**) afforded 2‐(10‐(2,6‐dimethylphenyl)anthracen‐2‐yl)‐1*H*‐phenalen‐1‐one (**6**) in 90% yield. Subsequent reduction of **6** with diisobutylaluminum hydride (DIBAL‐H) yielded the 2T–3T precursor **7** as a mixture of positional isomers where the methylene groups can occupy any α‐position (marked with asterisks) of the phenalene subunit. Following a similar strategy, Suzuki coupling between 2‐(10‐(2,6‐dimethylphenyl)anthracen‐2‐yl)‐4,4,5,5‐tetramethyl‐1,3,2‐dioxaborolane (**8**) and 2,5‐dibromo‐1*H*‐phenalen‐1‐one (**9**) afforded 2,5‐bis(10‐(2,6‐dimethylphenyl)anthracen‐2‐yl)‐1*H*‐phenalen‐1‐one (**10**) in 79% yield. Reduction of **10** with DIBAL‐H gave **11** as an isomeric mixture, which was used as the precursor for trimer **2**. Similarly, Suzuki coupling of compound **12** with compound **5** afforded 2,2'‐(10‐(2,6‐dimethylphenyl)anthracene‐2,7‐diyl)bis(1*H*‐phenalen‐1‐one) (**13**) in 89% yield. Reduction of **13** with DIBAL‐H gave **14** as an isomeric mixture, which served as the precursor for trimer **3**.

The on‐surface synthesis of **1**, **2**, and **3** was achieved in two steps. First, the precursors **7**, **11**, and **14** were independently deposited onto atomically clean Au(111) surfaces at room temperature; STM analysis of the resulting adsorbed molecular species is provided in Figure S2. Subsequent annealing to 320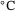 triggered oxidative ring closure of the methyl groups, yielding the 2T–3T dimer **1** and the two trimers 3T–2T–3T (**2**) and 2T–3T–2T (**3**), as shown in the overview STM images in Figure S3.

Single‐molecule STM and bond‐resolved STM/AFM imaging (Figure 1c,b) confirm the successful on‐surface synthesis of target structures **1**–**3**, along with hydro intermediates 2T–H3T and 3T–2T–H3T. The latter likely result from partial passivation of radical centers, resulting in the formal replacement of CH groups with CH_2_ groups, attributed to atomic hydrogen diffusion during annealing. The corresponding chemical structures (Figure 1a) define five distinct spin Hamiltonians, with total spin quantum numbers ranging from S=0 to S=3/2, modeled using the Heisenberg formalism (Figure 1e). To validate the magnetic ground state assignments, we employed a tight‐binding framework with electron correlation treated at the mean‐field Hubbard level (MFH‐TB), and computed the local density of states (LDOS) for each structure.

**FIGURE 1:**
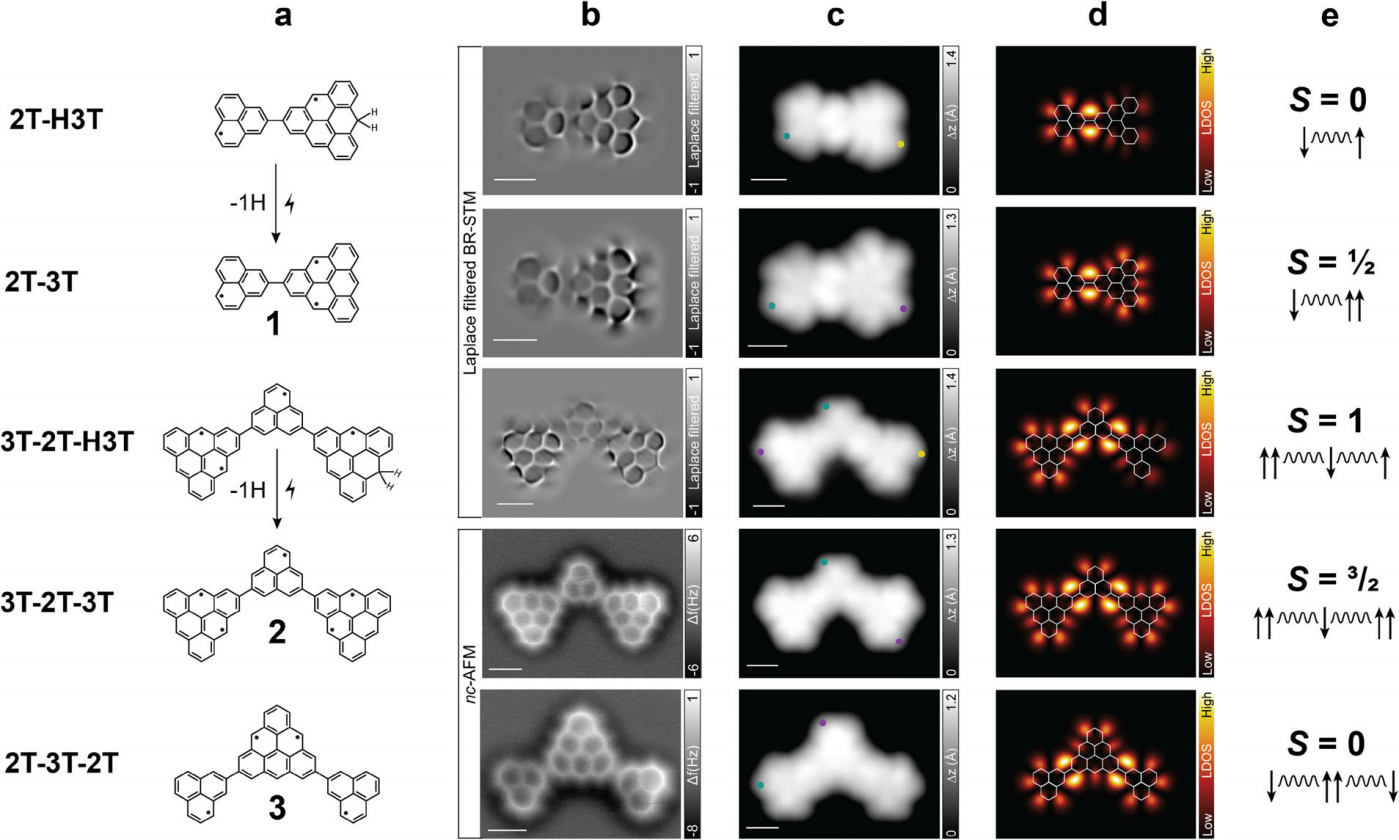
On‐surface synthesis of dimers and trimers composed of covalently coupled 2T and 3T building blocks. (a) Chemical sketch of the nanographenes synthesized and characterized in this work. Molecules labeled as **1**–**3** correspond to the target compounds obtained via thermal annealing of their respective precursors **7**, **11**, and **14**, while 2T–H3T and 3T–2T–H3T are identified as hydro intermediates. (b) Structural characterization of molecules in (a), carried out with a carbon monoxide (CO)‐functionalized tip by using bond‐resolved STM (opened feedback on the molecule with –5 mV/50 pA, Δz=−0.7 Å) or *nc*‐AFM techniques (open‐feedback on Au(111) with −5 mV/100 pA, Δz=1.9‐2.0 Å). (c) High‐resolution STM images, closed feedback with −0.1 V/100–150 pA, acquired with a CO‐functionalized tip. Filled colored circels indicate the positions where the spectra in Figures 2 and 4 were acquired. (d) Calculated MFH‐LDOS maps of the spin‐carrying orbitals for each molecular structure, with molecular skeletons overlaid. The ${\mathrm{CH}}_{2}$ group is incorporated into the MFH model by removing the corresponding carbon site from the π‐system. (e) Total spin quantum number S and the corresponding Heisenberg spin model for structures in (a). Scale bars: 0.5 nm (b,c).

The resulting LDOS maps of spin‐carrying orbitals (Figure 1d) show excellent agreement with the apparent topography of the in‐gap STM images acquired at V=−0.1 V (Figure 1c), reflecting the spatial distribution of the orbitals involved in low‐energy spin excitations. While the following section focuses on the magnetic properties, the electronic structure of **1**, **2**, and **3** was also investigated by detailed STS and MFH‐TB analysis (Figures S4, S7, and S8). The observed features highlight the multireference character of the coupled open‐shell nanographenes (Figure S5), consistent with previous studies on related systems [36, 39, 54]. In particular, the experimentally observed ion resonances originate from multireference electronic states, which arise from the hybridization of the spin‐carrying orbitals, and therefore cannot be uniquely assigned to individual molecular orbitals [55, 56].

### 2.1 Magnetic characterization

We now turn to the low‐bias STS analysis of the structures presented in Figure 1a, with the aim of validating the previously assigned Heisenberg representation (Figure 1e). To this end, we first determine the spin Hamiltonian parameters from the dimeric coupling motifs (Figure 2), and then use the as‐determined values to model the spin excitations observed in the trimeric systems (Figure 4).

**FIGURE 2:**
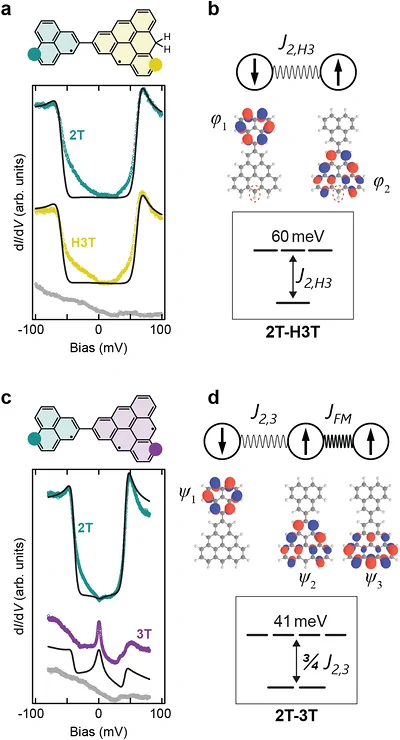
Extraction of spin Hamiltonian parameters from STS spectra. (a) Experimental and simulated differential conductance d*I*/d*V* spectra of 2T–H3T. The spectra were taken at positions marked on the sketch (top) and in Figure 1c. The feedback loop was opened on the molecule at −0.1 V/850 pA (a) and −80 mV/850 pA (b). Lock‐in modulation voltage $V_{m}$ = 1 mV (a,b). The simulated spectra were calculated by a third‐order‐scattering model assuming Heisenberg coupling, as depicted in (b) and described in the Supporting Information. The frontier states corresponding to the spins in the Heisenberg model are shown as iso‐surfaces below. Matching the simulated spectra with the experiment yields an effective coupling of $J_{2,H3}=60\;\text{meV}$. (c,d) Same as (a,b) but for 2T–3T. Assuming the ferromagnetic coupling of the two 3T spins to be much larger than the coupling to 2T [57] ($J_{\text{FM}}\gg J_{2,3}$), the observed doublet–quartet gap corresponds to $\frac{3}{4}J_{2,3}\approx 41\;\text{meV}$, thus $J_{2,3}=54\;\text{meV}$.

Antiferromagnetic coupling of two spin‐1/2 units is realized via the structure 2T–H3T, where the additional hydrogen atom effectively removes one unpaired electron from the triangulene unit, with the remaining unpaired $p_{z}$ electron delocalized over the triangulene backbone. The spatial distribution of the resulting spin‐carrying orbitals, $\varphi_{1}$ and $\varphi_{2}$, obtained from TB‐MFH calculations, is shown in Figure 2b. Low‐bias STS spectra acquired on the 2T and H3T units (Figure 2a) reveal two symmetric steps around the Fermi level, corresponding to inelastic singlet–triplet excitations. These features are well reproduced by a Heisenberg dimer model, with Hamiltonian $H=J_{2,H3}\;S_{1}\cdot S_{2}$, including spin‐flip processes up to third order [58].

The corresponding calculated spectra, shown in black in the graph, closely reproduce the experimental features with an effective exchange coupling of $J_{2,H3}=60$ meV—significantly larger than values reported for symmetric dimers [36, 59]. To rationalize this, we estimate the effective hopping ($t_{\mathrm{eff}}$) and Coulomb repulsion ($U_{\mathrm{eff}}$), which enter the expression for the exchange coupling as $J=4t_{eff}^{2}/U_{eff}$, using the TB‐MFH model [49]. Although $t_{\mathrm{eff}}$ is only 5% larger than in the 2T–2T dimer [36], a 21% reduction in $U_{\mathrm{eff}}$ for 2T–H3T accounts for the enhanced spin coupling—highlighting the potential of wavefunction engineering to modulate exchange interactions in molecular spin systems [26].

Controlled dehydrogenation of the H3T unit via tip‐induced manipulation [28, 60] yields the 2T–3T dimer (**1**), which serves as the ferrimagnetic unit for the trimeric structures.

The three resulting spin‐carrying orbitals, $\psi_{i}$, are shown in Figure 2d. Notably, the 3T‐localized orbital $\psi_{3}$ does not hybridize with $\psi_{1}$, justifying our spin‐chain‐like model (Figure S1). The STS spectra in Figure 2c exhibit two symmetric steps with higher intensity at the 2T unit, and weaker features along with a zero‐bias resonance at the 3T site. The latter is a hallmark of the degenerate doublet ground state of the 2T–3T system and its spatial distribution coincides with $\psi_{3}$, as evidenced by the constant‐current d*I*/d*V* map on the right‐hand side of Figure 3a‐right. In contrast, the inelastic doublet–quartet spin excitation predominantly localizes at the 2T unit, as revealed in the left part of Figure 3a. Simulated spectra based on the corresponding Heisenberg model (black curves) reproduce the experimental data with an exchange coupling of $J_{2,3}=54$ meV.

**FIGURE 3:**
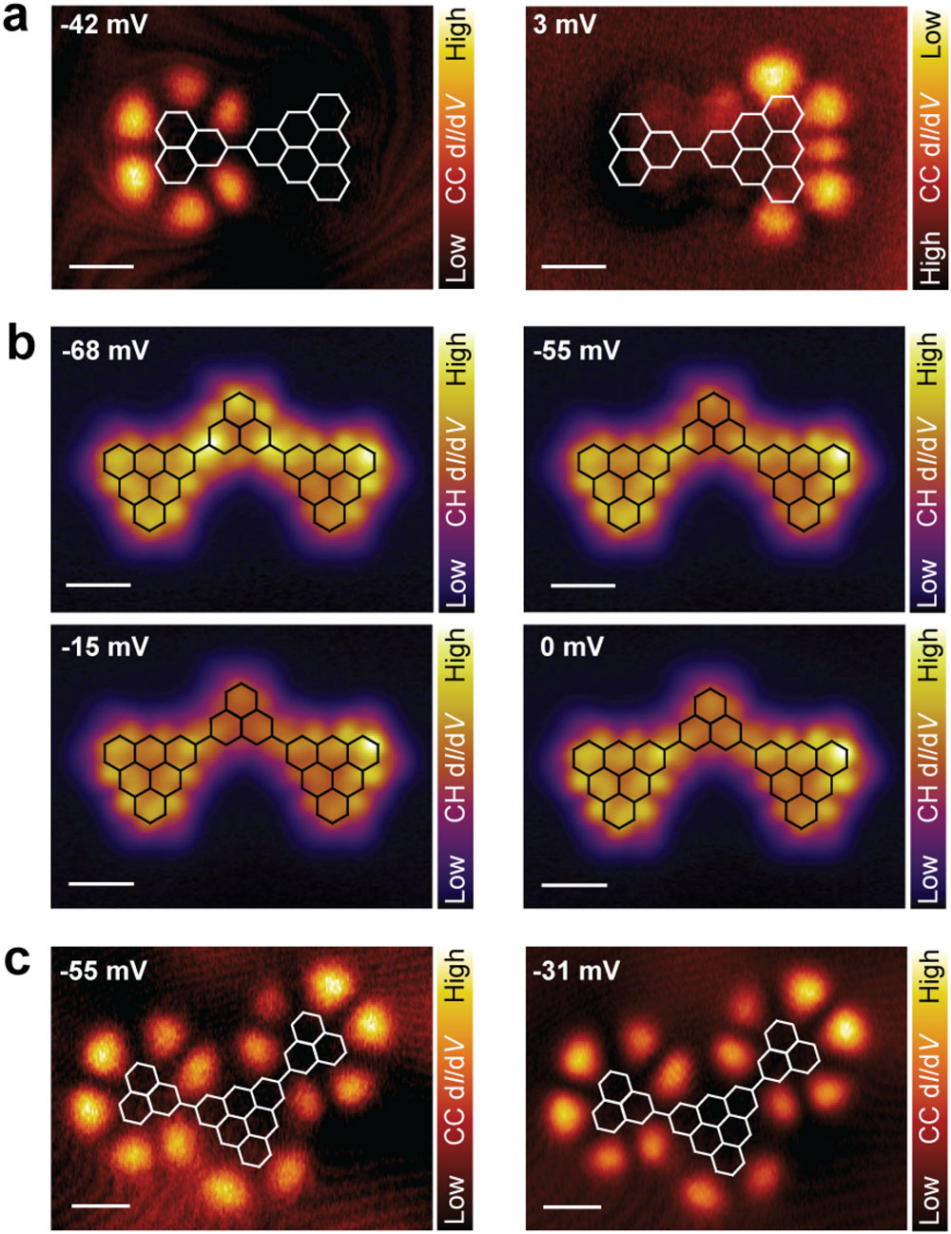
Spin excitation maps of **1**–**3**. (a,c) Constant‐current (CC) maps and (b) constant‐height (CH) maps acquired at the voltage thresholds corresponding to the spin excitation energies (maps at positive biases are provided in the Supporting Information). CC setpoints: (a)‐left 1 nA, (a)‐right 100 pA, (c) 350 pA. CH maps (b): feedback opened on the molecule at −0.1 V/800 pA. For the map on the right of panel (a), depicting the spatial extension of the Kondo‐like resonance, we have leveraged the topographic effect of constant‐current maps. At small finite bias, the presence of a zero‐bias peak leads to tip retraction, thereby reducing the measured d*I*/d*V* intensity. To enhance the visibility of the Kondo resonance, the color scale has been inverted (original map is reported in the Supporting Information). Lock‐in modulation $V_{m}$: (a,c) 2 mV, (b,d) 4 mV. (a,c) were acquired with a metal tip, (b) with a CO‐functionalized tip. Scale bars: 0.5 nm.

We note that both, experimental and theoretical spectra, recorded at the 2T unit reveal a characteristic zero‐bias dip—which is a spectroscopic signature of the ferromagnetic Kondo effect [61].

Having established the coupling constants $J_{2,H3}$ and $J_{2,3}$, we now examine the trimeric structures 3T–2T–H3T, 3T–2T–3T, and 2T–3T–2T, corresponding to total spin ground states of S=1, S=3/2, and S=0, respectively. The results summarized in Figure 4 include a detailed low‐bias STS analysis of the relevant magnetic excitations and a comparison with the corresponding simulated d*I*/d*V* curves.

**FIGURE 4:**
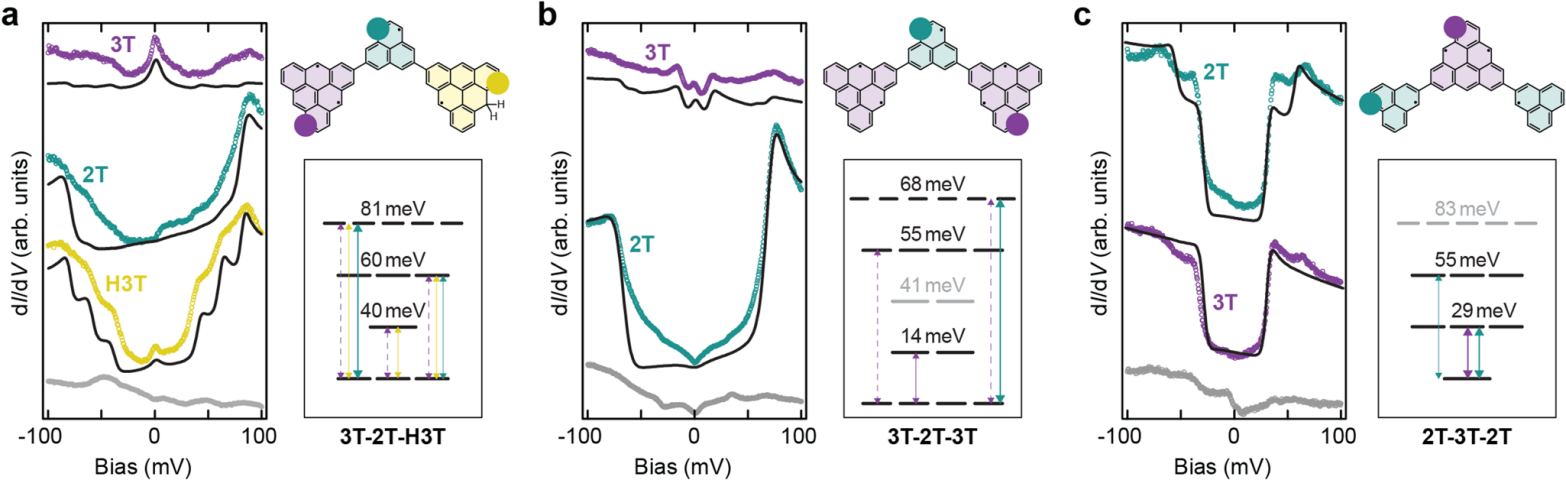
Experimental and calculated IETS spectra for **2** and **3**. (a) Differential conductance spectra taken at three different positions on 3T–2T–H3T, marked as on the molecule sketch and in Figure 1c. The feedback loop was opened on the molecule at −0.1 V/800 pA (a,b) and −0.1 V/500 pA (c). Lock‐in modulation voltage $V_{m}$ = 2 mV (a,b) and $V_{m}$ = 1 mV (c). Black solid lines correspond to simulated spectra using the coupling parameters $J_{2,3}$ and $J_{2,H3}$ as determined before. The energy scheme shows the available spin excited states, whereas the multiplicity is represented by the number of lines. Colored arrows indicate the excitation intensity, depending on tip position.

The magnetic spectrum of 3T–2T–H3T (Figure 4a) shows inelastic transitions from the triplet ground state to singlet, triplet, and quintet states, which are well reproduced by the calculated d*I*/d*V* spectra using the previously determined exchange couplings $J_{2,H3}$ and $J_{2,3}$.

Figure 4b and Figure 4c show low‐bias STS data of 3T–2T–3T (**2**) and 2T–3T–2T (**3**), respectively. In 3T–2T–3T, asymmetric spin coupling yields an uncompensated spin‐3/2 ground state, with excitations to quartet and sextet states clearly resolved in the d*I*/d*V* spectra (green and purple traces). Constant‐height d*I*/d*V* maps (Figure 3b) reveal the spatial distribution of these excitations: the quartet–sextet transition localizes on the 2T unit, while transitions to doublet and quartet states appear at the 3T sites. As in the 2T–3T dimer, the trimer exhibits signs of both ferromagnetic and overscreened behavior, evidenced by a zero‐bias peak at the 3T sites and a dip at the 2T site (Figure 4b).

In contrast, the 2T–3T–2T trimer, with two 2T units coupled to a central 3T, is in a fully compensated singlet ground state (S=0). STS spectra (Figure 4c) reveal two distinct inelastic excitations to triplet states, at 29 and 55 meV. Experimentally, both excitations appear at the 2T and 3T sites, whereas the Heisenberg model (see Supporting Information) predicts the 55 meV transition to be localized only at the 2T units. This discrepancy may arise from the simplified assumption that only one of the 3T's two degenerate zero modes couples to the neighboring 2T. While valid for dimers, this picture appears to break down in the symmetric 2T–3T–2T trimer, where both 2T units can hybridize with the 3T. This necessitates a more refined model of the coupling mechanism [29, 62].

## 3 Conclusion

We have demonstrated an antiferromagnetic heterospin coupling motif as a robust strategy for engineering complex spin Hamiltonians in all‐carbon systems. Through in‐solution and on‐surface synthesis, we covalently couple S=1/2 and S=1 triangular nanographenes to construct three distinct ferrimagnetic configurations. Tip‐induced dehydrogenation provides an additional tuning knob to tailor the magnetic properties of the resulting π‐conjugated topologies, enabling access to all ground states from S=0 to S=3/2. High‐resolution STS of the dimeric units, serving as elemental coupling motifs, yields Heisenberg parameters that accurately reproduce the magnetic excitations of the more complex trimer structures, validating the underlying spin model. The resulting spin Hamiltonians feature a rich manifold of spin multiplets and excitations, exemplifying prototypical multilevel quantum systems with tunable and well‐defined spin states.

These results establish a bottom‐up route to tailored spin architectures and provide a foundation for realizing 1D and 2D non‐centrosymmetric lattices, where broken symmetry is predicted to stabilize ferrimagnetic ground states and correlated spin phases [63, 64].

## Conflicts of Interest

The authors declare no conflicts of interest.

## Supporting information


**Supporting File**: anie74668‐sup‐0001‐SuppMat.pdf.

## Data Availability

The raw NMR data are freely available on Zenodo at https://zenodo.org/record/15603117 (DOI: 10.5281/zenodo.15603117).
