# Supplementary material for: Engineering Ferrimagnetic Interactions in Molecular Quantum Systems
Source: Angew Chem Int Ed Engl. 2026 Sep 15;65(44):e4744683. doi: 10.1002/anie.4744683 (PMC13703890; doi:10.1002/anie.4744683)
Supplement: Supplementary file 1 [file ANIE-65-e4744683-s001.pdf]

Supporting Information for: Engineering Ferrimagnetic Interactions in Molecular Quantum Systems

Elia Turco,[*,a,†] Fupeng Wu,[b,†] Annika Bernhardt,[c,†] Nils Krane,[a] Ji Ma,[d] Roman Fasel,[a,e] Michal Juríček,[*,c] Xinliang Feng,[*,b] Pascal Ruffieux[*,a]

Data Availability

The raw NMR data are freely available on Zenodo at https://zenodo.org/record/15603117 (DOI: 10.5281/zenodo.15603117).

1. Experimental Methods

1.1. Sample Preparation and Scanning Probe Measurements

STS measurements were performed with a commercial low-temperature STM/AFM from Scienta Omicron operated at a temperature of 4.5 K and a base pressure below $5 \cdot 10^{-11}$ mbar. The Au(111) single crystal surfaces were prepared by iterative Ar^+ sputtering and annealing cycles. Before sublimation of molecules, the surface quality was verified through STM imaging. The powders of **7** and **11** precursors were filled into quartz crucibles of a home-built evaporator and sublimed at 210 ° C and 300 °C, respectively, on the surfaces of the single crystal. Instead, the molecular precursor **14** was sublimed via direct current heating of a Silicon wafer. STM images were acquired in both constant-current (overview and high-resolution imaging) and constant-height (bond-resolved imaging) modes, $\mathrm{d}I/\mathrm{d}V$ spectra were acquired in constant-height mode, and $\mathrm{d}I/\mathrm{d}V$ maps were acquired in constant-current mode. Indicated bias voltages are given with respect to the sample. Unless otherwise noted, all measurements were performed with metallic tips. Differential conductance $\mathrm{d}I/\mathrm{d}V$ spectra and maps were obtained with a lock-in amplifier. Modulation voltages for each measurement are provided in the respective figure caption. Bond-resolved STM and *nc*-AFM images were acquired in constant-height mode with CO-functionalized tips at low bias voltages while recording the current signal. Open feedback parameters on the molecular species and the subsequent lowering of the tip height (Δz) for each image are provided in the respective figure captions. The data was processed with Wavemetrics Igor Pro software.

1.2. Hydrogen passivation

In figure 1 of the main text, dihydro intermediates of **1** and **2** are reported. These structures are formed by passivation of the *active* spin sites by atomic hydrogen diffusing on the metal surface, subsequently to the cyclodehydrogenation reactions. Therefore, it naturally occurs to find structures whose unpaired electrons are quenched by CH_2 groups, thus allowing the detection and sequential manipulation of intermediates with various spin ground states S. More details on the tip-based manipulation method can be found in Ref. 1 and Ref. 2.

2. Computational Methods

2.1. Tight-binding and mean-field Hubbard calculations

TB-MFH calculations were performed by numerically solving the mean-field Hubbard Hamiltonian with third-nearest-neighbor hopping.

The corresponding Hamiltonian reads as

$$\hat{\mathcal{H}}_{\mathrm{MFH}} = \sum_{j} \sum_{\langle\alpha,\beta\rangle j,\sigma} t_j \hat{c}^{\dagger}_{\alpha,\sigma} \hat{c}_{\beta,\sigma} + U \sum_{\alpha,\sigma} \langle n_{\alpha,\sigma}\rangle n_{\alpha,\bar{\sigma}} - U \sum_{\alpha} \langle n_{\alpha,\uparrow}\rangle \langle n_{\alpha,\downarrow}\rangle, \tag{1}$$

Here, $c_{\alpha,\sigma}{}^{\dagger}$ and c_{β}, σ denote the spin selective ($\sigma \in \uparrow, \downarrow$) creation and annihilation operator at sites α and β, $\langle \alpha, \beta \rangle_j$ ($j = 1, 3$) denotes the nearest-neighbor and third-nearest-neighbor sites for j = 1, and 3, respectively, t_j denotes the corresponding hopping parameters (with $t_1 = 2.7$ eV and $t_3 = 0.1t_1$ for nearest-neighbor and third-nearest-neighbor hopping), U denotes the on-site Coulomb repulsion, $n_{\alpha,\sigma}$ denotes the number operator, and $\langle n_{\alpha,\sigma} \rangle$ denotes the mean occupation number at site α. Orbital electron densities, ρ, of the nth-eigenstate with energy E_n have been simulated from the corresponding state vector $a_{n,i,\sigma}$ by

$$\rho_{n,\sigma}(\vec{r}) = \left| \sum_{i} a_{n,i,\sigma} \phi_{2p_z}(\vec{r} - \vec{r_i}) \right|^2, \tag{2}$$

where i denotes the atomic site index and ϕ_{2p_z} denotes the Slater $2p_z$ orbital for carbon. All TB-MFH calculations presented in the manuscript were done in the third-nearest-neighbor approximation and using an on-site Coulomb term $U = 1.2\,|t_1|$.

2.2. Calculation of differential conductance spectra

First, the spin Hamiltonian is constructed by a chain of spin-1/2 units, whereas each triangulene unit (3T) is considered to consist of two S=1/2 spins, coupled by a ferromagnetic Heisenberg-like exchange $J_{\mathrm{FM}} = -1\,\mathrm{eV}$. The phenalenyl radical (2T) and the hydrogenated triangulene (H3T) are represented by a single spin-1/2. The units are coupled by one of the two coupling parameters $J_{2,3} = 54\,\mathrm{meV}$ between 2T and 3T and $J_{2,H3} = 60\,\mathrm{meV}$ between 2T and H3T.

The $\mathrm{d}I/\mathrm{d}V$ spectra were simulated by introducing a perturbative spin scattering term into the Hamiltonian, which accounts for spin-flip processes up to third order in the interaction matrix elements [3].

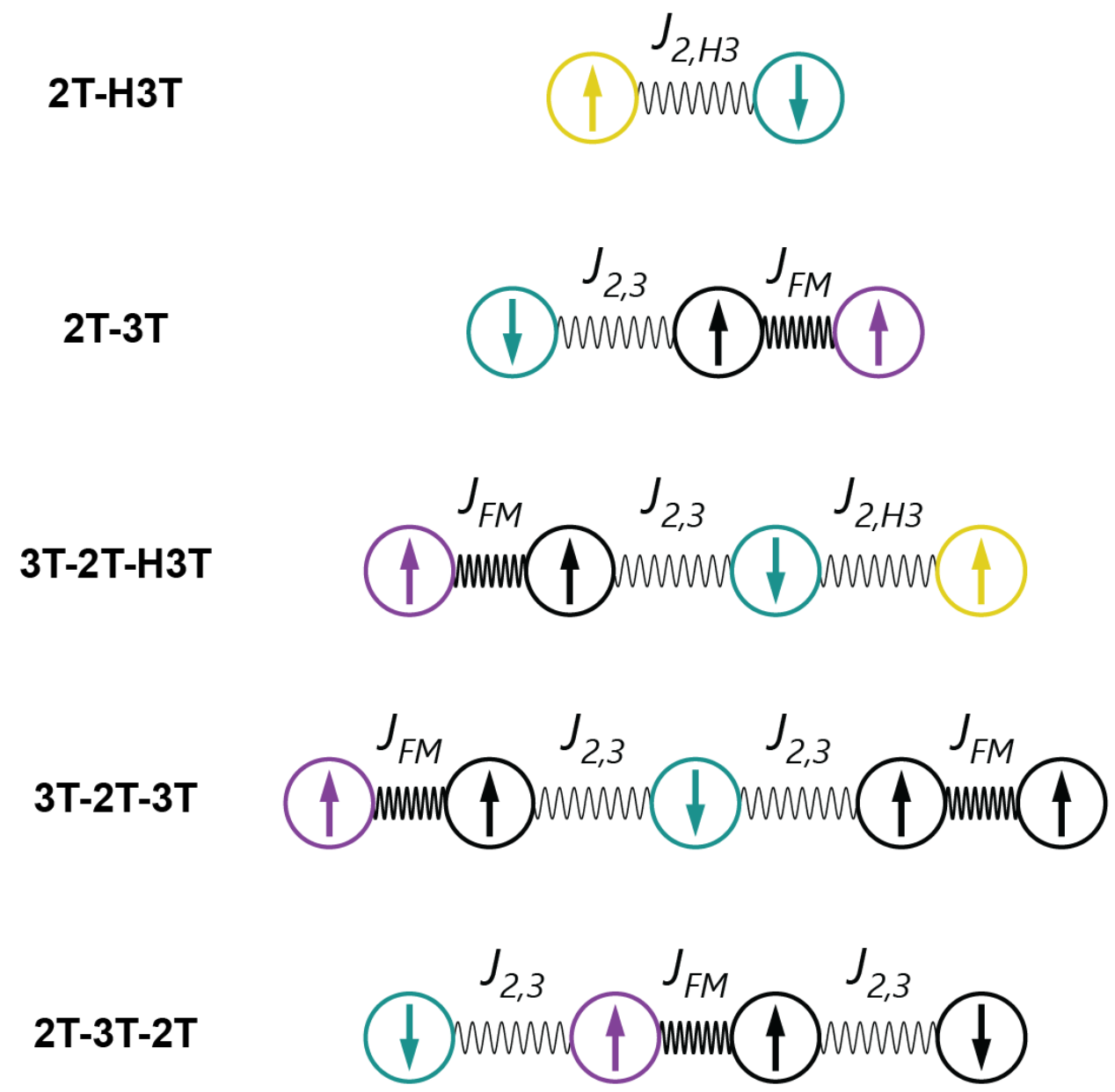

Figure S1. Sketch of Heisenberg models used for simulation of differential conductance spectra. Each unit represents an S=1/2 spin, coupled by one of three different coupling strengths to the neighbor. The colored units indicate the considered spins for the respective spectra, shown in Figures 2 and 4 of the main text.

Table 1

	2T-H3T		2T-3T		3T-2T-H3T			3T-2T-3T		2T-3T-2T	
Parameter	H3T	2T	2T	3T	3T	2T	H3T	3T	2T	2T	3T
U	-0.1	-0.1	-0.08	0	0	-0.1	-0.1	0	-0.2	-0.1	-0.1
$J\rho$	-0.3	-0.3	-0.25	-0.3	-0.2	-0.4	-0.4	-0.2	-0.3	-0.2	-0.2
T	30	30	20	15	15	25	15	15	30	15	15
x_0	-1.15	-1.15	-1.15	-0.15	-1.15	-1.15	-1.15	-1.15	-1.15	-1.15	-1.15
slope	-0.1	-0.1	-0.1	-0.4	0	0.4	0.2	-0.4	0.2	-0.3	-0.3

Table 1 summarizes the parameter used to simulate the $\mathrm{d}I/\mathrm{d}V$ shown in the main text: U is the asymmetry parameter for broken electron–hole symmetry, $J\rho$ the coupling strength to the substrate, T the temperature in Kelvin and x_0 the x-offset in meV (see Ref. 3).

3. Additional experimental data

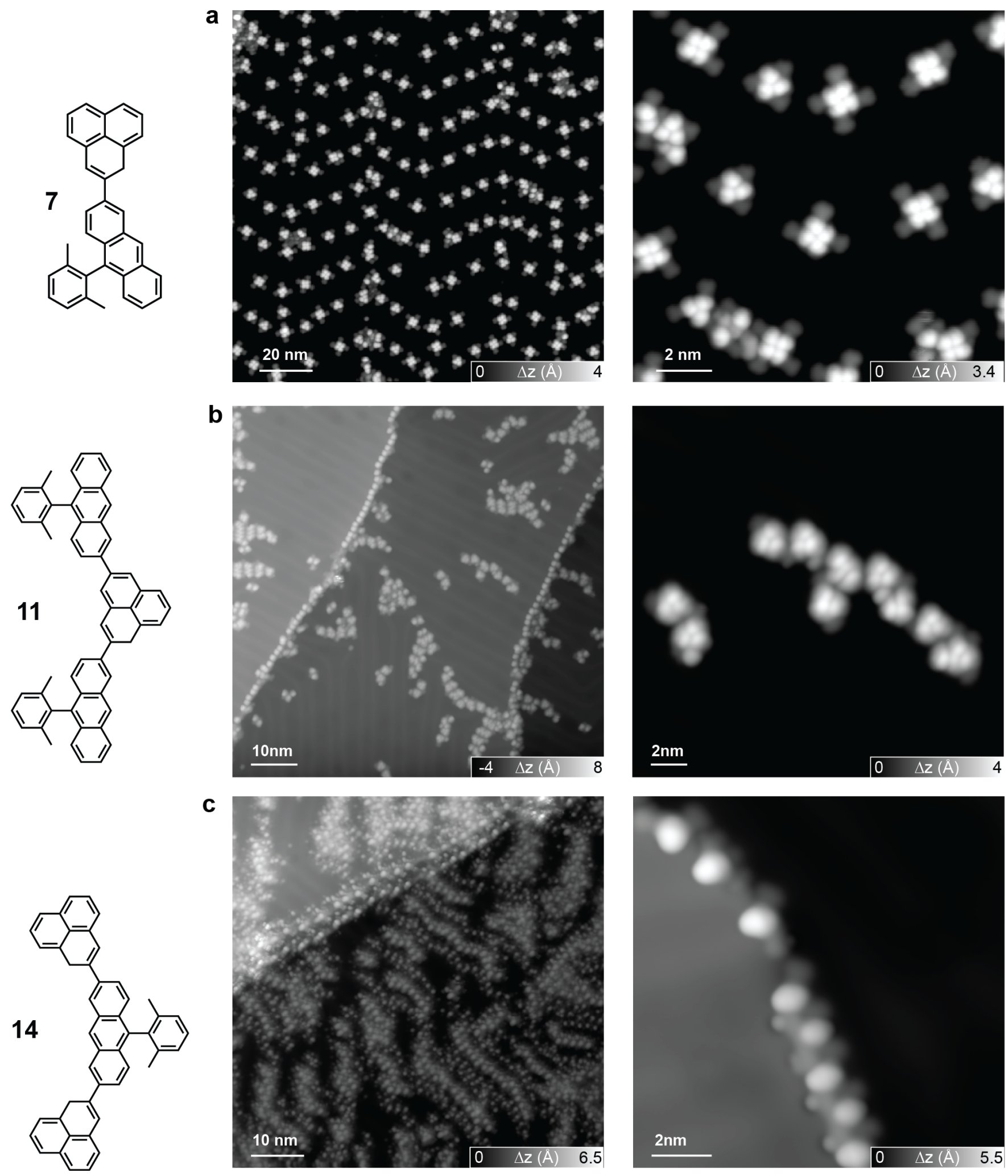

Figure S2. Molecular precursors deposited on Au(111) at room temperature. From left to right: chemical structures of precursors **7**, **11**, and **14**; (a–c) overview STM images showing submonolayer coverage; high-resolution STM images (right) of the corresponding self-assembled molecular clusters. Tunneling parameters: -1 V/30 pA (a,c-left), -1.5 V/30 pA (b), (c-right) -0.4 V/50 pA. The ambiguous appearance of (c-left) is due to a double-tip effect.

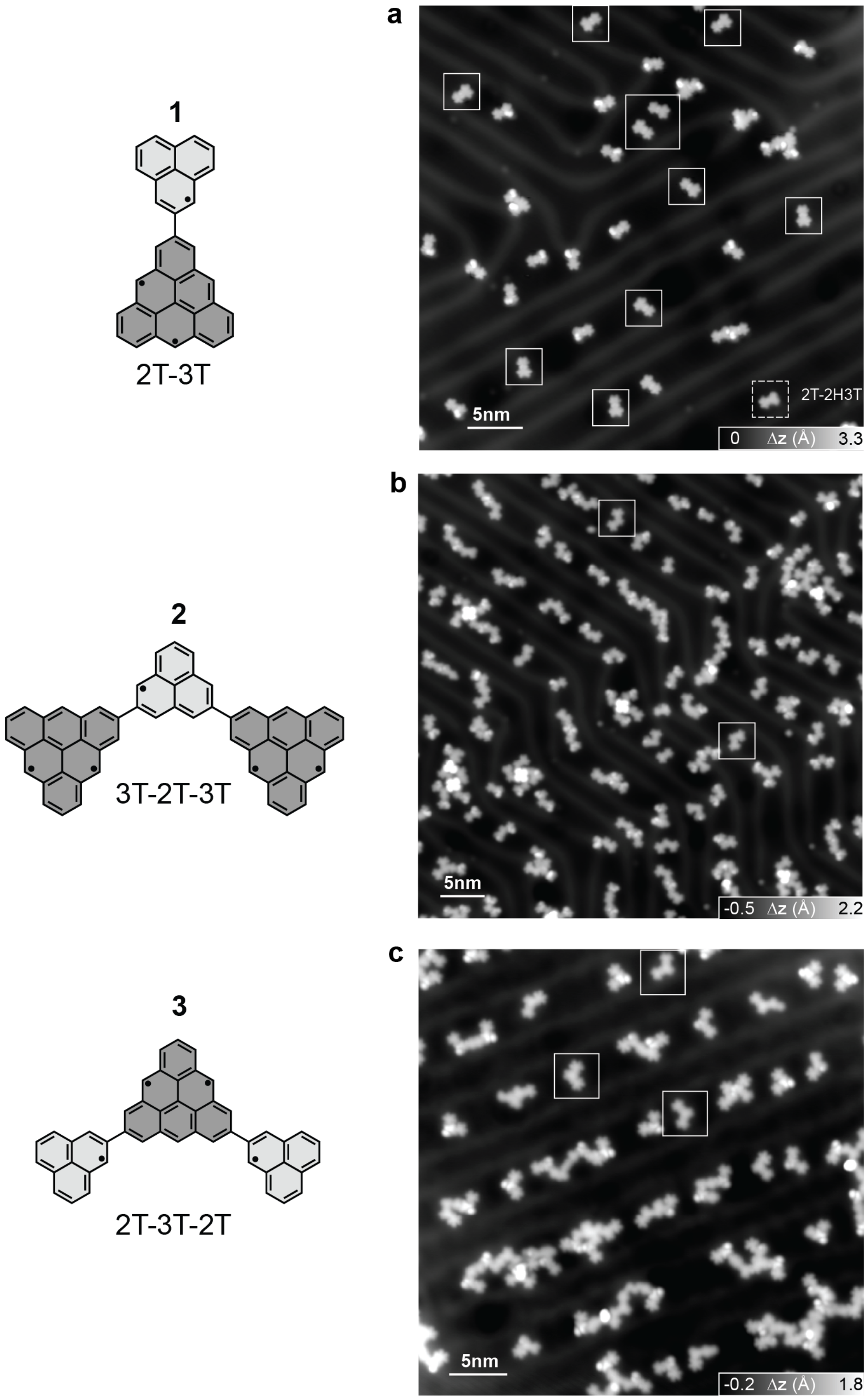

Figure S3. Thermally assisted on-surface synthesis towards target compounds **1**-**3**. Starting from the submonolayer coverage of the molecular precursors (described in Figure S2), each sample was annealed to 320°C for few minutes to trigger the oxidative ring closure of the methyl groups. (a,b,c) STM images acquired subsequently to the annealing step, starting from precursors **7**, **11** and **14**. In each STM image, the corresponding target structures are highlighted with white frames. Notably, the yield of target molecules is higher for **1** than for **2** and **3**. Along with the target structures we always observe a coexistence of covalently coupled molecular clusters and unreacted molecular precursors. Tunneling parameters: -1 V/50 pA (a,b), -0.1 V/50 pA (c).

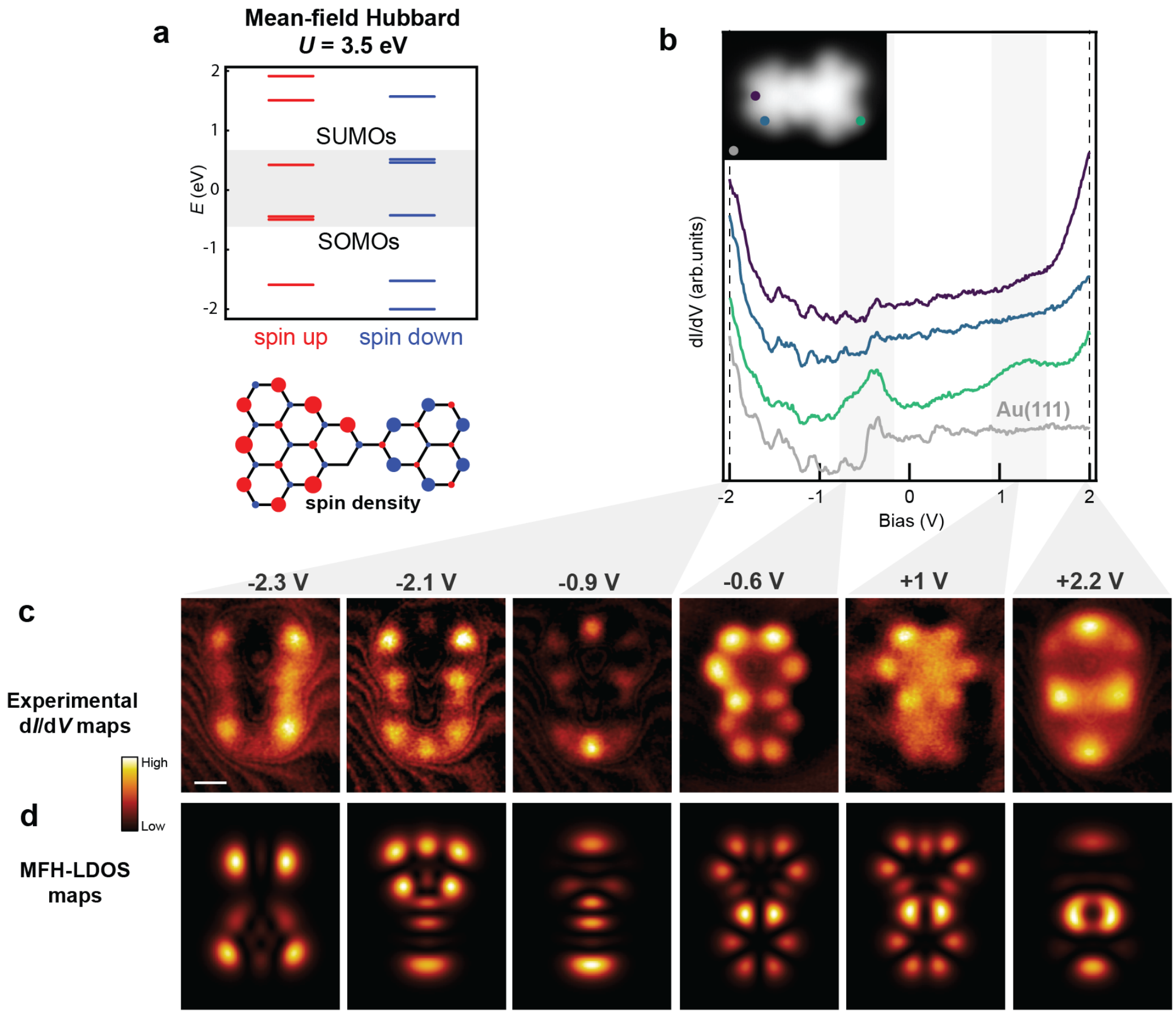

Figure S4. Electronic characterization of 2T-3T(**1**) on Au(111). (a) MFH energy spectrum (top) and spin polarization plot (bottom), where blue and red filled circles denote mean populations of spin up and spin down electrons (circles size scaling with the absolute value), respectively. Spin-carrying orbitals are highlighted by shaded gray overlays. (b) dI/dV spectroscopy on **1** revealing molecular orbital resonances (open feedback parameters: V=–2.0 V, I= 350 pA; V_m = 20 mV). Acquisition positions are indicated in the HR-STM image shown as inset (V = –0.6 V, I= 150 pA).(c) Constant-current dI/dV maps of the molecular orbital resonances, acquired with a metal tip. All the dI/dV maps were acquired with a lock-in modulation V_m = 20 mV and a current setpoint $I = 400$ pA. (d) MFH-TB LDOS of HOMO-3, HOMO-2, HOMO-1, SOMOs, SUMOs, LUMO+1, respectively.

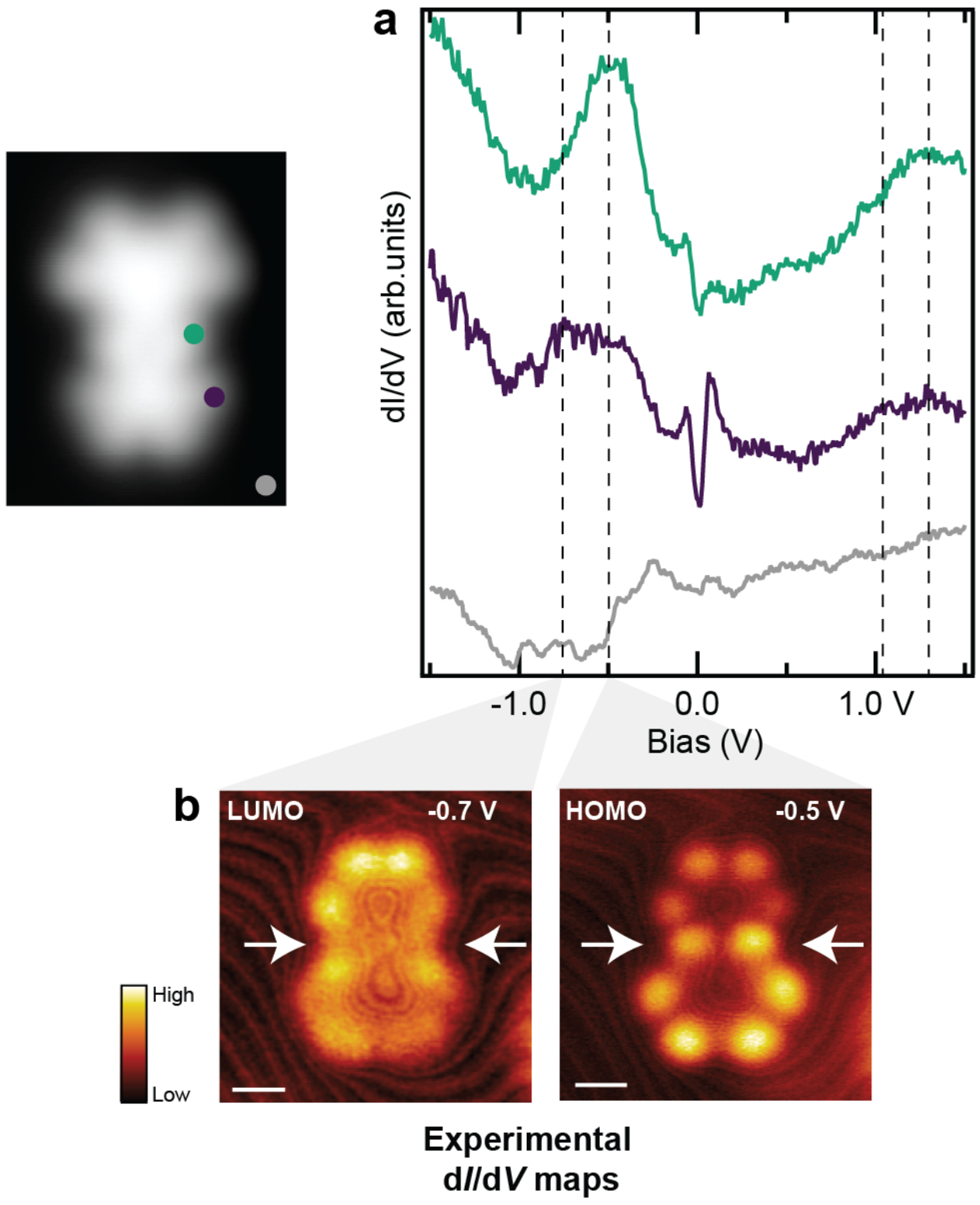

Figure S5. (a) dI/dV spectra acquired with a CO-functionalized tip on **1** adsorbed on Au(111). Vertical dotted lines indicate that both the negative- and positive-bias ion resonances associated with the frontier orbitals consist of two components. As discussed in Ref.[4] and Ref.[5], because of hybridization of singly occupied molecular orbitals (SOMOs), exchange-coupled open-shell systems possess a multireference ground state, where bonding (HOMO) and antibonding (LUMO) combinations contribute to ion resonances at the same bias polarity. The corresponding measurement positions are marked by circles in in the STM (V = –0.6 V, I= 150 pA) image on the left . Spectra were recorded with the feedback loop opened at -1.5 V, 300 pA, and V_m = 20 mV. (b) dI/dV maps recorded with a metal tip at the two-component negative ion resonance, highlighting the bonding (HOMO) and antibonding (LUMO) symmetry of the two resonant transitions. Imaging parameters: 300 pA, V_m = 20 mV. For positive biases, the broadening of the molecular orbital resonances hinders the visualization of HOMO/LUMO as distinct contributions. These observations are in line with what has been shown in Ref.[4].

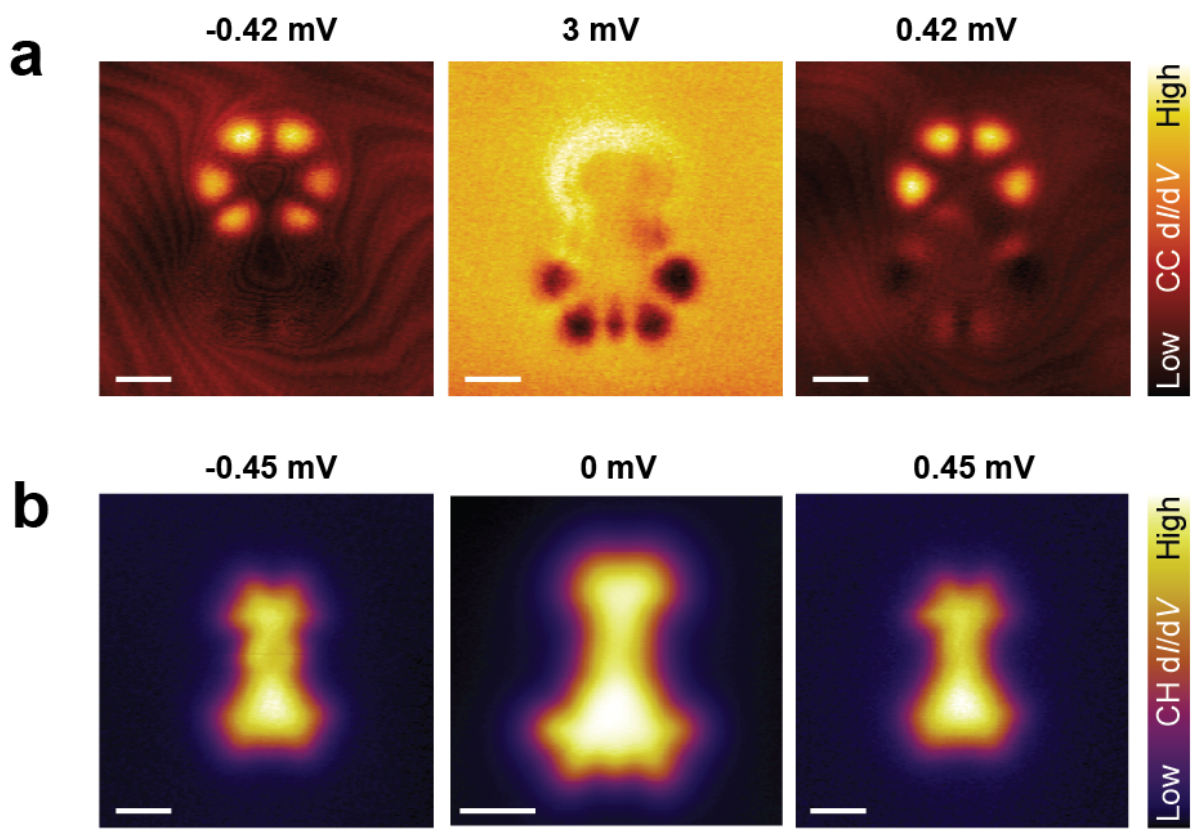

Figure S6. Constant-current and constant-height spin excitation maps of 2T-3T(**1**). (a) Constant-current dI/dV maps of the doublet-quartet spin excitation (left/right) and Kondo-like resonance (center). The noticeable difference between negative- and positive-bias spin-excitation maps reflects the many-body nature of the exchange-coupled molecular orbitals, as discussed in Ref.[4], and constitutes an indirect manifestation of its multireference ground state. Current setpoints: (a)-left/right 1 nA, (a)-center 100 pA. (b) Constant-height dI/dV maps of the doublet-quartet spin excitation (left/right) and Kondo-like resonance (center). Feedback opened on the molecule at: ±45 mV/1 nA (left/right), -15 mV/800 pA (center). The Lock-in modulation V_m used is 2 mV for all maps except the constant-height map at 0 mV, which was acquired with V_m = 4 mV. Scale bars (a,b): 0.5 nm.

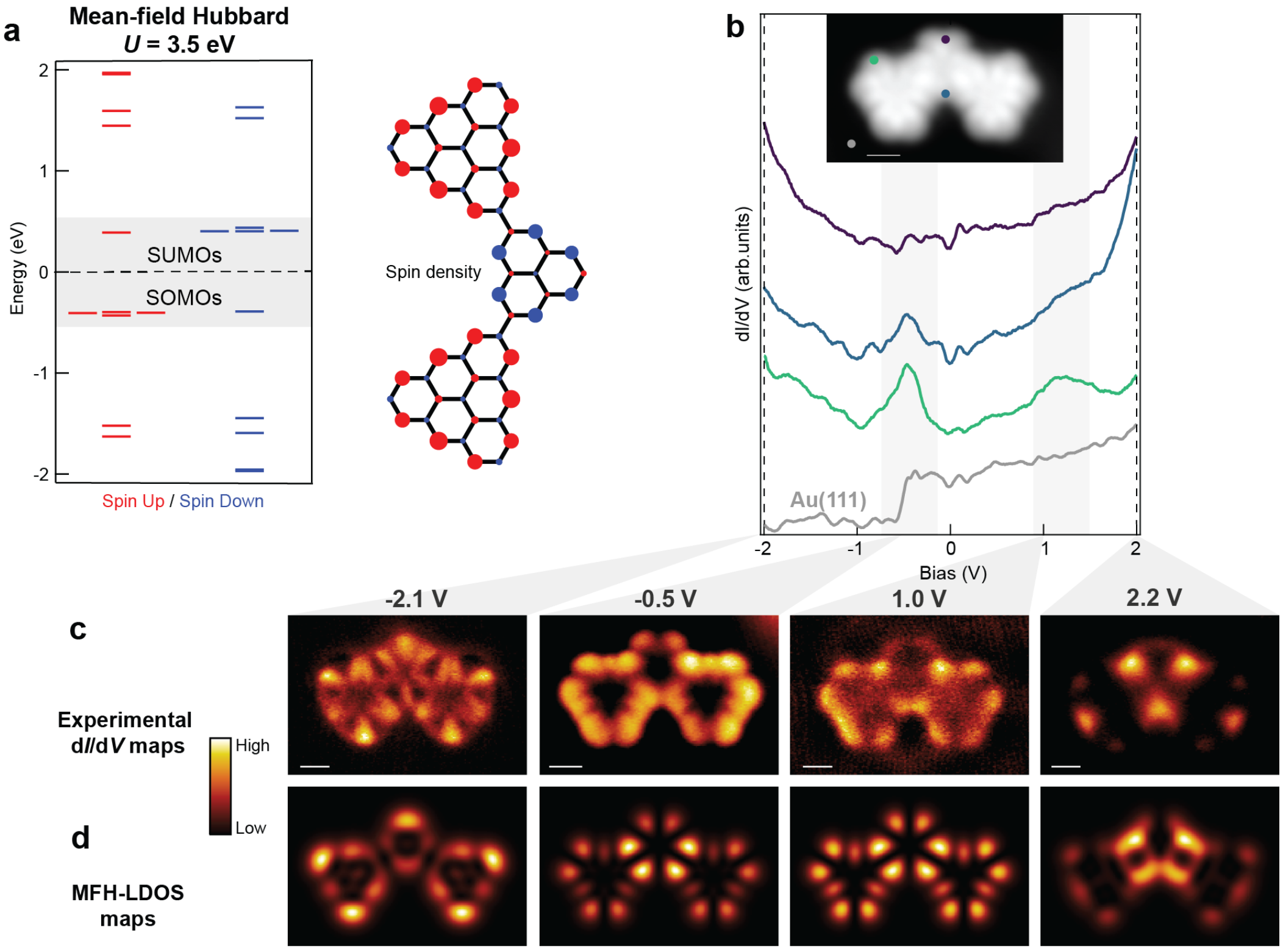

Figure S7. Electronic characterization of 3T-2T-3T(**2**) on Au(111).(a) MFH energy spectrum (left) and spin polarization plot (right), where blue and red filled circles denote mean populations of spin up and spin down electrons (circles size scaling with the absolute value), respectively. Spin-carrying orbitals are highlighted by shaded gray overlays. (b) dI/dV spectroscopy on **2** revealing molecular orbital resonances (open feedback parameters: V=–2.0 V, I= 300 pA; V_m = 30 mV). Acquisition positions are indicated in the HR-STM image shown as inset (V = –0.1 V, I= 150 pA).(c) Constant-current dI/dV maps of the molecular orbital resonances, acquired with a CO-functionalized tip. All the dI/dV maps were acquired with a lock-in modulation V_m = 30 mV and a current setpoint $I = 250$ pA. (d) MFH-TB LDOS of HOMO-1, SOMOs, SUMOs, LUMO+1, respectively.

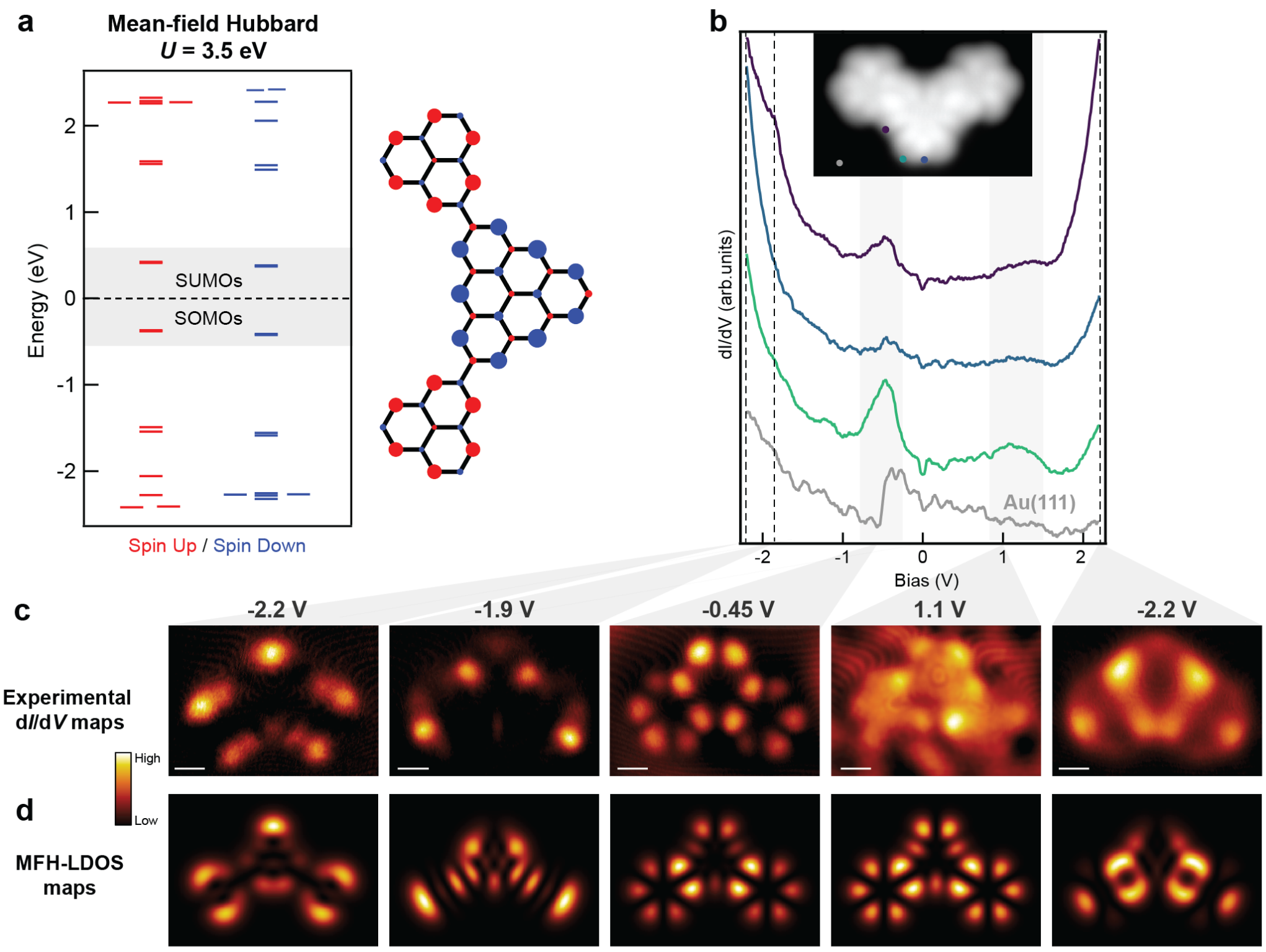

Figure S8. Electronic characterization of 2T-3T-2T (**3**) on Au(111). (a) MFH energy spectrum (left) and spin polarization plot (right), where blue and red filled circles denote mean populations of spin up and spin down electrons (circles size scaling with the absolute value), respectively. Spin-carrying orbitals are highlighted by shaded gray overlays. (b) dI/dV spectroscopy on **3** revealing molecular orbital resonances (open feedback parameters: V=–2.2 V, I= 300 pA; V_m = 30 mV). Acquisition positions are indicated in the HR-STM image shown as inset (V = –0.1 V, I= 100 pA).(c) Constant-current dI/dV maps of the molecular orbital resonances, acquired with a metal tip. All the dI/dV maps were acquired with a lock-in modulation V_m = 30 mV and a current setpoint $I = 350$ pA. (d) MFH-TB LDOS of HOMO-2, HOMO-1, SOMOs, SUMOs, LUMO+1, respectively.

References

[1] E. Turco, A. Bernhardt, N. Krane, L. Valenta, R. Fasel, M. Juríček, P. Ruffieux, *JACS Au* **2023**, *3*, 1358.

[2] C. Zhao, Q. Huang, L. Valenta, K. Eimre, L. Yang, A. V. Yakutovich, W. Xu, J. Ma, X. Feng, M. Juríček, R. Fasel, P. Ruffieux, C. A. Pignedoli, *Physical Review Letters* **2024**, *132*, 046201.

[3] M. Ternes, *New Journal of Physics* **2015**, *17*, 063016.

[4] N. Krane, E. Turco, A. Bernhardt, D. Jacob, G. Gandus, D. Passerone, M. Luisier, M. Juríček, R. Fasel, J. Fernández-Rossier, P. Ruffieux, *Nano Letters* **2023**, *23*, 9353.

[5] E. Turco, L. Tejerina, G. Catarina, A. Ortega-Guerrero, N. Krane, L. Gross, M. Juríček, S. Mishra, *Journal of the American Chemical Society* **2025**.

Details of the In-Solution Synthesis of Precursors

4.1 General Methods and Materials

Unless otherwise specified, commercially available starting materials, reagents, catalysts, and dry solvents were used without further purification. All reactions were carried out under an inert atmosphere using standard Schlenk and vacuum-line techniques. Starting materials were sourced from TCI, Sigma-Aldrich, abcr, Alfa Aesar, Acros Organics, and VWR. Catalysts were obtained from Strem Chemicals.

Column chromatography was performed using silica gel (SiO_2, particle size 0.063–0.200 mm, VWR). Thin-layer chromatography (TLC) was conducted on silica-coated aluminum sheets with a fluorescence indicator (TLC silica gel 60 F254, Merck KGaA). Further purification by recycling gel permeation chromatography (r-GPC) was conducted on a JAI HPLC LC-9110II NEXT system equipped with a fraction collector (FC-3310) and connected in series with JAIGEL-1HH, JAIGEL-2HH, and JAIGEL-2.5HH columns, using HPLC-grade chloroform as the eluent.

NMR spectra were recorded on a Bruker AV-II 300 spectrometer (300 MHz for ^{1}H, 76 MHz for ^{13}C) and a Bruker FT-NMR Avance III 400 spectrometer (400 MHz for ^{1}H and 101 MHz for ^{13}C) and Avance 600 at 298 K unless indicated otherwise. Chemical shifts (δ) are reported in parts per million (ppm) relative to the residual solvent peak (dichloromethane-*d*$_2$: $\delta(^1H)$ = 5.32 ppm, $\delta(^{13}C)$ = 53.84 ppm; chloroform-*d*: $\delta(^1H)$ = 7.26 ppm, $\delta(^{13}C)$ = 77.16 ppm). Coupling constants (*J*) are given in Hz. Peak multiplicities are denoted as follows: s = singlet, d = doublet, t = triplet, q = quartet, m = multiplet. Dichlormethane-*d*$_2$ (≥99.5 atom% D) and 1,1,2,2-tetrachloroethane-*d*$_2$ (≥99.5 atom% D) were purchased from Sigma-Aldrich.

Mass spectra were recorded with a gas chromatography mass spectrometry setup consisting of a Finnigan TraceGC Ultra and Thermo Electron Corporation Trace DSQ. High-resolution mass spectra were measured as EI (ThermoFischer Scientifics DFS), ESI, or APCI (ThermoFischer Scientifics QExactive) MS.

MALDI-TOF mass spectra were acquired on a Bruker Autoflex Speed MALDI-TOF mass spectrometer (Bruker Daltonics, Bremen, Germany). Samples for MALDI-TOF analysis were prepared by co-crystallizing the analyte with the matrix—either 1,8-dihydroxyanthracen-9(10*H*)-one (dithranol, > 98% purity, Fluka Analytical) or *trans*-2-[3-(4-*tert*-butylphenyl)-2-methyl-2-propenylidene]malononitrile (DCTB, >99% purity, Sigma-Aldrich)—in the solid state.

The key building blocks 2-bromo-10-(2,6-dimethylphenyl)anthracene (**4**),[5] 2,7-dibromo-10-(2,6-dimethylphenyl)anthracene (**12**),[5] and 2-bromo-1*H*-phenalen-1-one (**S1**)[6] were synthesized according to previously reported procedures.

4.2 Detailed Synthetic Procedures

Synthesis of 2-(4,4,5,5-tetramethyl-1,3,2-dioxaborolan-2-yl)-1*H*-phenalen-1-one (5)

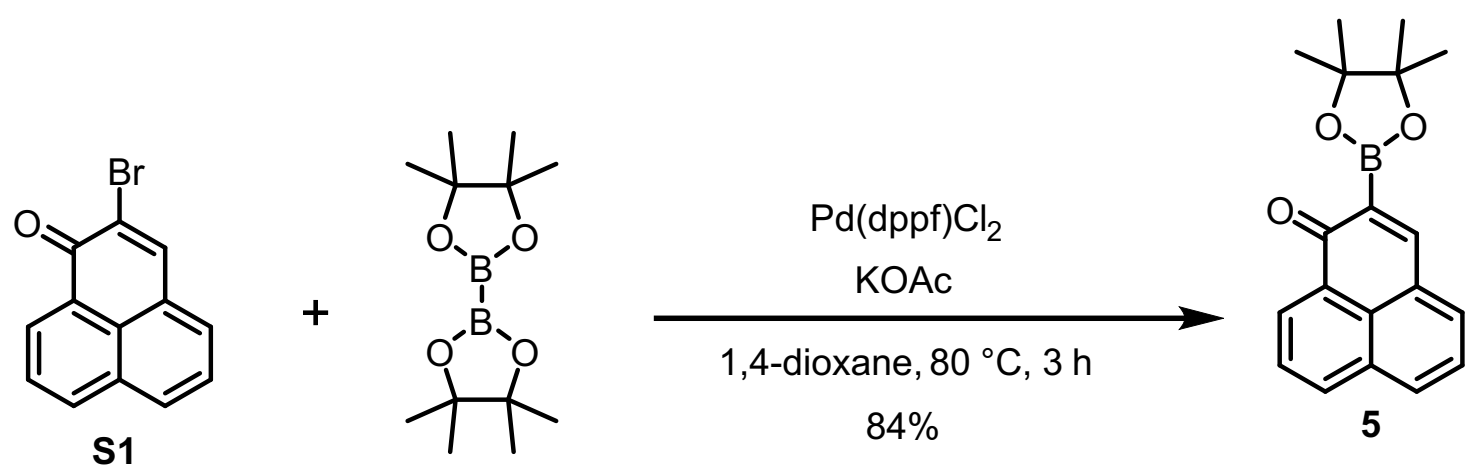

Compound **5** was prepared using a literature procedure;[7] the reaction was carried out under inert conditions; solvents were deoxygenated by bubbling with nitrogen while sonication in an ultrasonic bath for 15–30 min. In a sealed tube, bis(pinacolato)diboron (343 mg, 1.35 mmol), **S1** (350 mg, 1.35 mmol), potassium acetate (265 mg, 2.70 mmol), and Pd(dppf)Cl_2 (49 mg, 0.067 mmol, 5 mol%) were suspended in 1,4-dioxane (17 mL). The mixture was heated at 80 °C for 3 h. After cooling to room temperature, the crude mixture was diluted with water and extracted with methyl *tert*-butyl ether (MTBE). The combined organic layers were washed with water, followed by brine and dried over $MgSO_4$. After reducing the solvent under reduced pressure, the crude product was passed through a pad of silica eluding with MTBE. Evaporation of the solvent afforded the desired compound as a brown resin (0.49 g, 0.95 mmol, 84%). Purity: 70% by ^{1}H-NMR; the desired compound

could not be separated from the residual pinacolborane without decomposition. The mixture was used for cross-coupling reaction without further purification. Only a small fraction of the desired compound was purified (flash column chromatography (SiO_2, cyclohexane/ethyl acetate = 10:1 v/v) for the characterization of the compound.

HRMS (EI) *m/z:* $[M]^+$ calcd. for $C_{19}H_{19}BO_3$: 306.1422, found: 306.1430. ^{1}H NMR (400 MHz, $CDCl_3$): δ 8.62 (dd, J = 7.3, 1.2 Hz, 1H), 8.32 (s, 1H), 8.16 (dd, J = 8.1, 1.2 Hz, 1H), 8.04 (d, J = 8.6 Hz, 1H), 7.81–7.71 (m, 2H), 7.59 (dd, J = 8.3, 7.0 Hz, 1H), 1.40 (s, 12H). ^{13}C NMR (101 MHz, $CDCl_3$): δ 187.1, 152.2, 134.3, 132.6, 132.2, 132.0, 130.8, 130.0, 129.9, 128.5, 127.8, 127.5, 126.7, 84.1, 25.0.

Synthesis of 2-(10-(2,6-dimethylphenyl)anthracen-2-yl)-1*H*-phenalen-1-one (6)

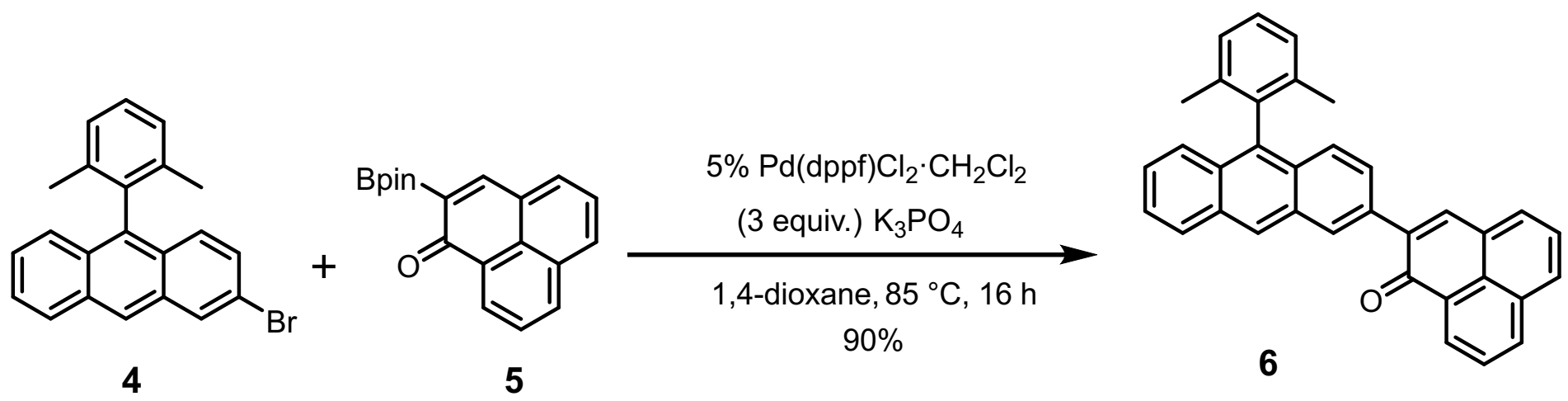

In a 25 mL Schlenk tube, compound **4** (47.2 mg, 0.131 mmol), compound **5** (20.0 mg, 65.3 µmol), Pd(dppf)Cl_2·CH_2Cl_2 (2.7 mg, 3.3 µmol), and K_3PO_4 (42 mg, 0.20 mmol) were added. The tube was evacuated and backfilled with argon three times. 1,4-Dioxane (5 mL) was then added, and the reaction mixture was stirred at 85 °C for 16 h under an argon atmosphere. After cooling to room temperature, the reaction mixture was poured into water and extracted with dichloromethane (DCM) three times. The combined organic layers were washed with brine, dried over anhydrous $MgSO_4$, and concentrated under reduced pressure. The crude product was purified by silica gel column chromatography (iso-hexane/DCM = 1:1 v/v) to afford compound **6** as an orange solid (27 mg, 90% yield).

HR-MALDI-TOF MS (matrix: DCTB) *m/z*: $[M]^+$ calcd. for $C_{35}H_{24}O$: 460.1822, found: 460.1801. ^{1}H NMR (300 MHz, dichlormethane-d_2): δ 8.72 (dd, J = 7.4, 1.2 Hz, 1H), 8.61 (s, 1H), 8.47 (d, J = 1.8 Hz, 1H), 8.29 (dd, J = 8.2, 1.2 Hz, 1H), 8.15–8.05 (m, 3H), 7.94–7.82 (m, 2H), 7.73–7.63 (m, 2H), 7.55–7.27 (m, 7H), 1.78 (s, 6H). ^{13}C NMR (76 MHz, dichlormethane-d_2): δ 184.5, 140.5, 139.4, 138.3, 138.0, 136.0, 135.3, 134.4, 132.7, 132.5, 132.2, 132.11, 132.07, 131.3, 130.6, 130.4, 129.5, 129.33, 129.28, 128.7, 128.3, 128.0, 127.9, 127.7, 127.6, 127.4, 126.5, 126.3, 125.9, 125.8, 20.3 (one carbon resonance could not be resolved because of signal overlap).

Synthesis of 10-(2,6-dimethylphenyl)-2-(1*H*-phenalen-2-yl)anthracene (7) (Mixture of isomers)

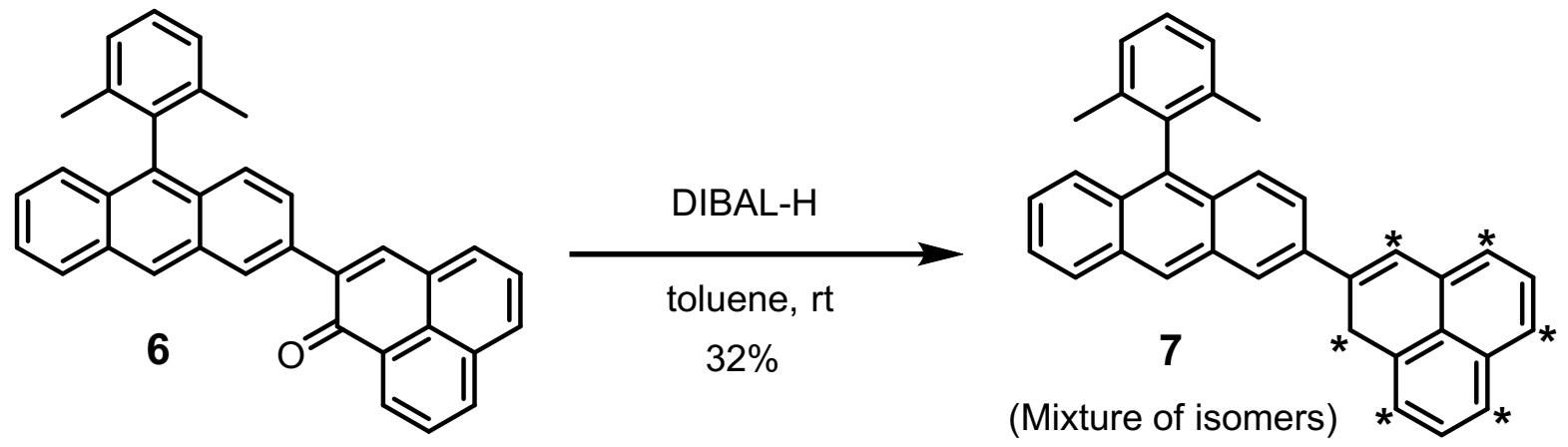

In a 10 mL Schlenk tube, compound **6** (13 mg, 28 µmol) was dissolved in deoxygenated toluene (1 mL). Diisobutylaluminum hydride (DIBAL-H, 56 uL, 1 M in hexane, 2 equiv.) was added dropwise at room temperature. Upon addition, the reaction mixture immediately changed color from orange to light yellow and exhibited sky-blue fluorescence under 365 nm UV light. The reaction was stirred at room temperature overnight. Then, the reaction mixture was passed through a plug of Florisil (6 × 2 cm) using deoxygenated toluene as the eluent. The solvent was removed under reduced pressure to yield the crude product. Thin-layer chromatography (TLC) revealed three fluorescent spots, all exhibiting sky-blue emission under UV light. Further purification by recycling gel permeation chromatography (r-GPC, fraction collected at t = 80–84 min) afforded a light-yellow solid **7** (4 mg, 32% yield) as a mixture of isomers. NMR: Compound **7** is obtained as a mixture of isomers where the methylene groups can be shifted to any α-position (asterisks) of the phenalenyl subunit. The mixture of isomers gives a complex proton

NMR spectrum with characteristic singlets for the methylene groups. The ratio between methylene groups, aromatic protons, and methyl groups is 2:18:6 matching the expected structures. High-resolution MALDI-TOF mass spectrometry (HR-MALDI-TOF MS) of the isomeric mixture showed a single dominant peak corresponding to $[M – H]^+$ at m/z = 445.1927, in excellent agreement with the calculated value (m/z = 445.1945, Δ = –4.0 ppm), and the observed isotopic pattern matched the simulated spectrum (Figures S16), confirming the molecular integrity of the compound. This isomeric mixture (**7**) was directly used as the precursor for the on-surface synthesis.

Synthesis of 2-(10-(2,6-dimethylphenyl)anthracen-2-yl)-4,4,5,5-tetramethyl-1,3,2-dioxaborolane (8)

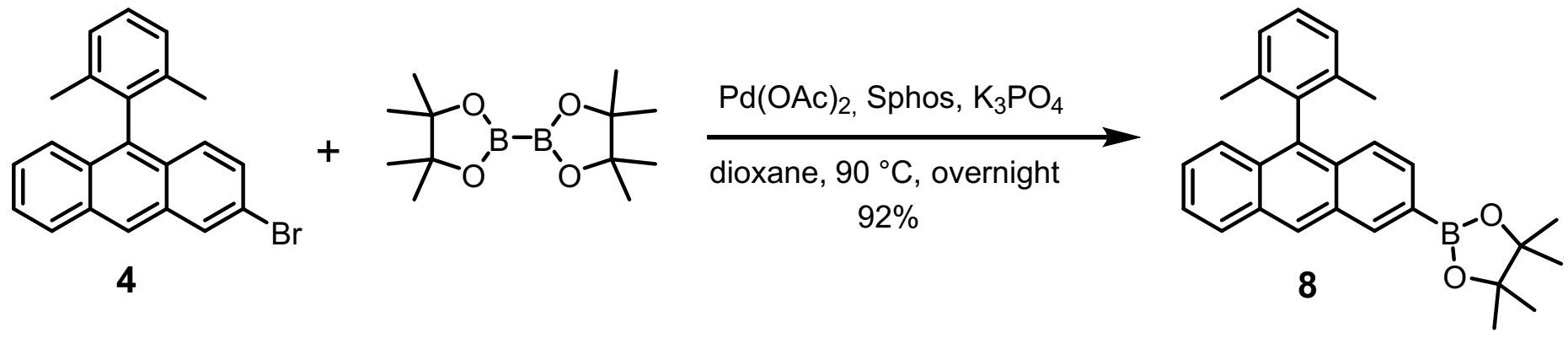

To a 50 mL Schlenk flask equipped with a magnetic stir bar, compound **4** (0.15 g, 0.40 mmol), 4,4,4′,4′,5,5,5′,5′-octamethyl-2,2′-bi(1,3,2-dioxaborolane) (0.20 g, 0.80 mmol), $Pd(OAc)_2$ (18 mg, 0.080 mmol, 10 mol%), SPhos (66 mg, 0.16 mmol), and K_3PO_4 (511 mg, 2.41 mmol) were added. The flask was evacuated and backfilled with argon three times. After further flushing with argon for 30 min, deoxygenated 1,4-dioxane (12 mL) was added. The reaction mixture was stirred at 90 °C overnight under an argon atmosphere. Upon completion, the reaction mixture was cooled to room temperature, poured into water, and extracted with ethyl acetate. The combined organic layers were dried over anhydrous $MgSO_4$, filtered, and concentrated under reduced pressure. The crude product was purified by silica gel column chromatography (iso-hexane/DCM = 1:1 v/v) to afford compound **8** as a light-yellow solid (150 mg, 92% yield).

HR-MALDI-TOF MS (matrix: dithranol) m/z: $[M]^+$ calcd. for $C_{28}H_{29}BO_2$: 408.2255, found: 408.2248. ^{1}H NMR (300 MHz, dichlormethane-d_2): δ 8.59 (d, J = 6.9 Hz, 2H), 8.14–8.08 (m, 1H), 7.62 (dd, J = 8.7, 1.2 Hz, 1H), 7.52–7.32 (m, 5H), 7.31–7.24 (m, 2H), 1.72 (s, 6H), 1.39 (s, 12H). ^{13}C NMR (76 MHz, dichlormethane-d_2): δ 138.3, 138.2, 138.0, 135.9, 132.2, 131.6, 131.1, 130.87, 130.1, 129.5, 128.3, 128.04, 127.95, 126.8, 126.2, 125.8, 125.2, 84.5, 25.3, 20.3 (one carbon resonance (presumably C–B) could not be detected).

Synthesis of 2,5-dibromo-1*H*-phenalen-1-one (9)

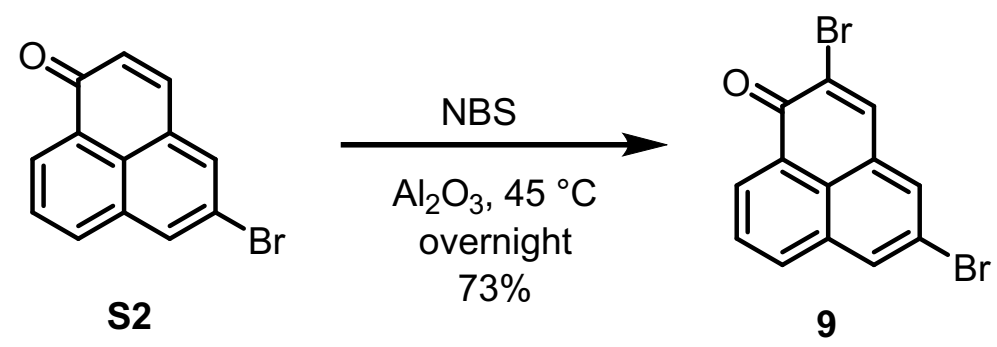

The compound was prepared using a modified literature procedure.[8] 5-Bromo-1*H*-phenalen-1-one (**S2**; 61 mg, 0.23 mmol), NBS (71 mg, 0.40 mmol), and neutral Al_2O_3 (327 mg) were ground in a mortar to obtain an evenly blended mixture which was placed in a test tube. The reaction mixture was heated at 45 °C overnight. Then, the solid mixture was extracted with CH_2Cl_2. After evaporation of the solvent, the crude product was purified by flash column chromatography (SiO_2, CH_2Cl_2) to afford the desired compound **9** as a yellow solid (58 mg, 0.17 mmol, 73%). HRMS (EI) m/z: $[M]^+$ calcd. for $C_{13}H_6Br_2O$: 335.8780, found: 335.8780. ^{1}H NMR (400 MHz, $CDCl_3$): δ 8.69 (dd, J = 7.5, 1.2 Hz, 1H), 8.21 (d, J = 1.8 Hz, 1H), 8.18–8.11 (m, 2H), 7.86–7.77 (m, 2H). ^{13}C NMR (101 MHz, $CDCl_3$): δ 178.4, 141.8, 134.7, 133.73, 133.71, 133.3, 132.5, 129.6, 128.7, 128.6, 127.4, 125.4, 120.7.

Synthesis of 2,5-bis(10-(2,6-dimethylphenyl)anthracen-2-yl)-1*H*-phenalen-1-one (10)

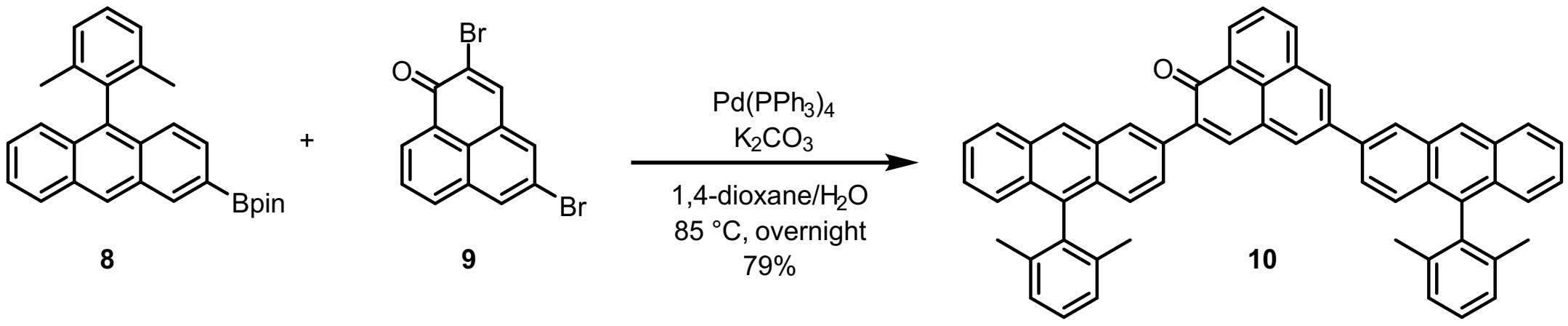

The compound was prepared using a modified literature procedure;[9] the reaction was carried out under inert conditions; solvents were deoxygenated by bubbling with nitrogen while sonication in an ultrasonic bath for 15–30 min. 2-(10-(2,6-Dimethylphenyl)anthracen-2-yl)-4,4,5,5-tetramethyl-1,3,2-dioxaborolane (**8**; 53 mg, 0.13 mmol), 2,5-dibromo-1*H*-phenalen-1-one (**9**; 22 mg, 0.060 mmol), K_2CO_3 (36 mg, 0.26 mmol), and $Pd(PPh_3)_4$ (7.4 mg, 6.0 µmol, 10 mol%) were suspended in 1,4-dioxane (7 mL) and water (1.75 mL). The mixture was heated at 85 °C overnight. After cooling to room temperature, the crude mixture was poured into water and extracted with CH_2Cl_2. The combined organic layers were washed with water, followed by brine, and dried over $MgSO_4$. After evaporation of the solvent, the crude product was purified by flash column chromatography (SiO_2, cyclohexane/ethyl acetate = 4:1 v/v) to afford the desired compound as an orange solid (37 mg, 0.050 mmol, 79%). HRMS (EI) *m/z:* $[M]^+$ calcd. for $C_{57}H_{40}O$: 740.3074, found: 740.3086. ^{1}H NMR (400 MHz, $CDCl_3$): δ 8.77 (dd, J = 7.4, 1.2 Hz, 1H), 8.62 (s, 1H), 8.58 (s, 1H), 8.51 (d, J = 1.7 Hz, 1H), 8.45 (d, J = 1.9 Hz, 1H), 8.39 (d, J = 1.6 Hz, 1H), 8.34 (dd, J = 8.2, 1.2 Hz, 1H), 8.29 (d, J = 1.7 Hz, 1H), 8.15 (s, 1H), 8.13–8.06 (m, 2H), 7.89 (dd, J = 7.7 Hz, 1H), 7.77 (dd, J = 9.1, 1.9 Hz, 1H), 7.69 (dd, J = 9.0, 1.8 Hz, 1H), 7.64 (d, J = 9.0 Hz, 1H), 7.57–7.45 (m, 5H), 7.43–7.33 (m, 4H), 7.33–7.27 (m, 4H), 1.81 (s, 6H), 1.80 (s, 6H). ^{13}C NMR (101 MHz, $CDCl_3$): δ 184.5, 140.0, 139.9, 139.6, 138.0, 137.9, 137.6, 137.4, 136.3, 136.0, 135.6, 135.1, 133.6, 132.7, 132.4, 132.0, 131.8, 131.7, 131.2, 131.0, 130.2, 130.1, 129.28, 129.26, 129.2, 129.0, 128.92, 128.85, 128.01, 127.99, 127.9, 127.7, 127.6, 127.3, 127.1, 127.04, 126.96, 126.6, 126.2, 126.12, 126.05, 126.0, 125.8, 125.42, 125.35, 20.29, 20.27 (four carbon resonances could not be resolved because of signal overlap).

Synthesis of 3,3'-(1*H*-phenalene-2,5-diyl)bis(9-(2,6-dimethylphenyl)anthracene) (11) (Mixture of isomers)

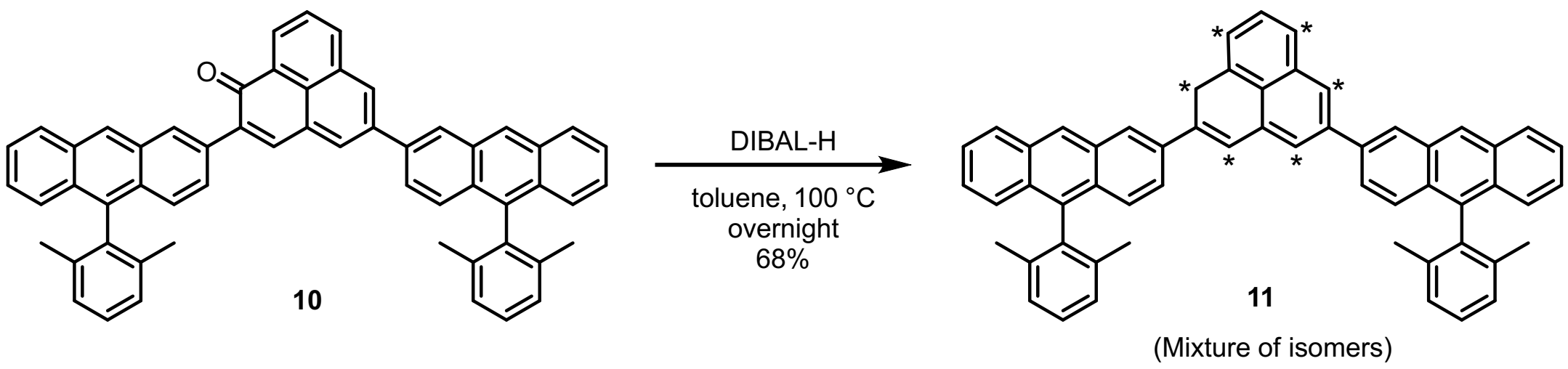

The compound was prepared using a modified literature procedure;[10] the reaction was carried out under inert conditions; solvents were deoxygenated by freeze-pump-thaw technique in three cycles under a nitrogen atmosphere. 2,5-Bis(10-(2,6-dimethylphenyl)anthracen-2-yl)-1*H*-phenalen-1-one (**10**; 9.3 mg, 0.020 mmol) was suspended in toluene (1 mL). A solution of DIBAL-H (25 µL, 0.030 mmol, 1.0 M in toluene) was added dropwise at room temperature and the reaction mixture was heated at 100 °C overnight. After cooling to room temperature, the reaction mixture was passed through a pad of Florisil® with toluene as eluent under the exclusion of air. After removal of the solvent in vacuum, the desired compound **11** was obtained as an orange solid (6.2 mg, 0.010 mmol, 68%). NMR: Compound **11** is obtained as a mixture of isomers where the methylene groups can be shifted to any α-position (asterisks) of the phenalenyl subunit. The mixture of isomers gives a complex proton NMR spectrum with characteristic singlets for the methylene groups. The ratio between methylene groups, aromatic protons, and methyl groups is 2:28:12 matching the expected structures. HRMS (EI) *m/z:* $[M]^+$ calcd. for $C_{57}H_{42}$: 726.3281, found: 726.3271.

Synthesis of 2,2'-(10-(2,6-dimethylphenyl)anthracene-2,7-diyl)bis(1*H*-phenalen-1-one) (13)

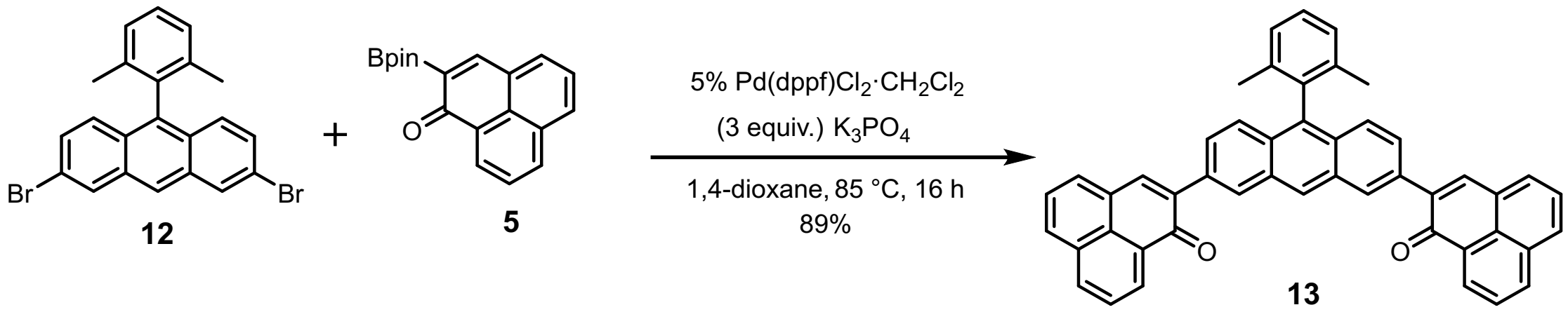

In a 25 mL Schlenk tube, compound **12** (9.5 mg, 22 μmol), compound **5** (20.0 mg, 65.3 μmol), Pd(dppf)Cl_2·CH_2Cl_2 (5.3 mg, 6.5 μmol), and K_3PO_4 (83 mg, 0.39 mmol) were added. The tube was evacuated and backfilled with argon three times. 1,4-Dioxane (5 mL) was then added, and the reaction mixture was stirred at 85 °C for 16 h under an argon atmosphere. After cooling to room temperature, the mixture was poured into water and extracted with dichloromethane (DCM) three times. The combined organic layers were washed with brine, dried over anhydrous $MgSO_4$, and concentrated under reduced pressure. The crude product was purified by silica gel column chromatography (iso-hexane/ethyl acetate = 3:1 v/v) to afford compound **13** as an orange solid (12 mg, 89% yield).

HR-MALDI-TOF MS (matrix: DCTB) *m*/*z*: $[M]^+$ calcd. for $C_{48}H_{30}O_2$: 638.2240, found: 638.2258. ^{1}H NMR (300 MHz, 1,1,2,2-tetrachloroethane-d_2): δ 8.69 (dd, J = 7.4, 1.2 Hz, 2H), 8.61 (s, 1H), 8.41 (d, J = 1.7 Hz, 2H), 8.24 (dd, J = 8.2, 1.2 Hz, 2H), 8.08–7.99 (m, 4H), 7.90–7.78 (m, 4H), 7.67–7.57 (m, 4H), 7.49 (d, J = 9.0 Hz, 2H), 7.36 (dd, J = 8.5, 6.3 Hz, 1H), 7.27 (d, J = 7.5 Hz, 2H), 1.81 (s, 6H). ^{13}C NMR (76 MHz, 1,1,2,2-tetrachloroethane-d_2): δ 184.3, 140.3, 138.9, 137.9, 137.3, 135.4, 135.2, 133.5, 132.0, 131.8, 131.7, 131.1, 129.8, 129.3, 129.0, 128.0, 127.8, 127.6, 127.5, 127.3, 127.2, 127.0, 125.7, 74.2, 20.3 (one carbon resonance could not be resolved because of signal overlap).

Synthesis of 10-(2,6-dimethylphenyl)-2,7-di(1*H*-phenalen-2-yl)anthracene (14) (Mixture of isomers)

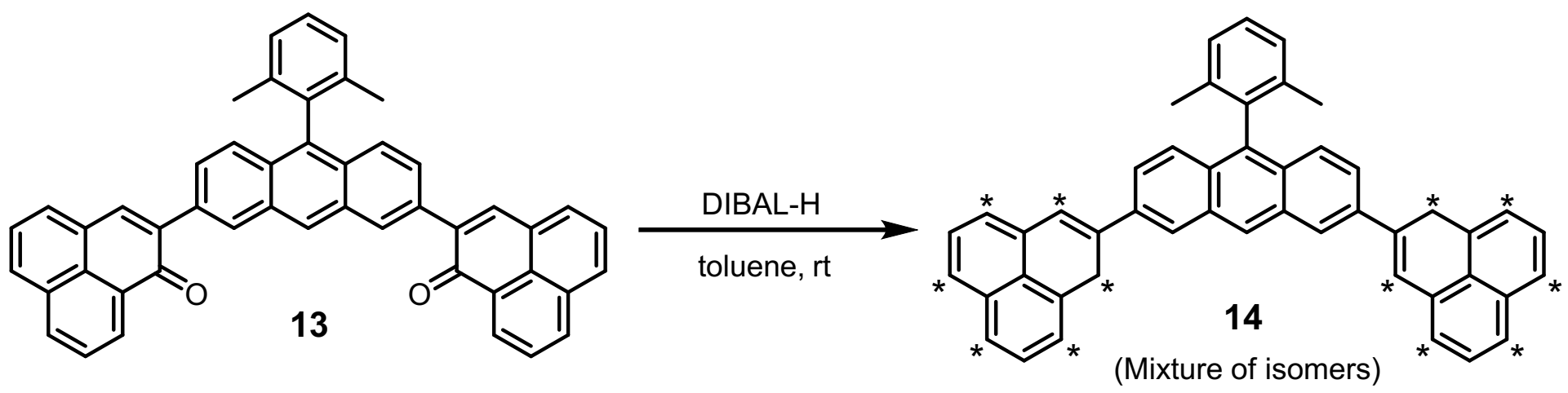

Method A: In a 10 mL Schlenk tube connected to a vacuum trap (Figure S32), compound **13** (5.0 mg, 7.8 μmol) was dissolved in dry, deoxygenated toluene (3 mL). Diisobutylaluminum hydride (DIBAL-H; 62.6 μL, 62.6 μmol, 1 M in hexane) was added dropwise at room temperature. Upon addition, the reaction mixture immediately changed color from orange to light yellow and exhibited sky-blue fluorescence under 365 nm UV light. The mixture was stirred at room temperature overnight. After completion, the reaction was carefully quenched with methanol and the solvent was removed *in situ* under reduced pressure. The residue was transferred into a glovebox, washed with dichloromethane (DCM), and filtered through a syringe filter. The DCM filtrate was then evaporated under reduced pressure to afford the crude isomeric mixture of compound **14**. HR-MALDI-TOF MS (matrix: dithranol) *m*/*z*: $[M]^+$ calcd. for $C_{48}H_{34}$: 610.2655, found: 610.2185. High-resolution MALDI-TOF mass spectrometry of the crude product showed an additional peak, likely resulting from partial oxidation of compound **14** during measurement (Figure S32). The resulting mixture was used directly as the precursor for the on-surface synthesis.

Alternative synthetic procedure for compound 14 (Mixture of isomers)

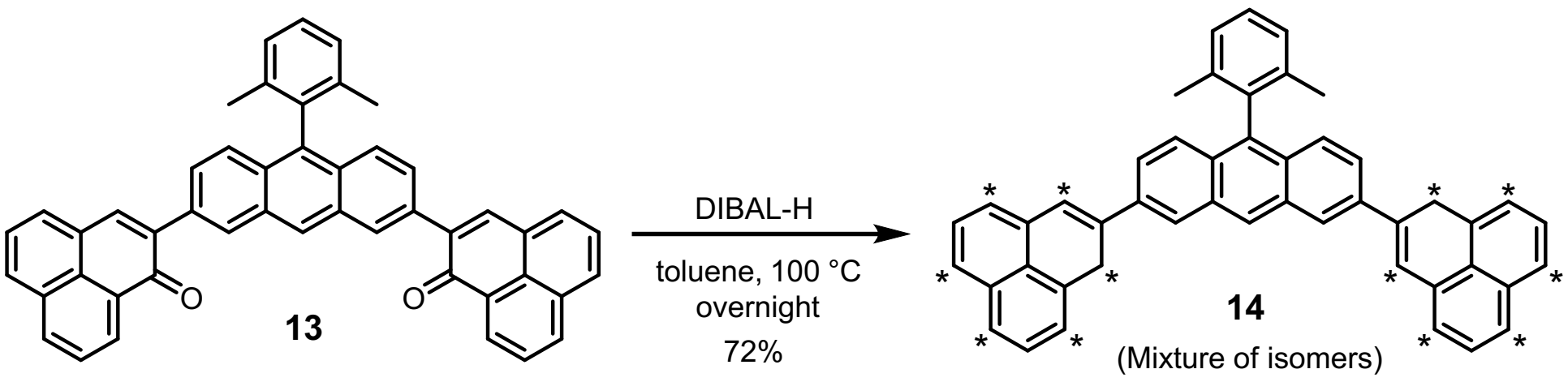

Method B: The compound was prepared using a modified literature procedure;[10] the reaction was carried out under inert conditions; solvents were deoxygenated by freeze-pump-thaw technique in three cycles under a nitrogen atmosphere. 2,2'-(10-(2,6-Dimethylphenyl)anthracene-2,7-diyl)bis(1*H*-phenalen-1-one) (**13**; 7.0 mg, 0.010 mmol) was suspended in toluene (1 mL). A solution of DIBAL-H (42 µL, 0.040 mmol, 1.0 M in toluene) was added dropwise at room temperature and the reaction mixture was heated at 100 °C overnight. After cooling to room temperature, the reaction mixture was passed through a pad of Florisil® with toluene as an eluent under the exclusion of air. After removal of the solvent in vacuum, the desired compound **14** was obtained as yellow solid (4.6 mg, 0.010 mmol, 72%). NMR: Compound **14** is obtained as a mixture of isomers where the methylene groups can be shifted to any α-position (asterisks) of the phenalenyl subunit. The mixture of isomers gives a complex proton NMR spectrum with characteristic singlets for the methylene groups. The ratio between methylene groups, aromatic protons, and methyl groups is 4:24:6 matching the expected structures. HRMS (EI) *m*/*z:* [*M*]+ calcd. for $C_{48}H_{34}$: 610.2655, found: 610.2641.

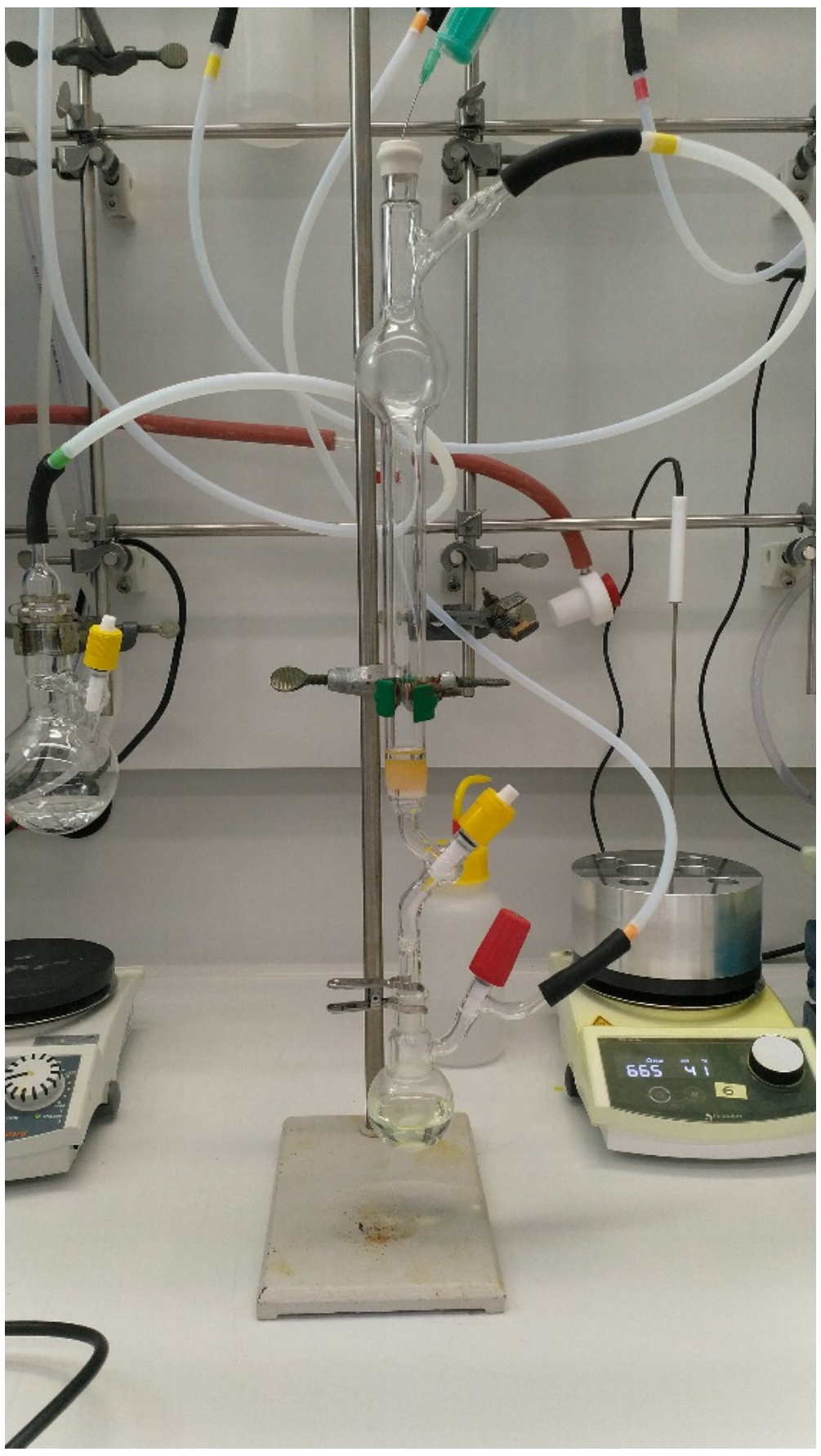

Figure S9. Custom-made glassware for inert column chromatography.

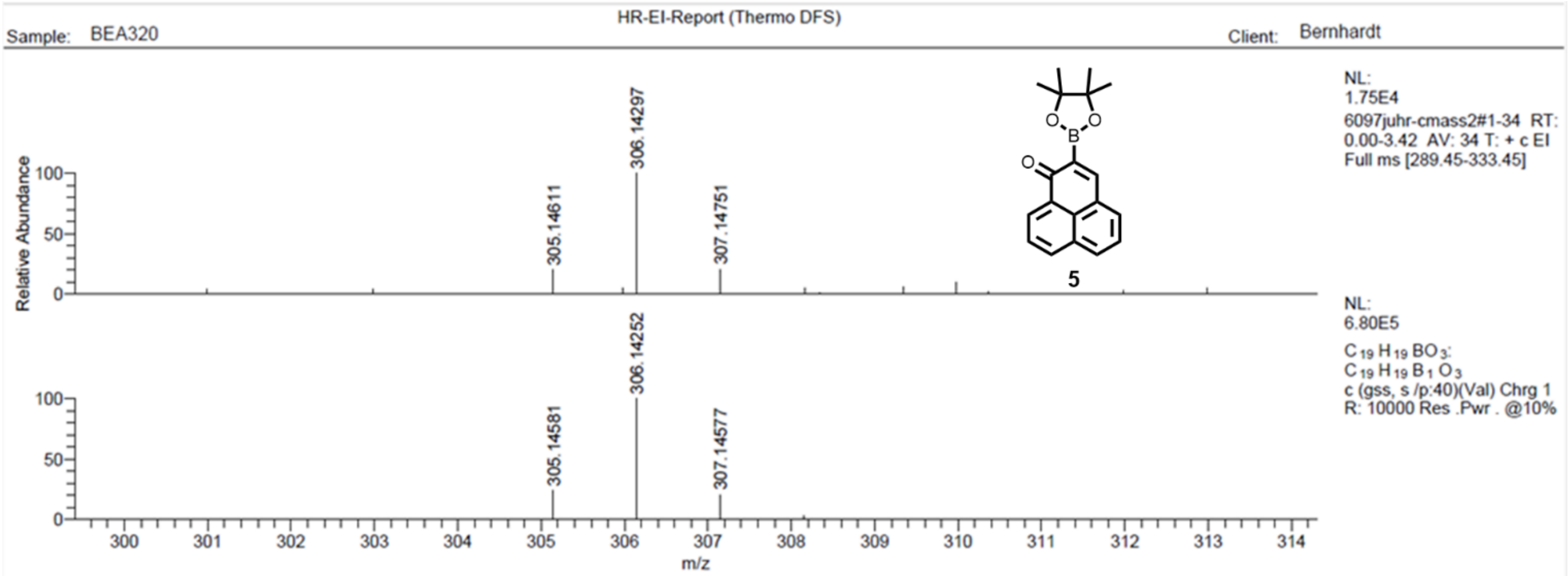

6097juhr-cmass2#1-34 RT: 0.00-3.42 AV: 34
T: + c EI Full ms [289.45-333.45]
m/z= 305.96759-306.34339

m/z	Intensity	Relative	Theo. Mass	Delta (ppm)	Composition
306.14297	17515.5	100.00	306.14218	2.60	$C_{19}H_{19}O_3B$

Figure S10. HR-EI-MS report of compound **5**.

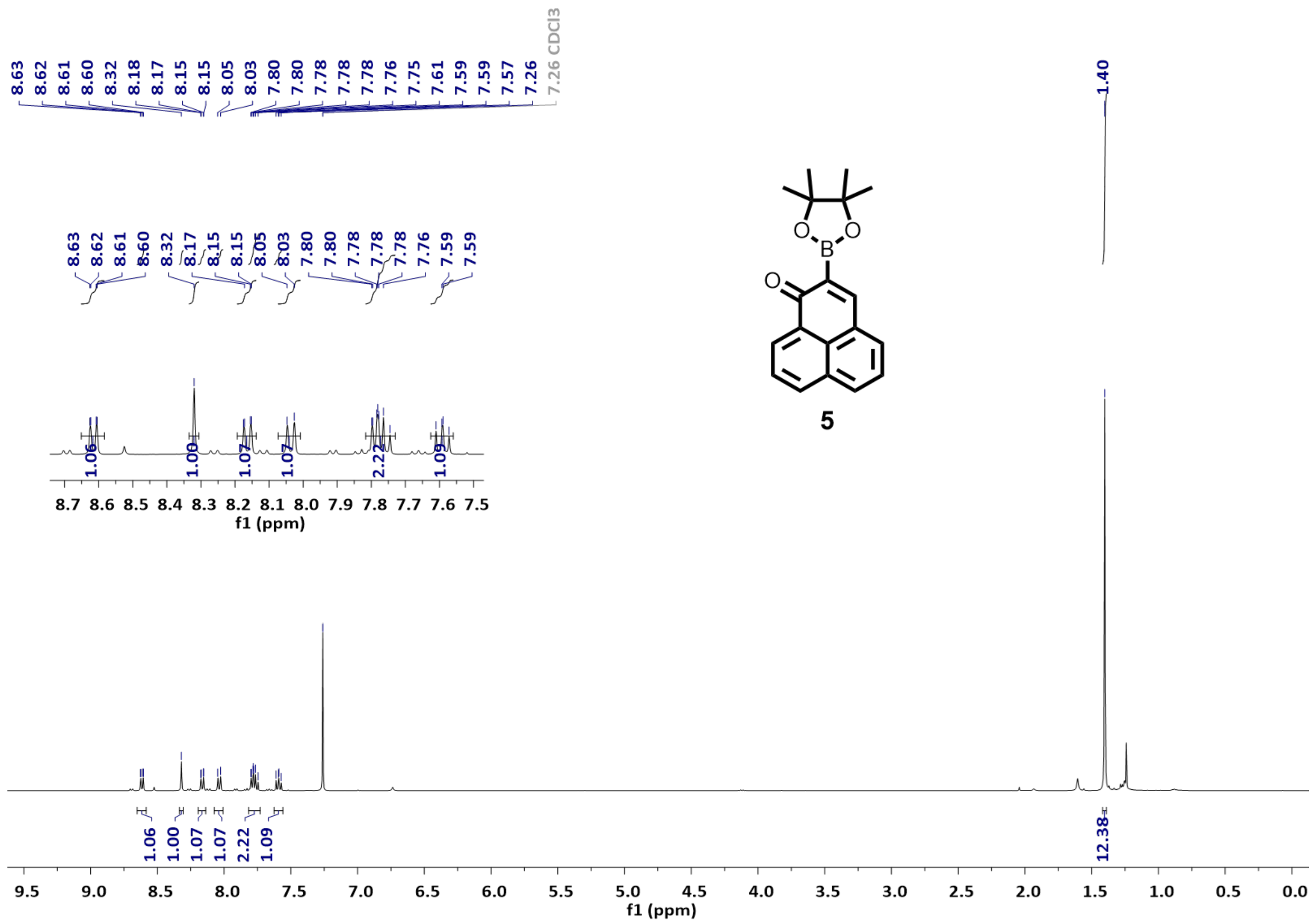

Figure S11. ^{1}H-NMR spectrum of **5** dissolved in $CDCl_3$, 400 MHz, 298 K.

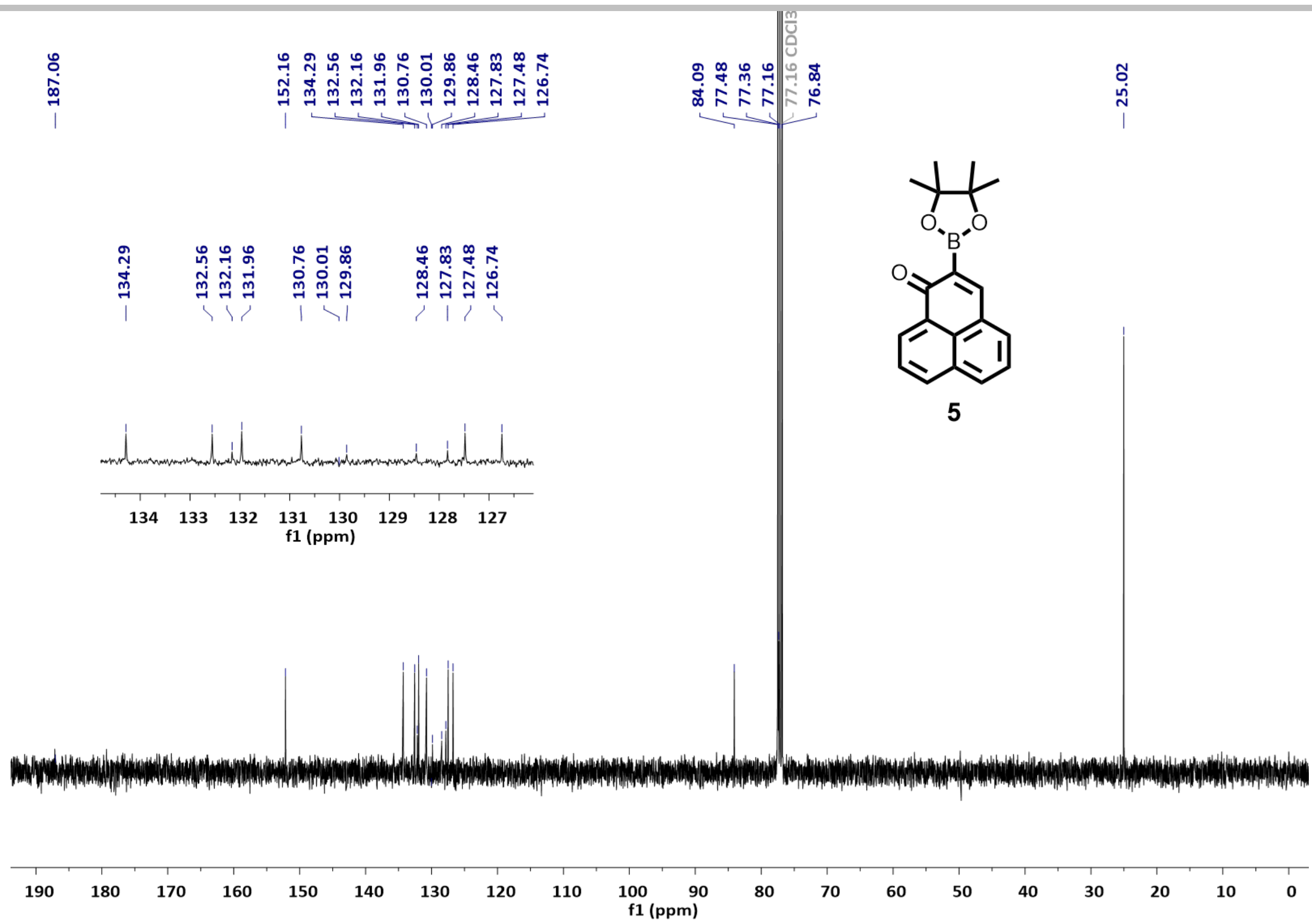

Figure S12. ^{13}C-NMR spectrum of **5** dissolved in $CDCl_3$, 101 MHz, 298 K.

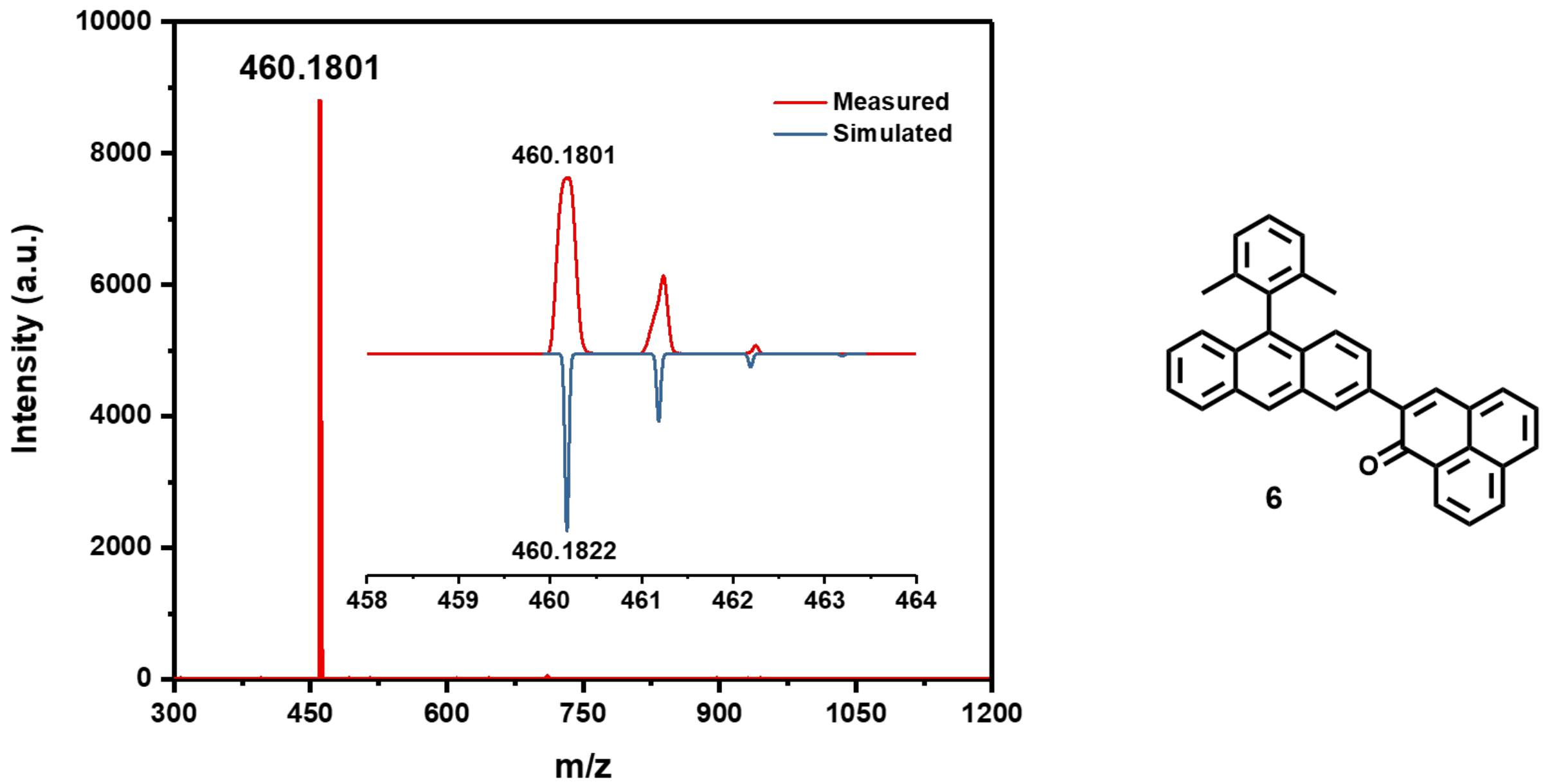

Figure S13. Liquid-state HR-MALDI-TOF-MS of **6** (matrix: DCTB).

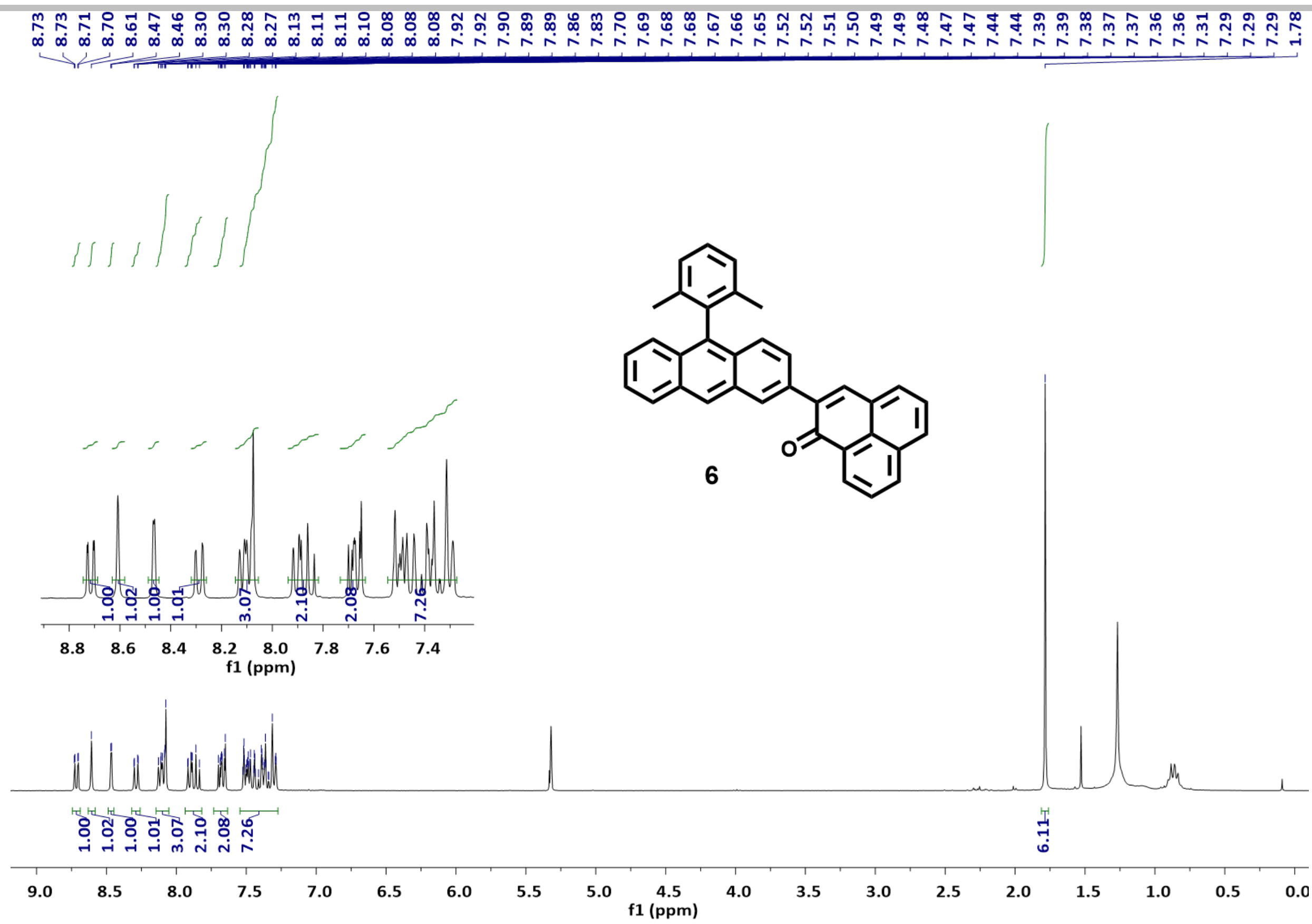

Figure S14. ^{1}H-NMR spectrum of **6** dissolved in CD_2Cl_2, 300 MHz, 296 K.

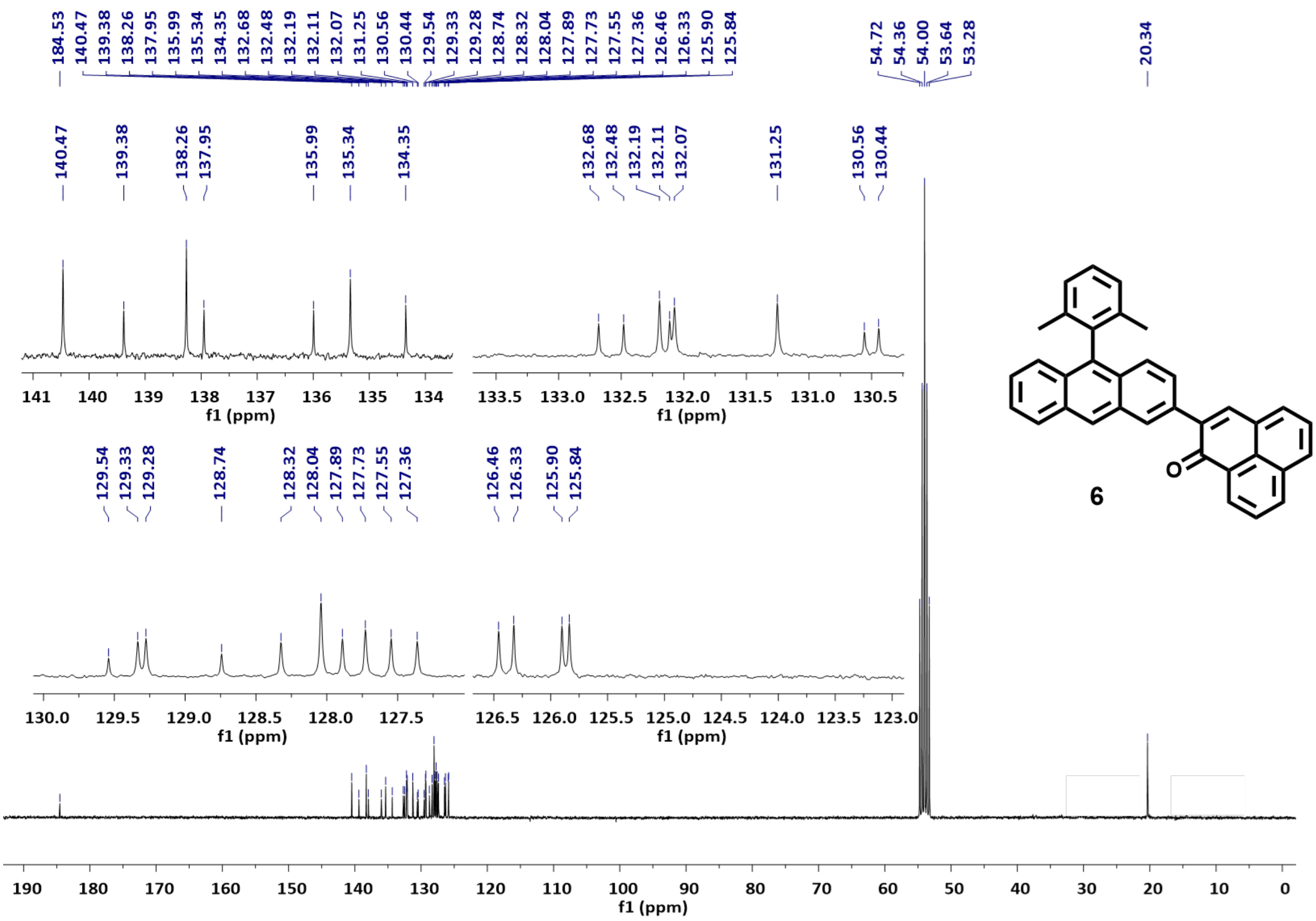

Figure S15. ^{13}C-NMR spectrum of **6** dissolved in CD_2Cl_2, 76 MHz, 296 K (one carbon resonance could not be resolved because of signal overlap).

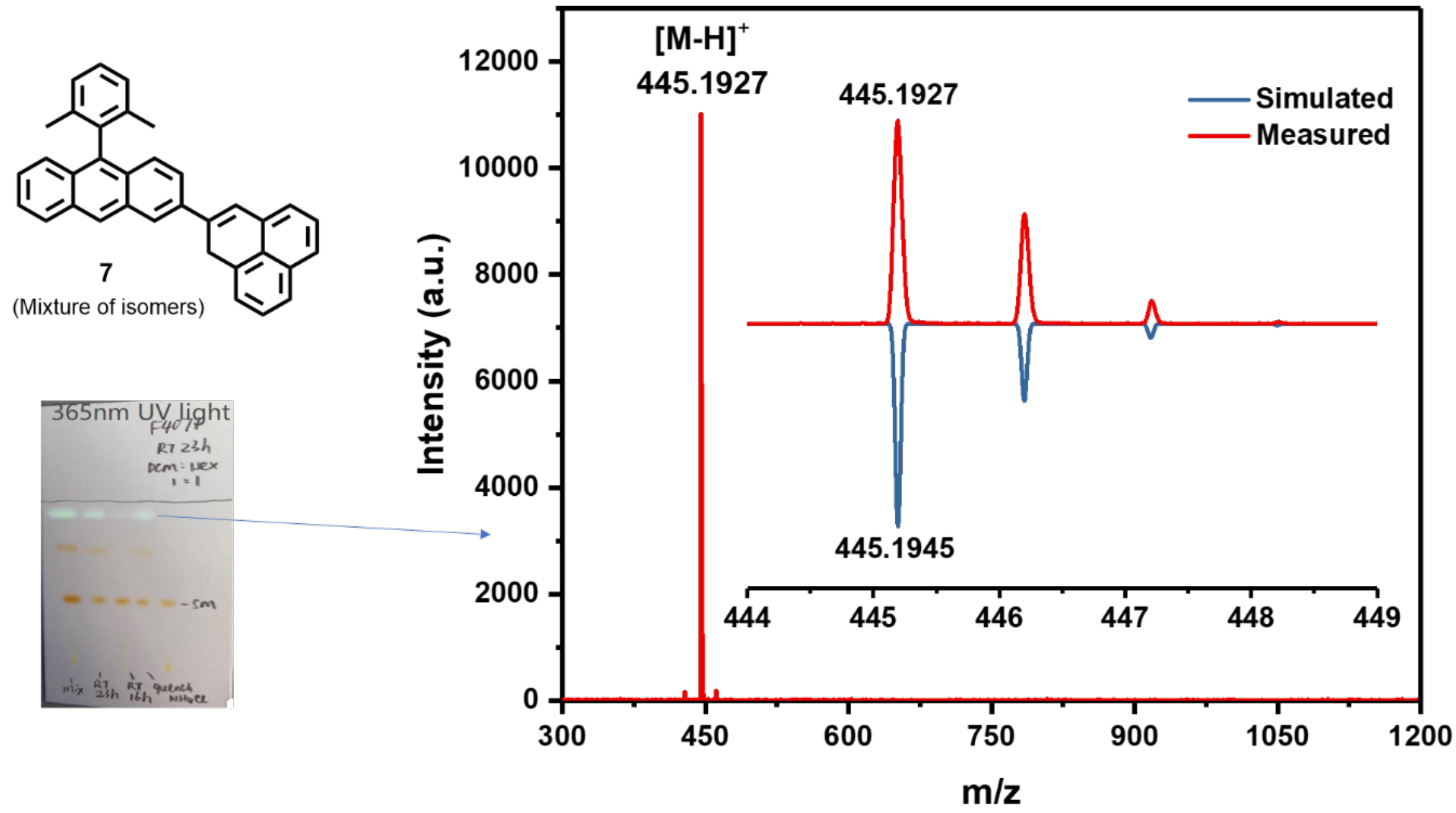

Figure S16. Compound **7** exhibited a distinct spot on TLC, showing sky-blue fluorescence under 365 nm UV light. And the liquid-state high-resolution MALDI-TOF mass spectrum of compound **7** (matrix: dithranol).

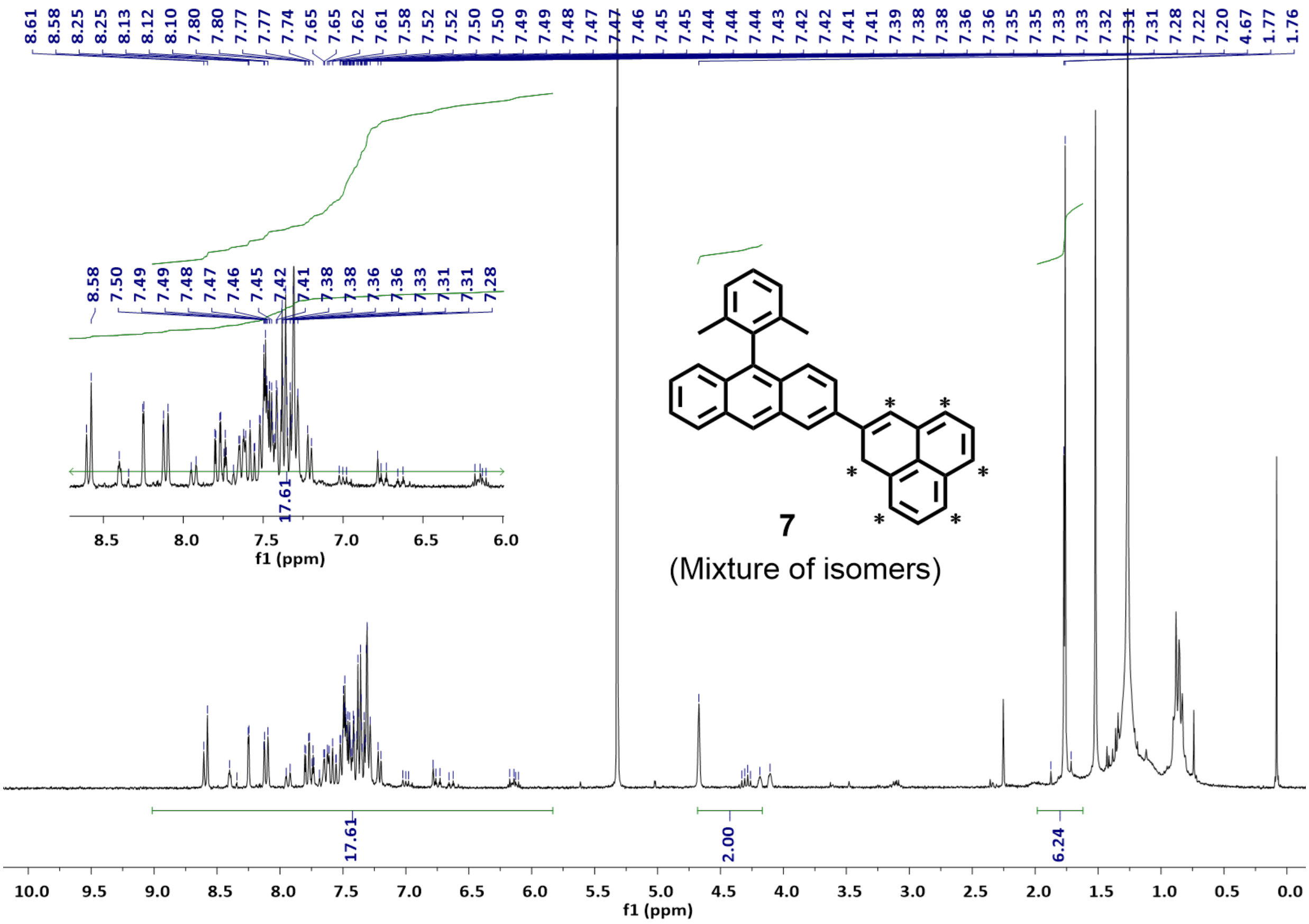

Figure S17. ^{1}H-NMR spectrum of **7** dissolved in CD_2Cl_2, 300 MHz, 296 K.

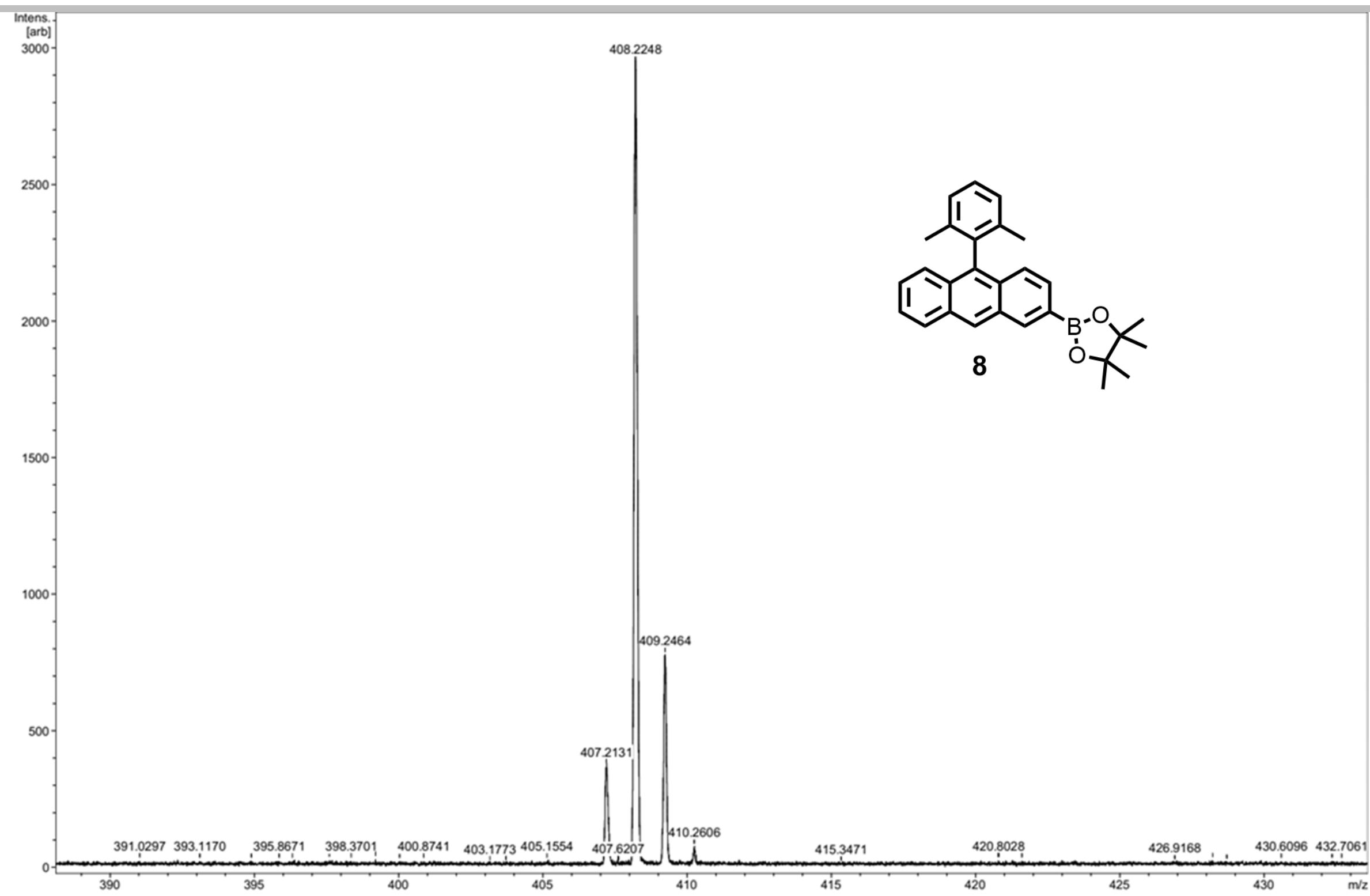

Bruker Daltonics flexControl

Display Screenshot - Generated On 2022-01-09 14h32m55s

Figure S18. Liquid-state HR-MALDI-TOF-MS of **8** (matrix: dithranol).

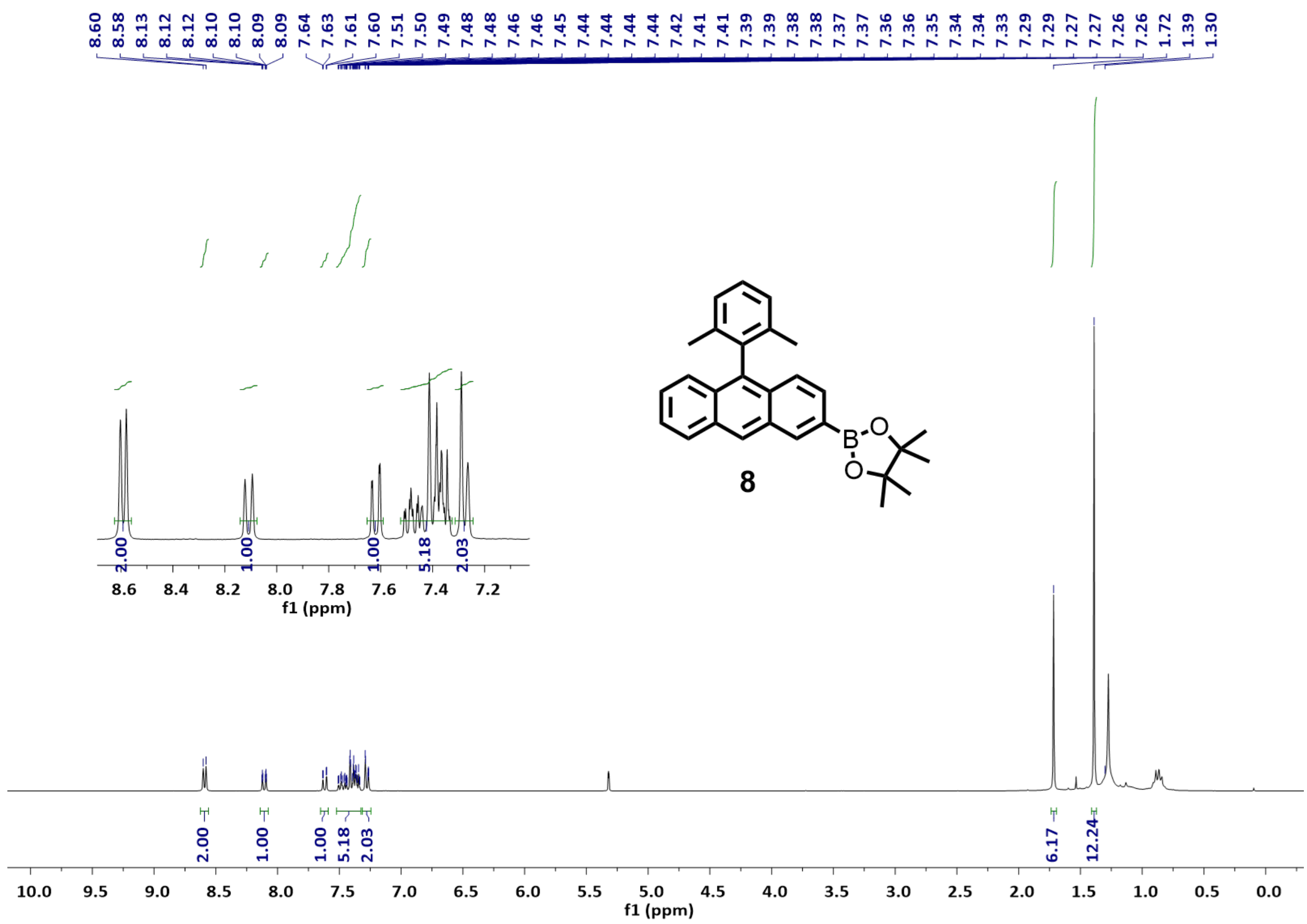

Figure S19. ^{1}H-NMR spectrum of **8** dissolved in CD_2Cl_2, 300 MHz, 296 K.

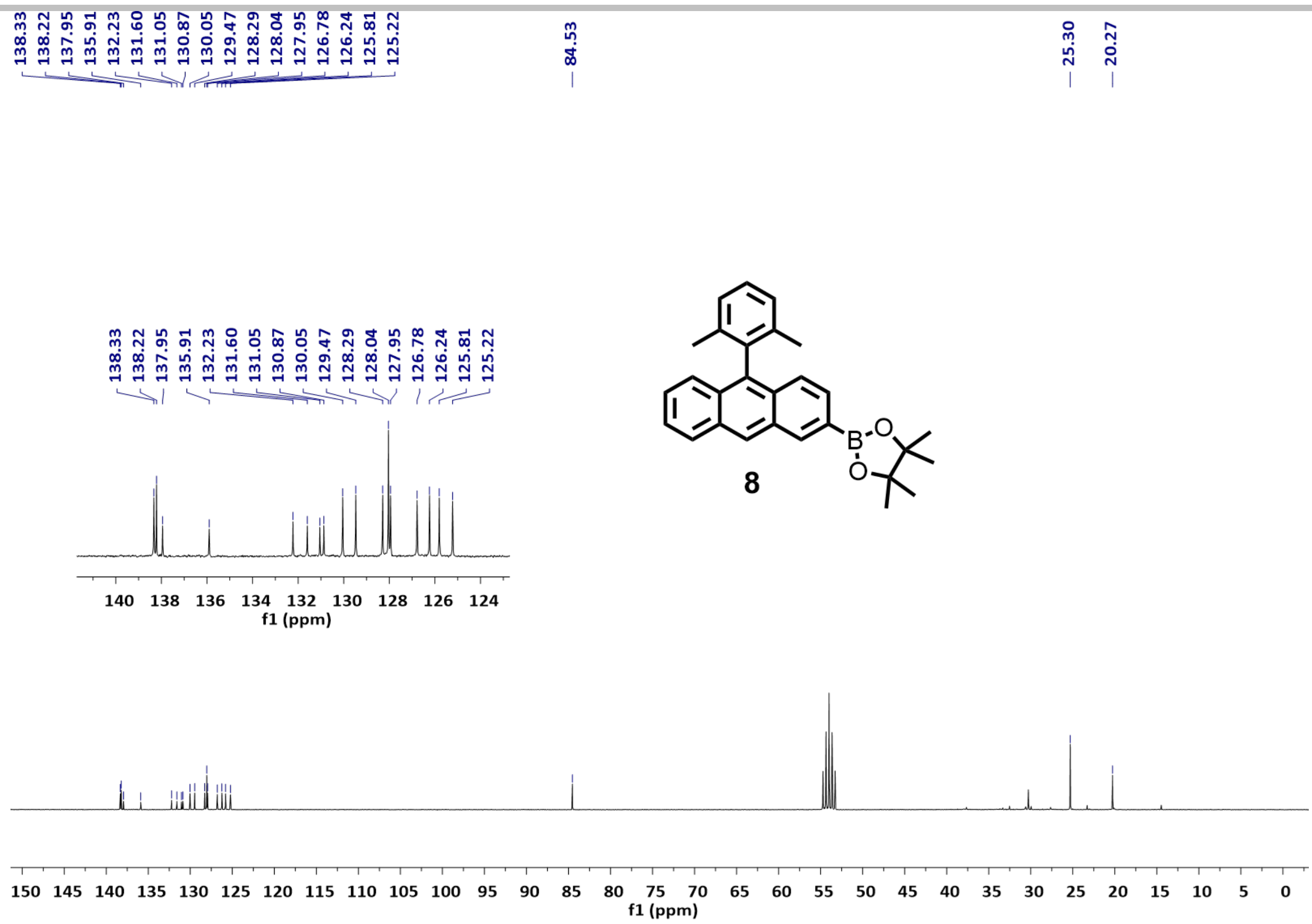

Figure S20. ^{13}C-NMR spectrum of **8** dissolved in CD_2Cl_2, 76 MHz, 296 K (one carbon resonance (presumably C–B) could not be detected).

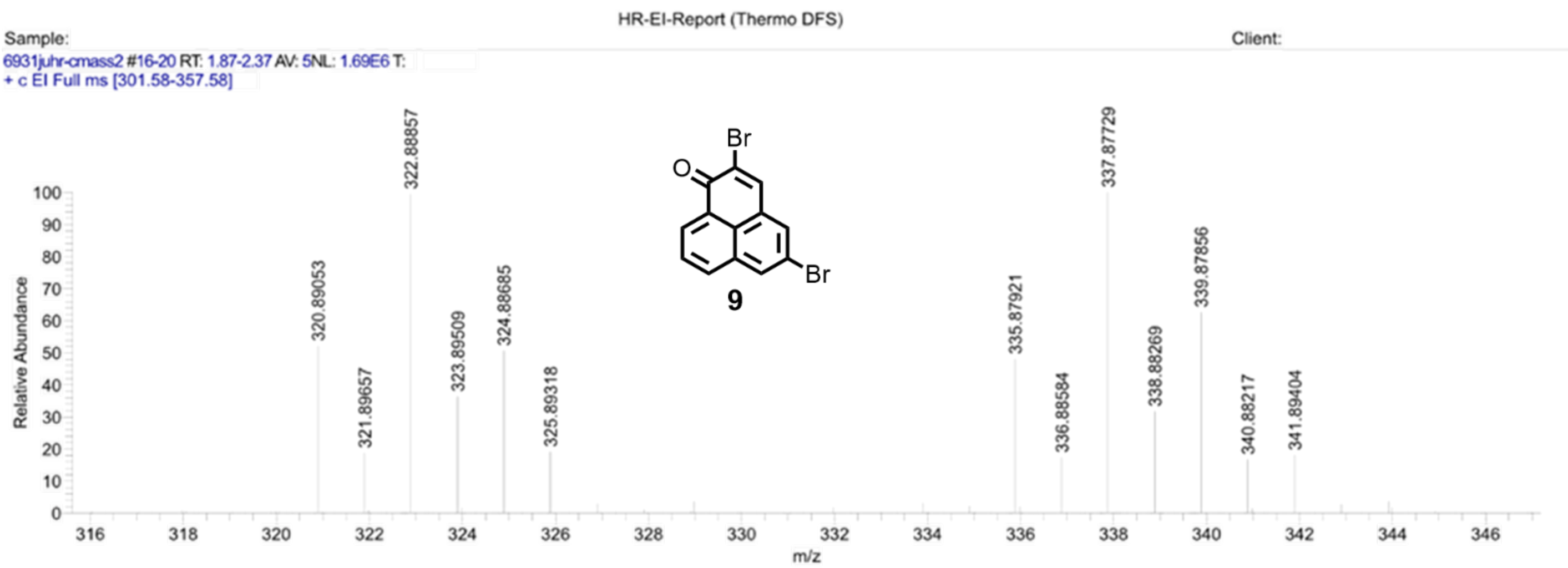

6931juhr-cmass2 #16-20 RT: 1.87-2.37 AV: 5 T: +
c EI Full ms [301.58-357.58]
m/z= 320.07437-336.16524

m/z	Intensity	Relative	Theo. Mass	Delta (ppm)	Composition
320.89053	882529.0	52.42	320.89090	-1.16	$C_{13}H_7Br_2$
324.88685	857002.4	50.91	324.88582	3.19	$C_{12}H_7OBr_2$
335.87921	812783.6	48.28	335.87799	3.63	$C_{13}H_6OBr_2$

Figure S21. HR-EI-MS report of compound **9**.

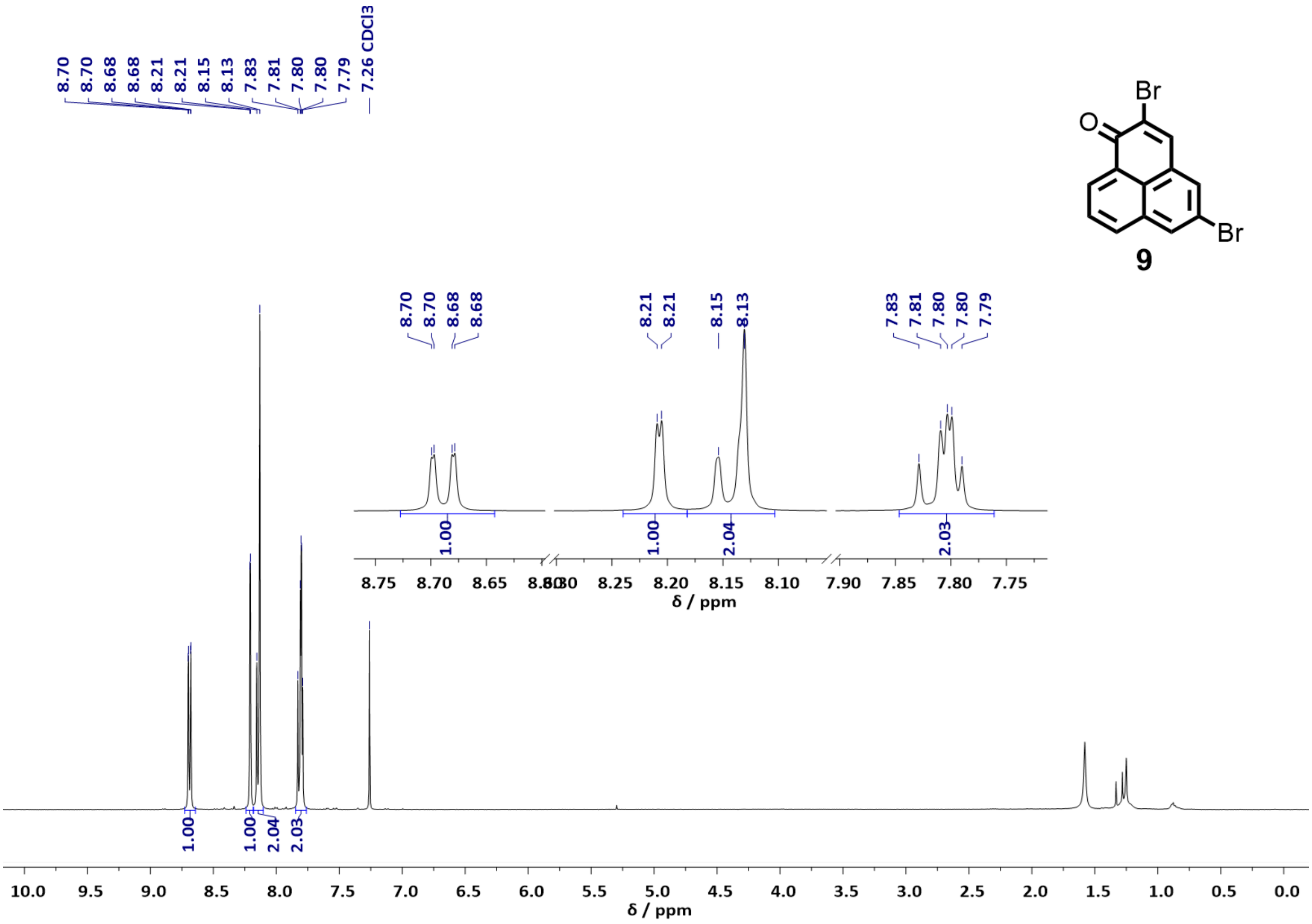

Figure S22. ^{1}H-NMR spectrum of **9** dissolved in $CDCl_3$, 400 MHz, 298 K.

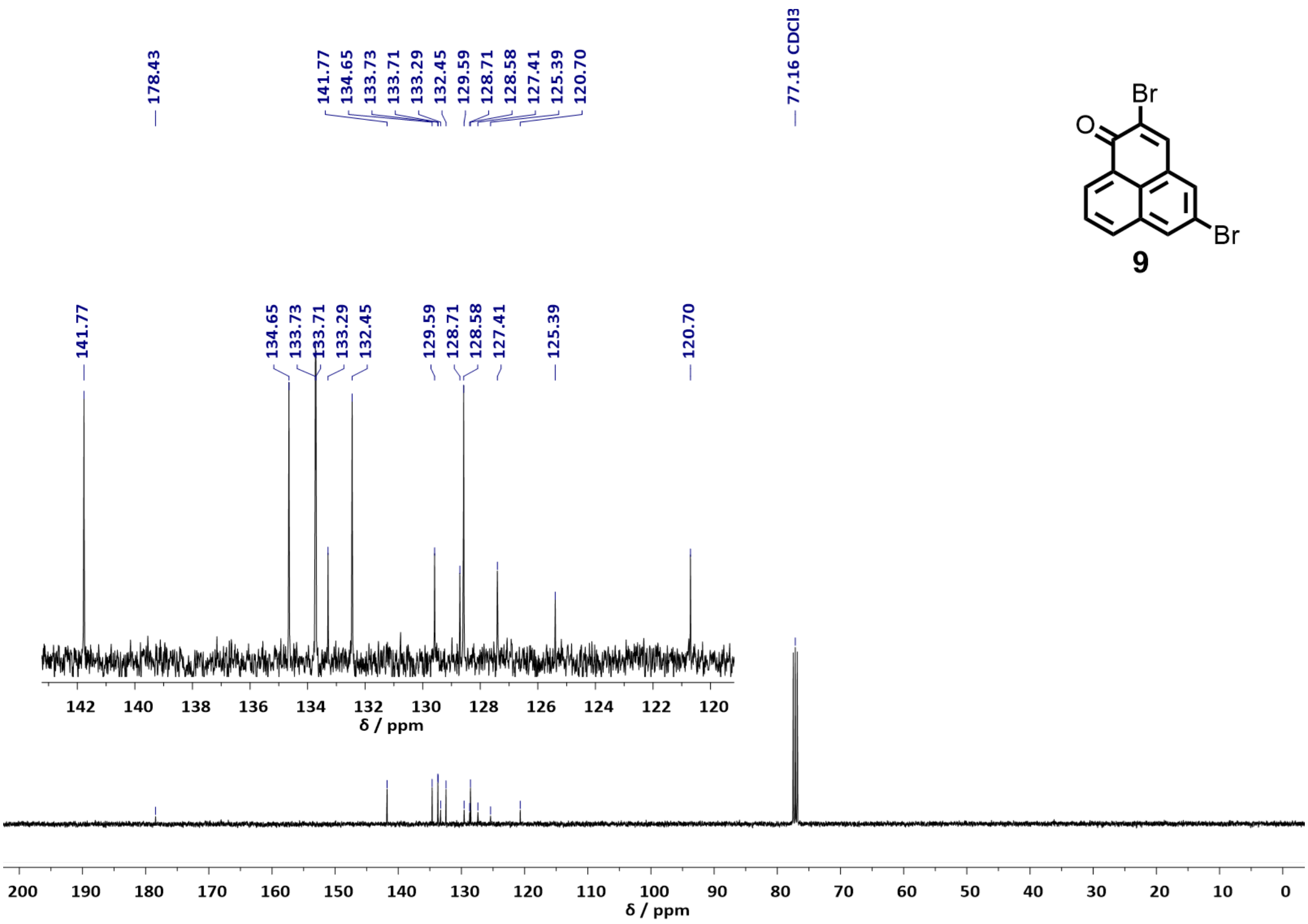

Figure S23. ^{13}C-NMR spectrum of **9** dissolved in $CDCl_3$, 101 MHz, 298 K.

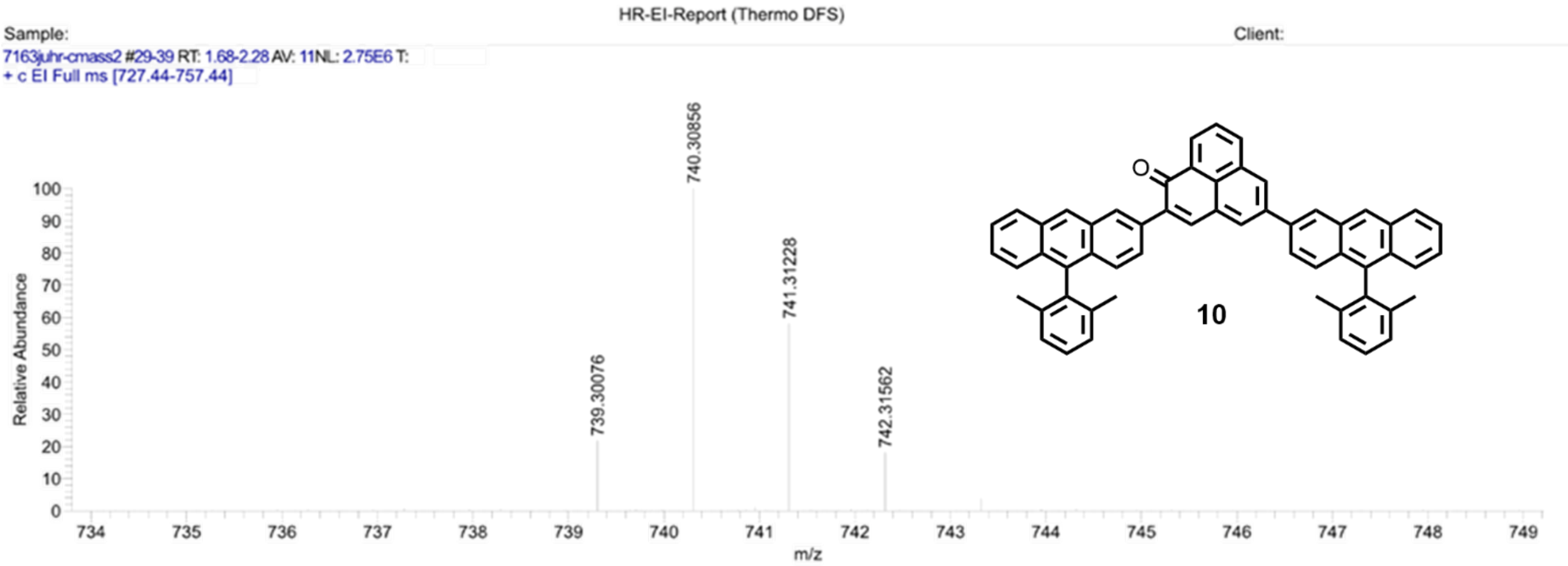

7163juhr-cmass2#29-39 RT: 1.68-2.28 AV: 11 T: +
c EI Full ms [727.44-757.44]
m/z= 738.93351-740.52882

m/z	Intensity	Relative	Theo. Mass	Delta (ppm)	Composition
739.30076	597395.4	21.75	739.29954	1.65	$C_{57}H_{39}O$
740.30856	2746536.6	100.00	740.30737	1.61	$C_{57}H_{40}O$

Figure S24. HR-EI-MS report of compound **10**.

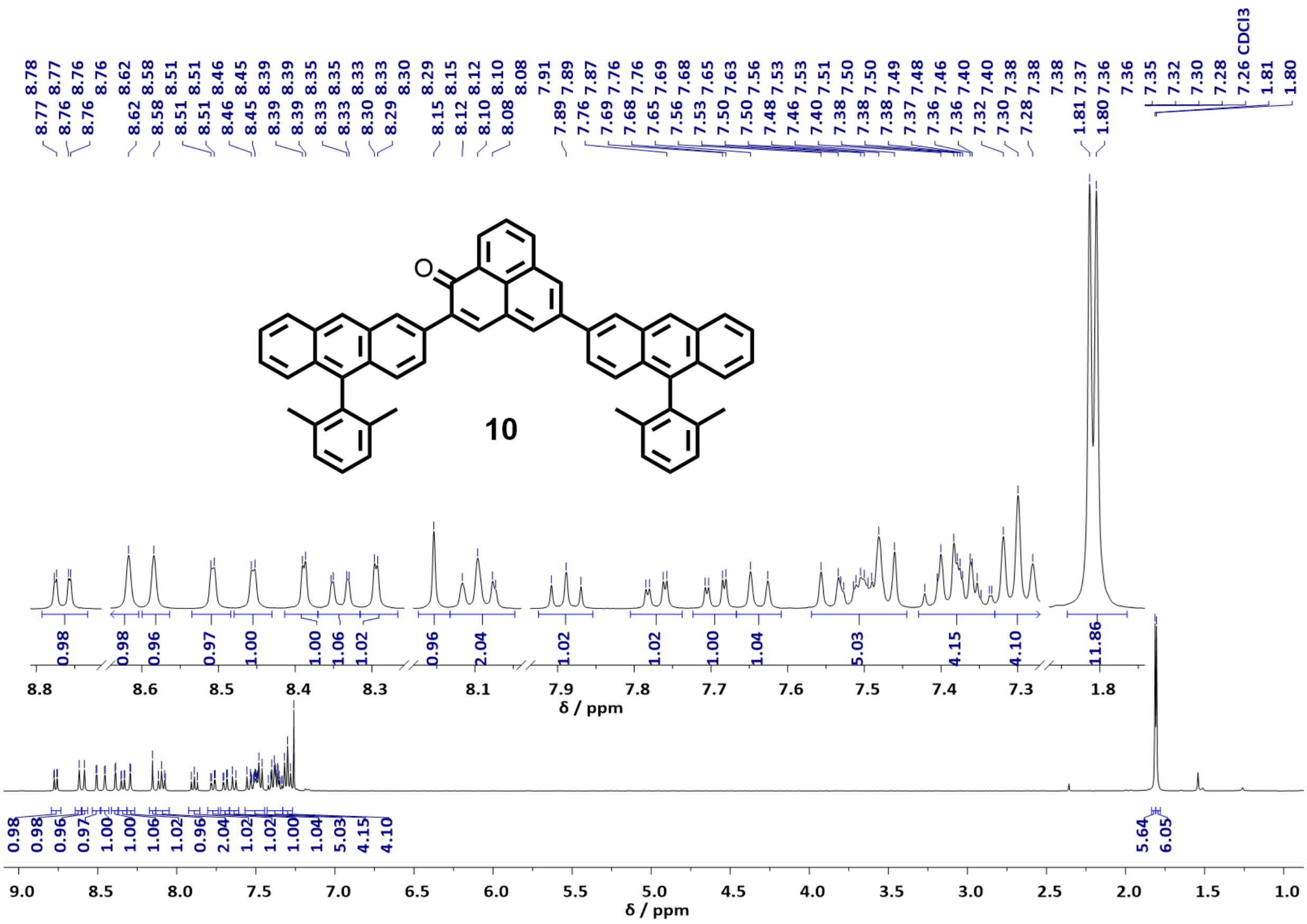

Figure S25. ^{1}H-NMR spectrum of **10** dissolved in $CDCl_3$, 400 MHz, 298 K.

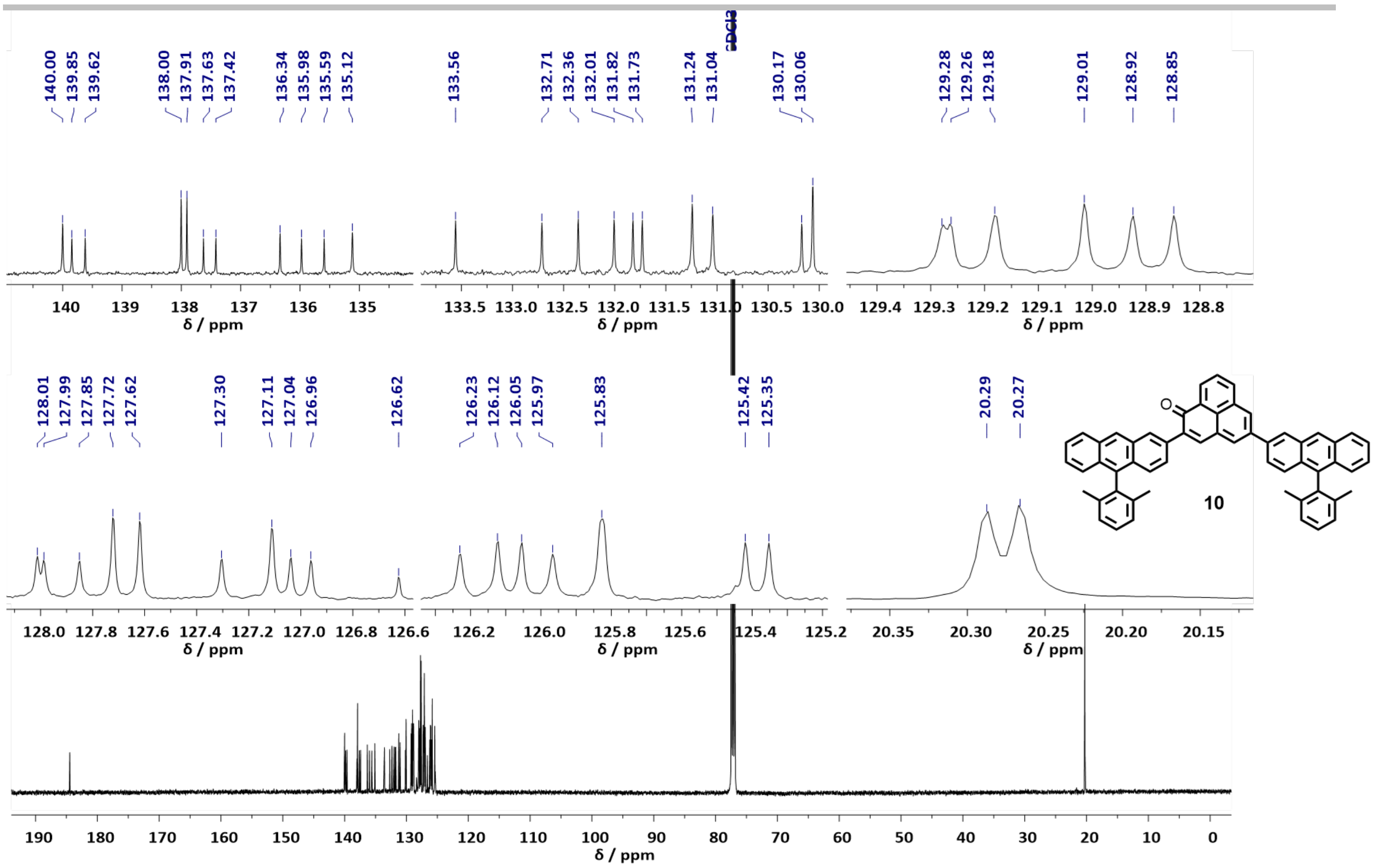

Figure S26. ^{13}C-NMR spectrum of **10** dissolved in $CDCl_3$, 101 MHz, 298 K (four carbon resonances could not be resolved because of signal overlap).

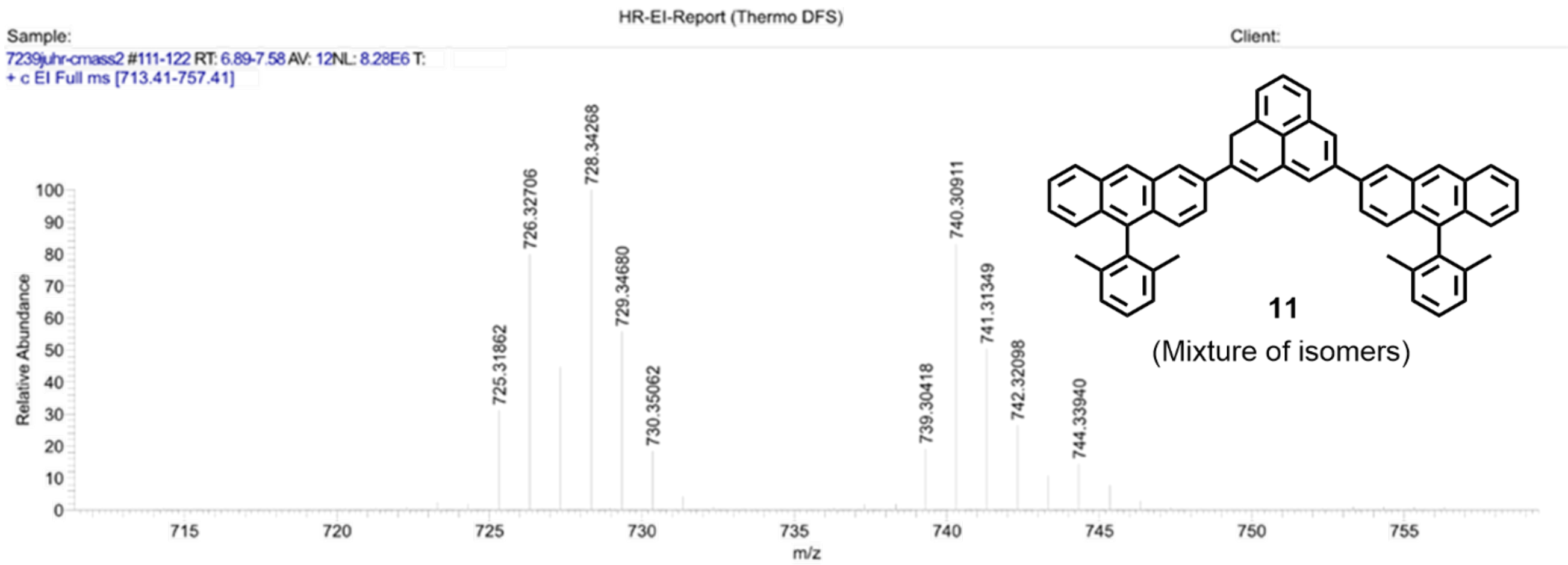

7239juhr-cmass2#111-122 RT: 6.89-7.58 AV: 12 T: +
c EI Full ms [713.41-757.41]
m/z= 711.41143-759.41143

m/z	Intensity	Relative	Theo. Mass	Delta (ppm)	Composition
726.32706	6618112.4	79.90	726.32810	-1.44	$C_{57}H_{42}$
728.34268	8283150.5	100.00	728.34375	-1.47	$C_{57}H_{44}$
740.30911	6887956.1	83.16	740.30737	2.36	$C_{57}H_{40}O$

Figure S27. HR-EI-MS report of compound **11**.

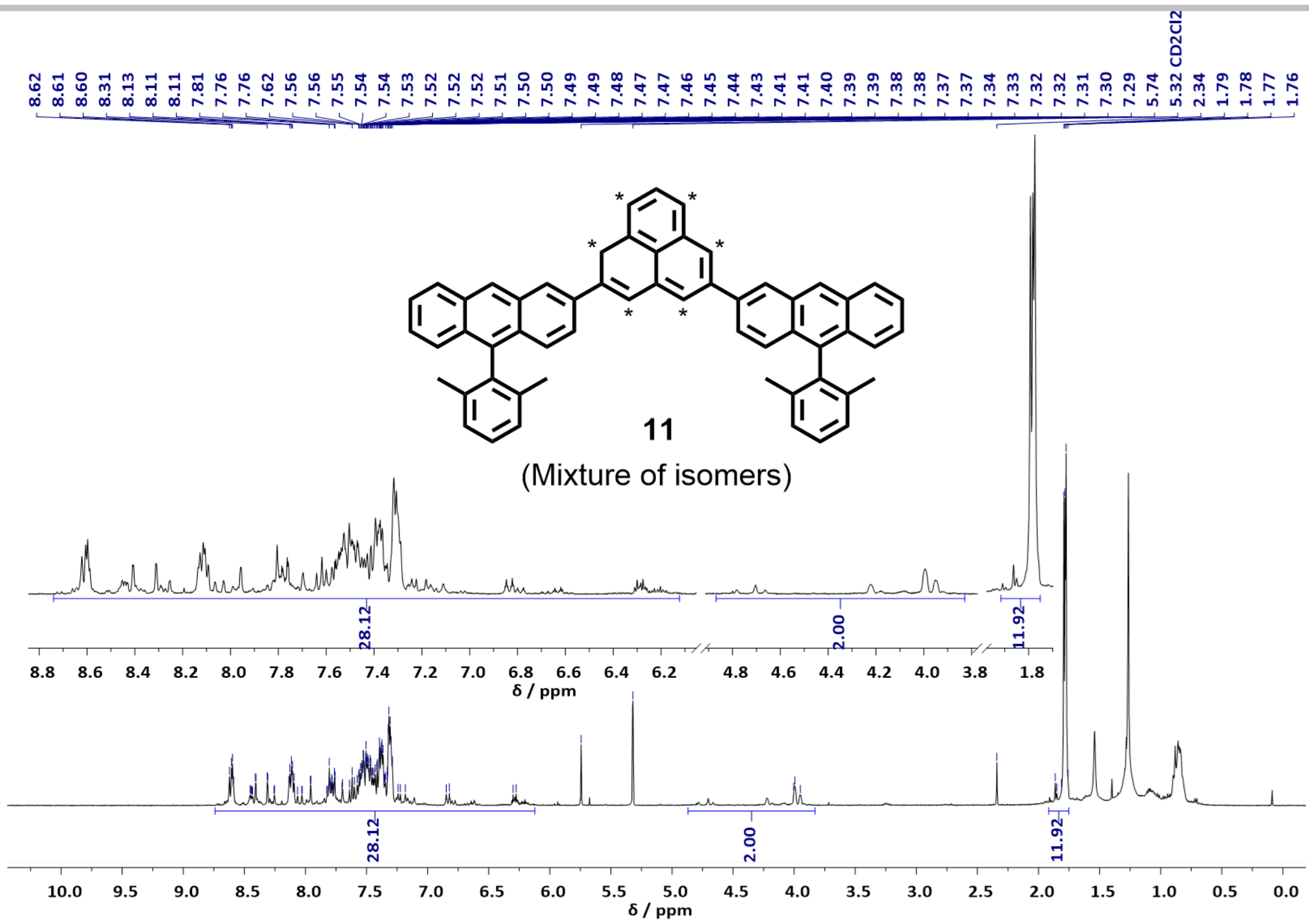

Figure S28. ^{1}H-NMR spectrum of **11** dissolved in CD_2Cl_2, 400 MHz, 298 K.

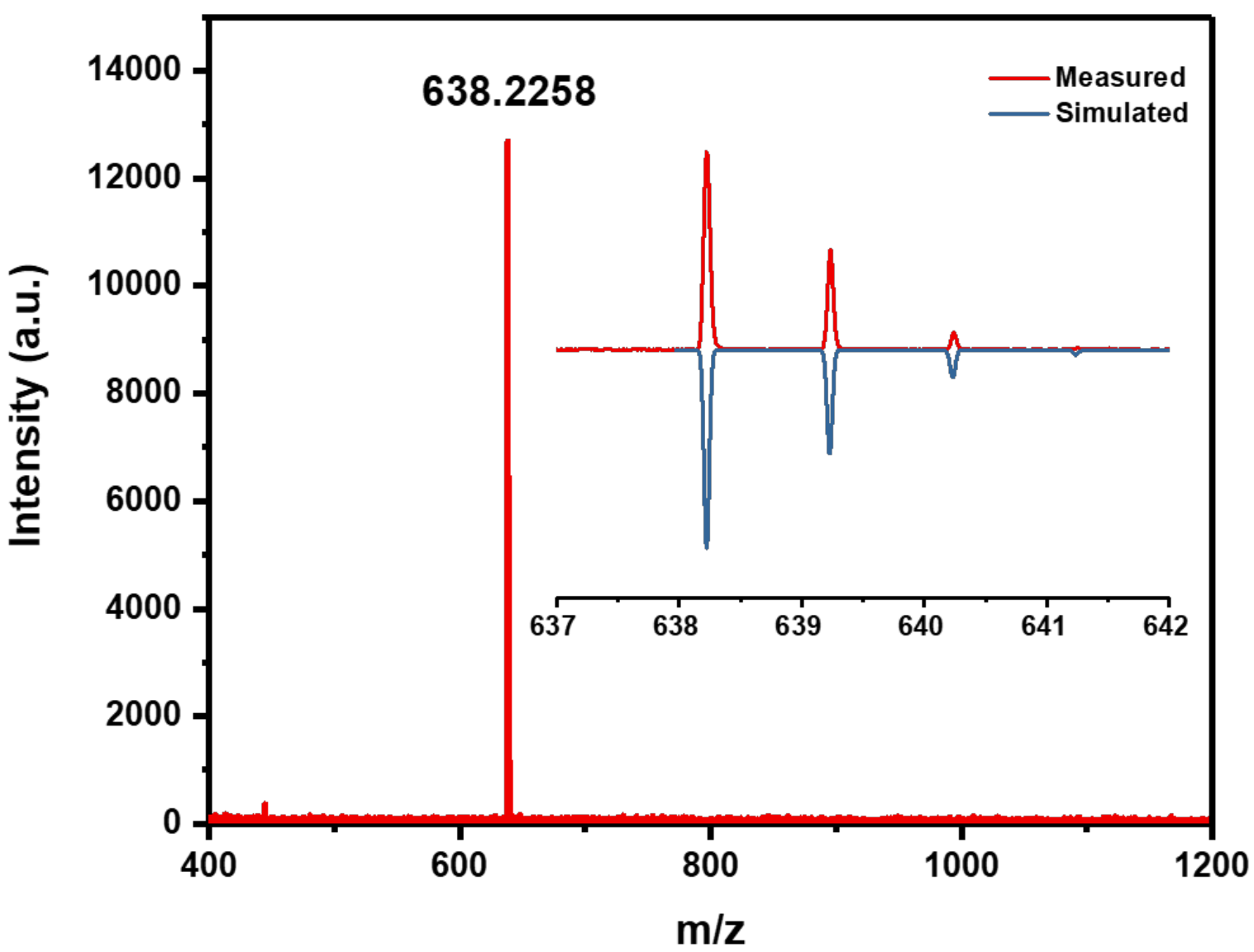

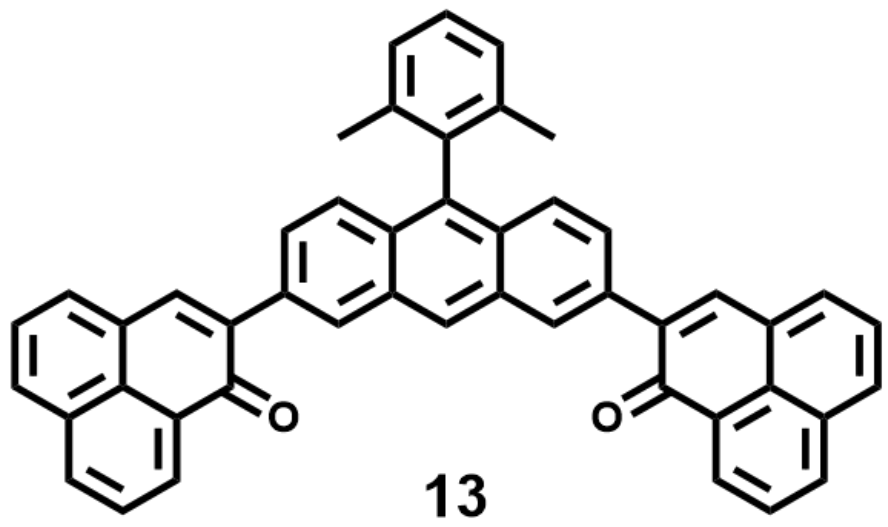

13

Figure S29. Liquid-state HR-MALDI-TOF-MS spectrum of compound **13** (matrix: DCTB).

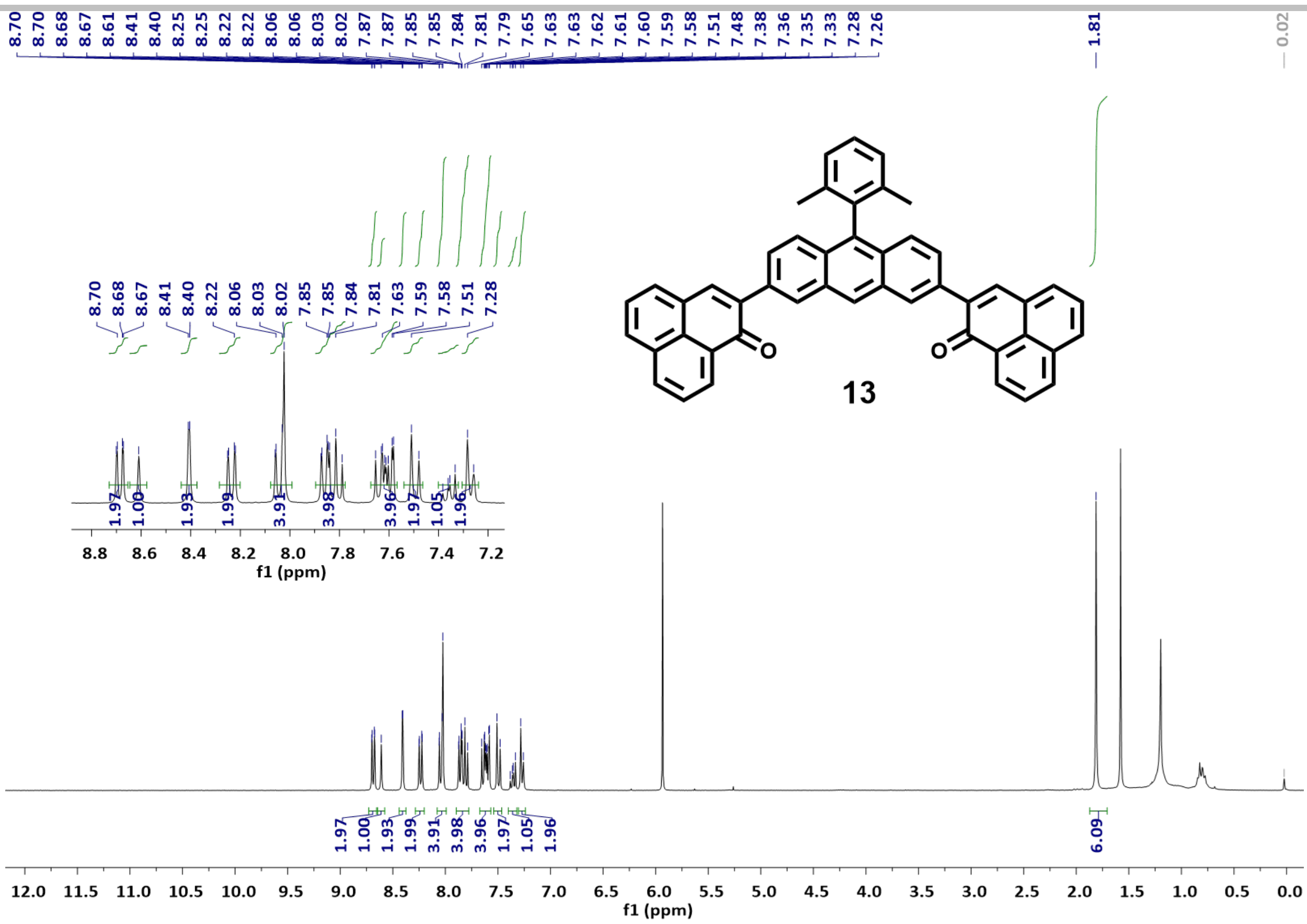

Figure S30. ^{1}H-NMR spectrum of compound **13** dissolved in 1,1,2,2-tetrachloroethane-d_2, 300 MHz, 296 K.

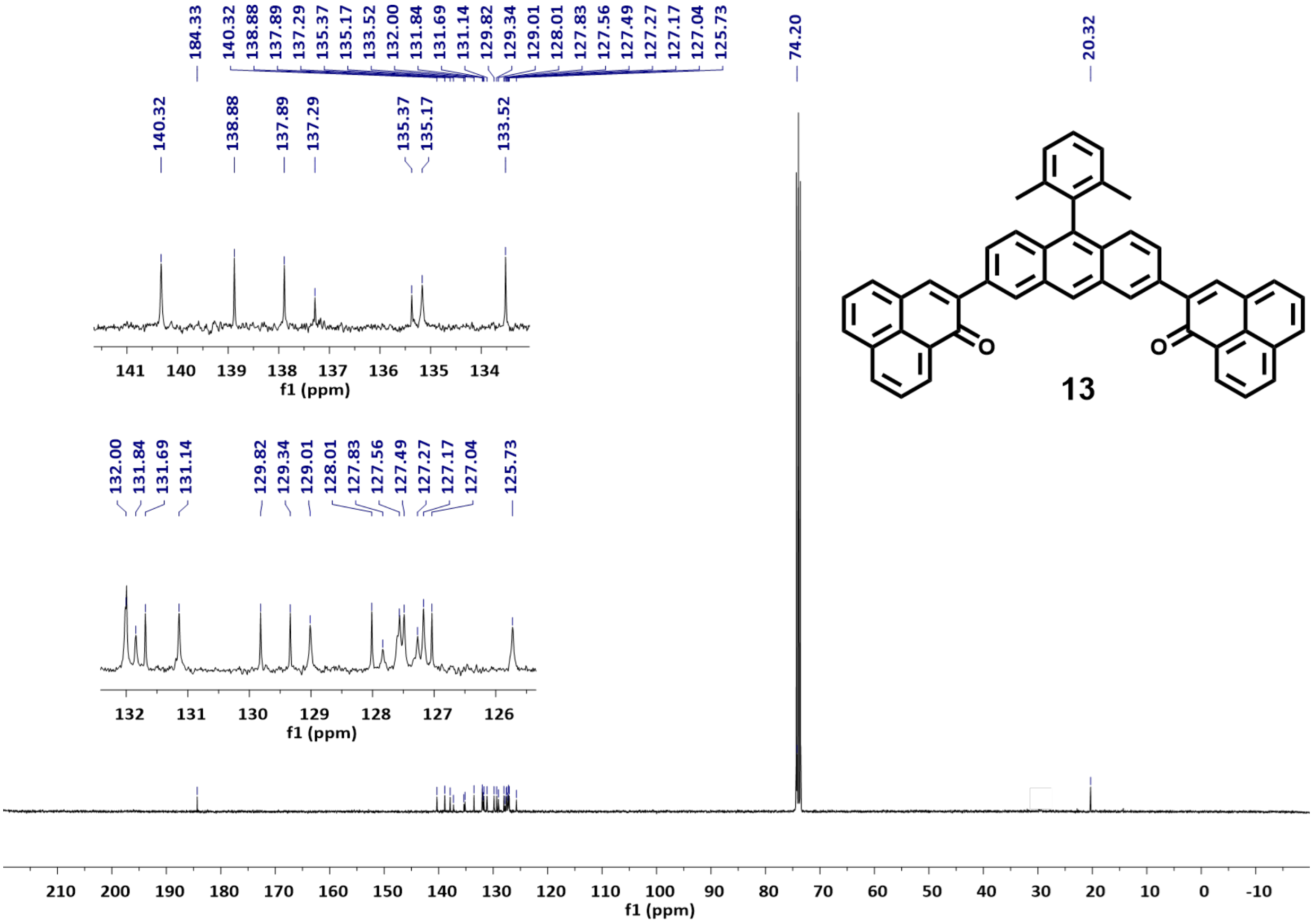

Figure S31. ^{13}C-NMR spectrum of **13** dissolved in 1,1,2,2-tetrachloroethane-d_2, 75 MHz, 296 K (one carbon resonance could not be resolved because of signal overlap).

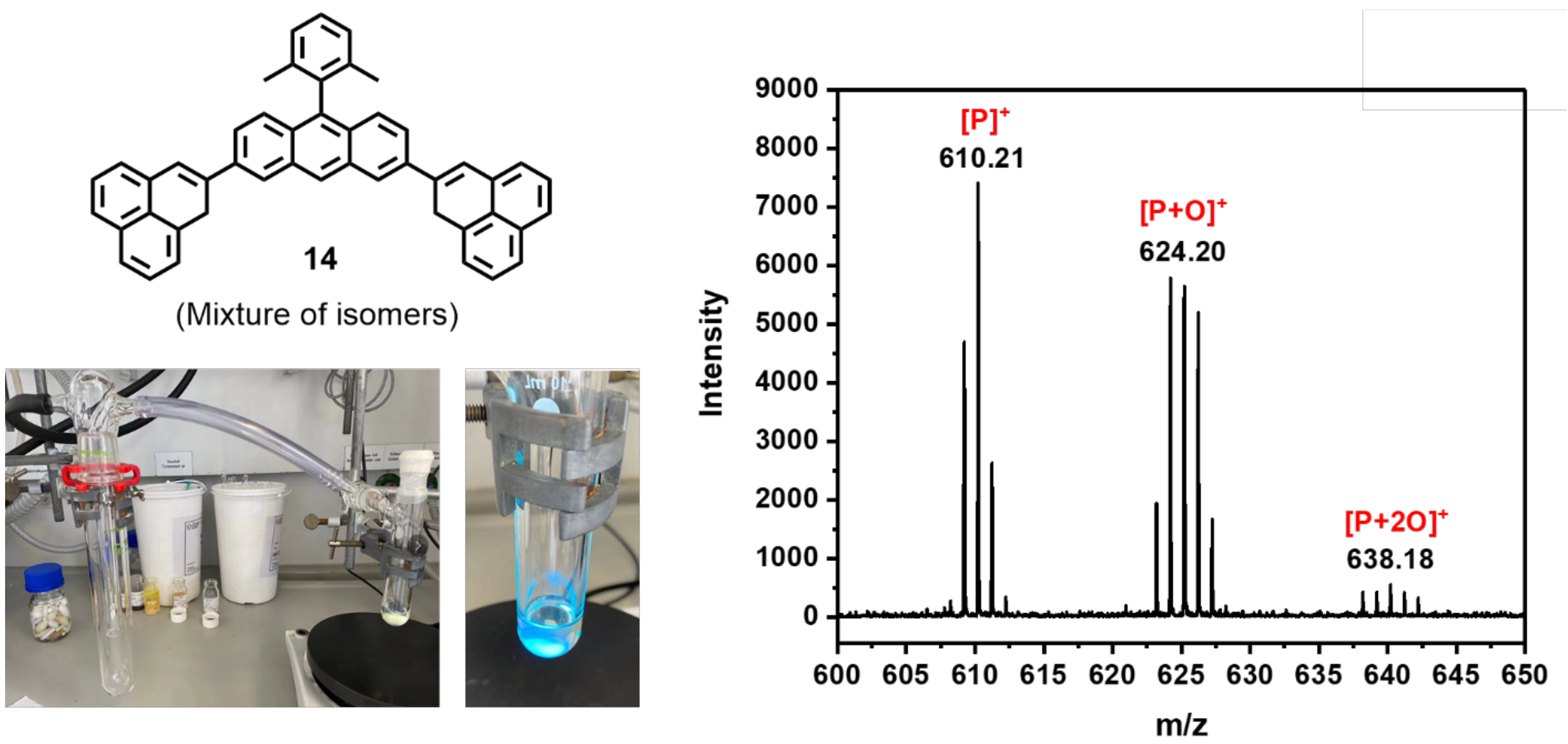

Figure S32. Method A: Experimental setup for the reduction reaction leading to compound **14**, along with the MALDI-TOF mass spectrum of the crude product (matrix: dithranol)..

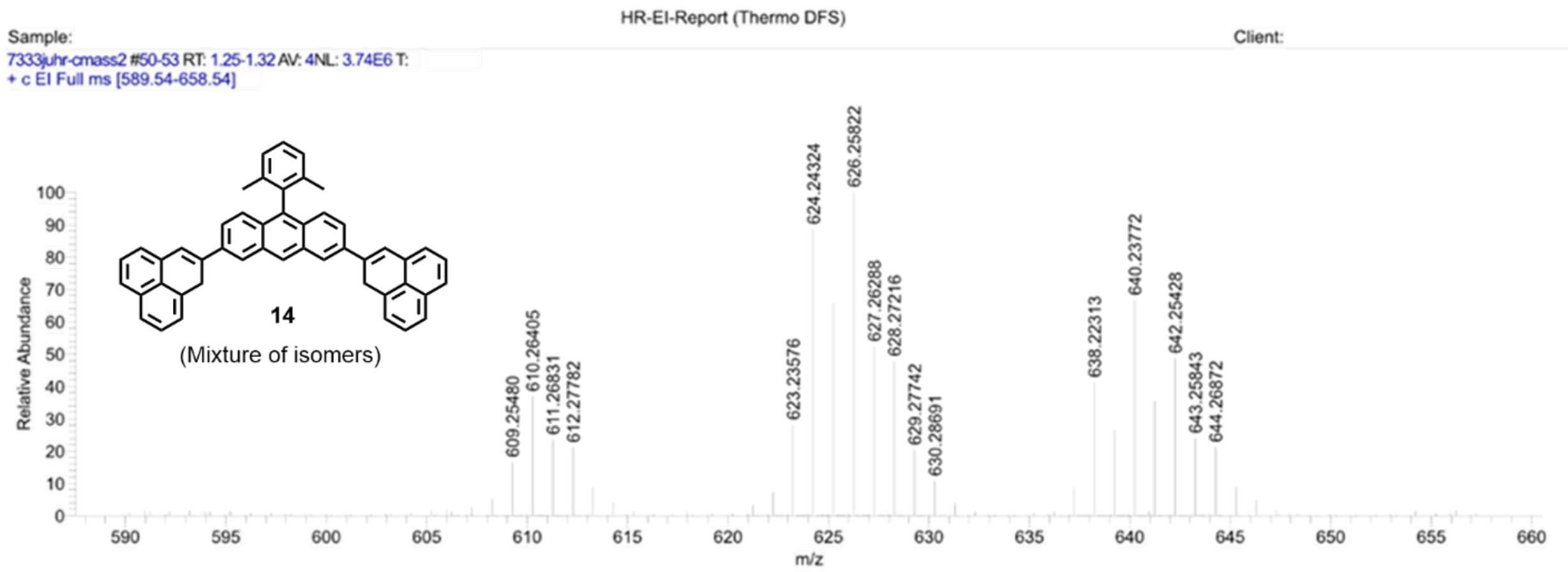

7333juhr-cmass2 #50-53 RT: 1.25-1.32 AV: 4 T: +
c EI Full ms [589.54-658.54]
m/z= 608.08949-642.79808

m/z	Intensity	Relative	Theo. Mass	Delta (ppm)	Composition
610.26405	1379515.8	36.89	610.26550	-2.39	$C_{48}H_{34}$
622.22816	269810.5	7.21	622.22912	-1.54	$C_{48}H_{30}O$
623.23576	1053821.3	28.18	623.23694	-1.90	$C_{48}H_{31}O$
624.24324	3312122.3	88.56	624.24477	-2.44	$C_{48}H_{32}O$
626.25822	3739882.3	100.00	626.26042	-3.51	$C_{48}H_{34}O$
638.22313	1544216.8	41.29	638.22403	-1.41	$C_{48}H_{30}O_2$
640.23772	2492057.8	66.63	640.23968	-3.07	$C_{48}H_{32}O_2$
642.25428	1819341.0	48.65	642.25533	-1.64	$C_{48}H_{34}O_2$

Figure S33. Method B: HR-EI-MS report of compound **14**.

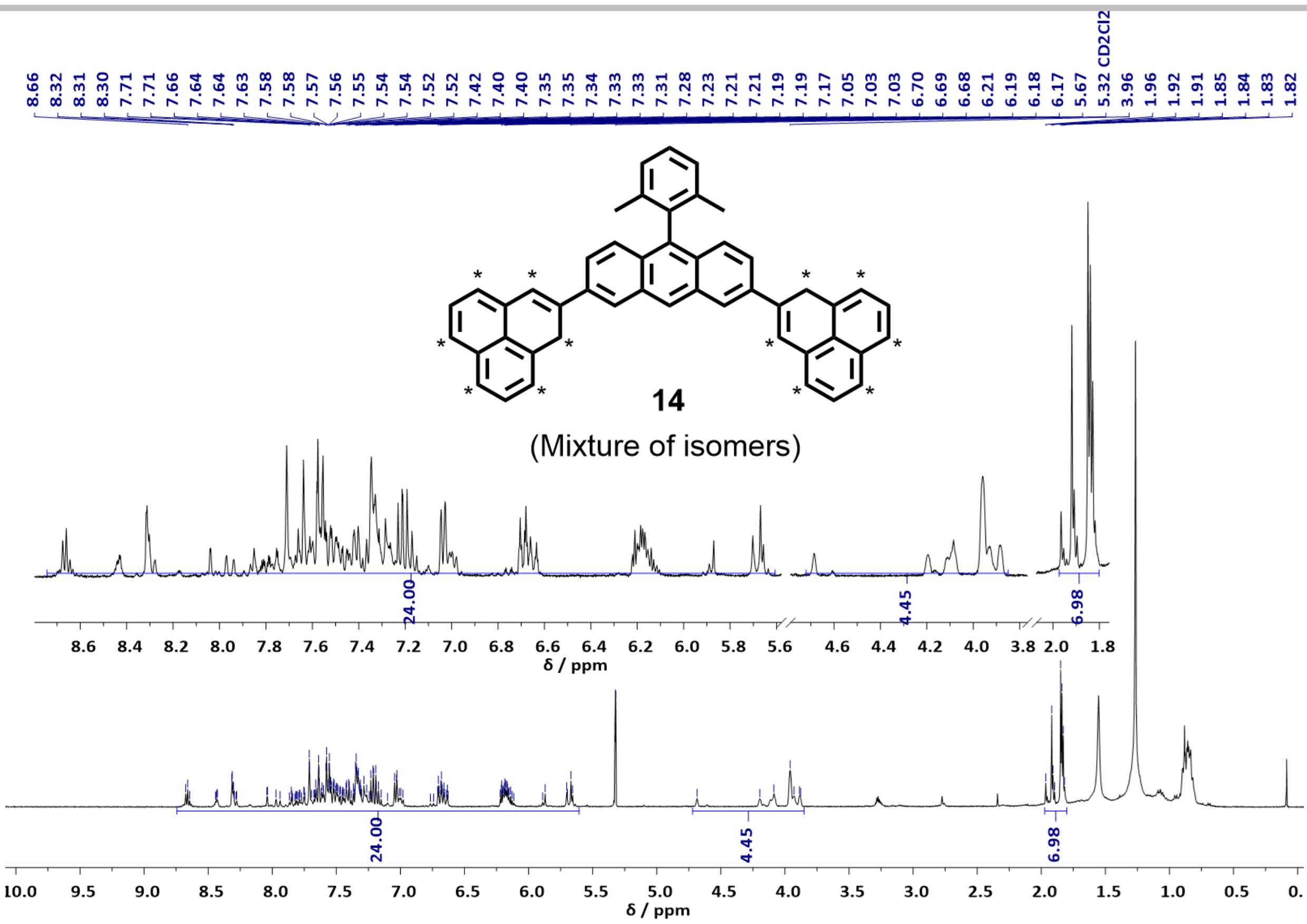

Figure S34. ^{1}H-NMR spectrum of **14** dissolved in CD_2Cl_2, 400 MHz, 298 K.

References

[5] S. Mishra, G. Catarina, F. Wu, R. Ortiz, D. Jacob, K. Eimre, J. Ma, C. A Pignedoli, X. Feng, P. Ruffieux, J. Fernández-Rossier, R Fasel, *Nature* **2021**, *598*, 287–292.

[6] A. Bernhardt, D. Čavlović, M. Mayländer, O. Blacque, C. M. Cruz, S. Richert, M. Juríček, *Angew. Chem. Int. Ed*. **2024**, *63*, e202318254.

[7] T. Ishiyama, M. Murata, N. Miyaura, *J. Org. Chem*. **1995**, *60*, 7508-7510.

[8] W. Hidalgo, L. Duque, J. Saez, R. Arango, J. Gil, B. Rojano, B. Schneider, F. Otálvaro, *J. Agric. Food Chem*. **2009**, *57*, 7417–7421.

[9] S. E. Webber, R. J. Almassy, *IMMUNE CHECKPOINT INHIBITORS, COMPOSITIONS AND METHODS THEREOF*, **2018**, US2018/65917.

[10] P. Boudjouk, P. D. Johnson, *J. Org. Chem.* **1978**, *43*, 3979–3980.
